# Two‐Stage Estimation for Causal Inference Involving Semi‐Continuous Exposures

**DOI:** 10.1002/sim.70800

**Published:** 2026-10-28

**Authors:** Xiaoya Wang, Richard J. Cook, Yeying Zhu, Tugba Akkaya‐Hocagil, R. Colin Carter, Sandra W. Jacobson, Joseph L. Jacobson, Louise M. Ryan

**Affiliations:** ^1^ Department of Statistics and Actuarial Science University of Waterloo Waterloo Ontario Canada; ^2^ Department of Biostatistics, School of Medicine Ankara University Ankara Turkey; ^3^ Departments of Emergency Medicine and Pediatrics Columbia University New York New York USA; ^4^ Department of Psychiatry and Behavioral Neurosciences Wayne State University Detroit Michigan USA; ^5^ School of Mathematical and Physical Sciences University of Technology Sydney Ultimo New South Wales Australia

**Keywords:** augmented inverse probability weighting, causal inference, inverse probability weighting, propensity score regression adjustment, semi‐continuous exposure

## Abstract

Methods for causal inference are well developed for binary and continuous exposures, but in many settings, the exposure has a substantial mass at zero—such exposures are called semi‐continuous. We propose a general causal framework for such semi‐continuous exposures, together with a two‐stage estimation strategy. A two‐part propensity structure is introduced for the semi‐continuous exposure, with one component for exposure status (exposed vs unexposed) and another for the exposure level among those exposed, and incorporates both into a marginal structural model that disentangles the effects of exposure status and dose. The two‐stage procedure sequentially targets the causal dose–response among exposed individuals and the causal effect of exposure status at a reference dose, allowing flexibility in the choice of propensity score methods in the second stage. We establish consistency and asymptotic normality for the resulting estimators, and characterize their limiting values under misspecification of the propensity score models. Simulation studies evaluate finite sample performance and robustness, and an application to a study of prenatal alcohol exposure and child cognition demonstrates how the proposed methods can be used to address a range of scientific questions about both exposure status and exposure intensity.

**Abbreviations**
AIPW: augmented inverse probability weighting
IPW: inverse probability weighting
PAE: prenatal alcohol exposure
PS: propensity score

## 1 Introduction

### 1.1 Background

In many areas of public health research, the goal is to assess whether an exposure has a causal effect on an outcome and to quantify that effect. In observational studies, propensity score (PS) methods are now widely used to mitigate bias due to measured confounding variables [1, 2, 3]. For binary exposures, propensity scores are used for matching, stratification, and inverse probability weighting [4, 5]. For continuous exposures, the generalized PS is based on the conditional density of the exposure evaluated at the observed exposure level [6, 7]. Although inverse density weighting can be used to estimate causal dose‐response relations in principle, in practice, it can lead to highly variable estimators when extreme weights arise (e.g., due to outliers or limited overlap) [8]. Moreover, modeling the exposure distribution can be challenging in practice—discretizing the exposure or the generalized PS has been advocated by some [9].

In many applications, samples comprise both unexposed individuals with variation in the exposure level among exposed individuals. This results in an exposure distribution with a point mass at zero and a continuous component among the exposed. Semi‐continuous exposures are also common in behavioral settings—for example, smoking, cannabis use, or alcohol consumption—where many individuals abstain and the level of exposure varies among users. Semi‐continuous exposures also arise in environmental research where toxins may be absent in some locations, yet vary in concentration among locations where it is present [10]. There has been relatively limited methodological attention to causal analysis involving such exposure distributions.

A motivating problem involves the study of prenatal alcohol exposure (PAE) and its relation to child cognition. A substantial proportion of mothers abstain from drinking during pregnancy, while the intensity of consumption varies widely among drinkers, yielding a semi‐continuous exposure with a point mass at zero and a continuous right‐skewed distribution among the exposed. Although epidemiological studies provide evidence of an association between PAE and cognition [11, 12, 13, 14], the causal dose–response relation remains unclear. This motivates two causal targets: the dose–response effect among exposed individuals and the contrast between exposure at a clinically meaningful reference dose and no exposure.

Recent work has proposed two‐part PS strategies for semi‐continuous exposures by modeling exposure status and dose among exposed using separate regression models [15, 16]; however, these approaches are often presented primarily as modeling and adjustment tools and do not explicitly define and target both causal estimands within a unified potential outcomes framework, together with corresponding identification assumptions and large sample properties. In this article, we develop and evaluate a two‐stage framework using propensity scores for causal inference with a semi‐continuous exposure and a continuous outcome. The approach targets two causal estimands using separate PS models: in Stage I the dose‐response effect is estimated among exposed individuals using PS regression adjustment, and in Stage II, the effect of exposure at a specified reference dose versus no exposure is estimated using a second PS for exposure status. The two‐stage structure enables a use of an augmented inverse probability weighted (AIPW) estimating equation [17, 18] in Stage II. To reflect realistic data‐generating mechanisms, we allow distinct (but possibly overlapping) sets of confounders affecting exposure status and dose among the exposed. We formalize the approach in the potential outcomes framework, define interpretable causal estimands aligned with the semi‐continuous exposure distribution, and state identifying assumptions. We establish large sample properties for the resulting estimators, evaluate finite sample performance through extensive simulation studies, including settings involving PS misspecification, and illustrate the methods in an analysis of the effect of PAE and child cognition [19]. The methodological contributions of this article are threefold. First, we propose a two‐stage decomposition that targets the dose–response and reference‐dose causal effects as distinct estimands, each estimated with a propensity score model tailored to its corresponding exposure component—the continuous dose among the exposed in Stage I, and the binary exposure status in Stage II. This avoids the need for a single joint model linking the binary and continuous parts of the exposure, and accommodates settings in which the confounders affecting exposure status differ from those affecting dose. Second, Stage I enters Stage II through an offset construction: the fitted dose–response model in Stage I is subtracted from the outcome to form a pseudo‐outcome that isolates the reference‐dose effect from the continuous dose–response. This construction supports sequential estimation while explicitly propagating Stage I uncertainty into Stage II through a joint sandwich variance estimation. Third, when the Stage I propensity score is correctly specified, the Stage II AIPW estimator remains consistent if either the Stage II propensity score for exposure status or the outcome imputation model is correctly specified, but not necessarily both. This clarifies precisely which model components are critical for valid inference at each stage and motivates the misspecification scenarios examined in our simulation studies.

The remainder of this article is organized as follows: In Section 1.2, we introduce the motivating PAE study. In Section 2, we introduce notation, models and identification assumptions for causal analysis involving a semi‐continuous exposure. Regression adjustment based on a two‐part PS is introduced to estimate the two causal estimands of interest. In Section 3, we present the two‐stage estimators for the binary status and continuous dose effects, and derive their asymptotic properties. In Section 4, we study the impact of propensity score misspecification by evaluating the limiting values of the estimators. Simulation studies are reported in Section 5, and in Section 6 we apply the methods to data from the Detroit study on the effect of PAE on child cognition. Conclusions and future directions of research are discussed in Section 7.

### 1.2 A Study of Prenatal Alcohol Exposure and Child Cognition

The Detroit longitudinal cohort study followed children born between 1986 and 1989 from birth through adolescence and early adulthood and has been used to examine the effects of PAE on neurocognitive outcomes [11, 19]. A total of 480 pregnant African‐American women were recruited from inner‐city areas and interviewed during pregnancy about alcohol use using a timeline follow‐back method. At each interview, average daily alcohol consumption during pregnancy was recorded and expressed as ounces of ***AA per day.

We focus on the 377 children who completed the 7‐year follow‐up and use the Full Scale IQ (FSIQ) score from the Wechsler Intelligence Scale for Children, Third Edition (WISC‐III), as the outcome. The WISC‐III is a standardized measure of cognitive functioning for children aged 6–16 years that provides norm‐referenced IQ scores, including the Full Scale IQ summary measure [20]. The FSIQ score is norm‐referenced (mean = 100) and typically ranges from 40 to 160, with higher values indicating better overall cognitive performance relative to age‐matched peers [20]. Figure 1A displays the distribution of AA/day; 16.2% of mothers reported that they abstained from drinking during pregnancy with the right‐skewed distribution for the AA/day among drinkers. Figure 1B shows the corresponding log‐transformed drinking dose, among the 316 drinkers. This distribution motivates two causal targets: the dose–response effect among mothers who reported drinking and the effect of a clinically meaningful non‐zero exposure level relative to no exposure, which are addressed by the two‐stage PS framework developed in the following sections.

**FIGURE 1:**
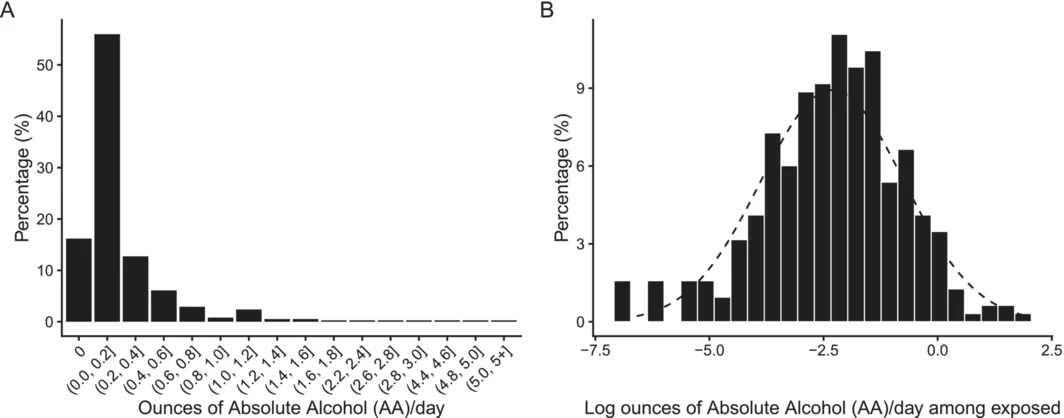
Distribution of PAE in the Detroit longitudinal cohort study. (A) Ounces of absolute alcohol (AA)/day for all 377 mothers, showing a point mass at zero (16.2% non‐drinkers) and a right‐skewed continuous component among drinkers. (B) Log dose, D=logT, among the 316 exposed mothers, with a fitted normal density (dashed); the log transformation yields an approximately symmetric distribution.

## 2 Causal Analysis Under a Semi‐Continuous Exposure

### 2.1 Notation, Potential Outcomes, and Causal Estimands

Let Y be a continuous response variable, T be a non‐negative random variable representing the exposure dose (e.g., the ounces of AA per day), and A=I(T>0) indicate the exposure status. Let $X={\left(X_{1}^{\prime },X_{2}^{\prime },X_{3}^{\prime }\right)}^{\prime }$ be a vector of three sets of confounders with $X_{1}={\left(X_{11},\ldots ,X_{1k_{1}}\right)}^{\prime }$, $X_{2}={\left(X_{21},\ldots ,X_{2k_{2}}\right)}^{\prime }$, and $X_{3}={\left(X_{31},\ldots ,X_{3k_{3}}\right)}^{\prime }$. $X_{1}$ and $X_{3}$ confound the T−Y association given A=1, and $X_{2}$ and $X_{3}$ confound the A−Y association; see the directed acyclic graph in Figure 2. In practice, $X_{1}$, $X_{2}$ and $X_{3}$ could be the same set of covariates, but we use separate notations here to allow them to vary and represent their different roles with respect to confounding. Let $Z_{1}={\left(X_{1}^{\prime },X_{3}^{\prime }\right)}^{\prime }$ and $Z_{2}={\left(X_{2}^{\prime },X_{3}^{\prime }\right)}^{\prime }$ denote the confounders of the T–Y and A–Y associations, respectively. For a sample of n independent mother‐child pairs, the observed data is denoted as $\left\{(Y_{i},T_{i},A_{i},X_{i}^{\prime }),i=1,\ldots ,n\right\}$, where $Z_{i1}$ and $Z_{i2}$ are defined from $X_{i}$ accordingly.

**FIGURE 2:**
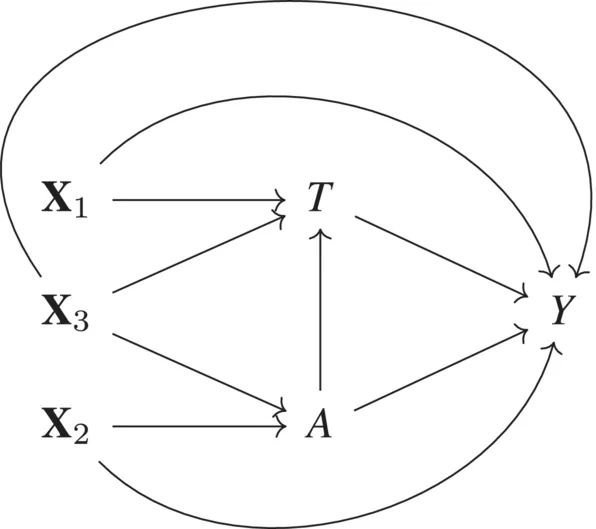
A directed acyclic graph for a two‐part exposure model.

As is common in dose‐response modelling, we consider a log transformation of the continuous exposure T if T>0 and refer to D=logT as the dose, with d its realized value. We let $Y_{i}(1,d)$ represent the potential outcome for the i‐th individual exposed at dose d [1, 21]; if the i‐th individual is unexposed, then the dose is undefined, but we denote the potential outcome as $Y_{i}(0,0)$ for consistency of notation, taking it as understood that d is undefined. The semi‐continuous nature of the exposure motivates two causal estimands. First, among exposed individuals we consider the causal dose‐response effect

(1) $$\Delta_{D}=E\{Y(1,d+1)-Y(1,d)|A=1\}.$$

Conditioning on A=1 defines the target population as individuals who are exposed in the observed population. Second, to compare exposed versus unexposed individuals, we must specify a reference dose c and consider this reference dose as applying to the exposed when assessing the effect of exposure (exposed versus unexposed). Thus, we define

(2) $$\Delta_{0}(c)=E\{Y(1,c)-Y(0,0)\}$$

as one causal effect of interest. The quantity c in (2) is a user‐chosen reference dose that indexes a family of causal contrasts $\left\{\Delta_{0}(c):c\in 𝒞\right\}$. For each c, $\Delta_{0}(c)$ represents the effect of setting the doses of exposure to c in exposed individuals versus no exposure. Thus, changing c changes the causal estimand by design: different values of c correspond to different, but well‐defined causal estimands (e.g., abstinence (“no drinking”) versus drinking at a low‐risk guideline level, or versus drinking at the mean dose among exposed individuals). It would be good practice to specify c based on substantive considerations, such as regulatory thresholds or typical exposure levels.

To provide a summary of the two causal targets in (1) and (2), we consider a marginal structural model (MSM) involving a semi‐continuous exposure as

(3) $$E\{Y(a,d)\}=\psi_{0}+\psi_{11}a(d-c)+\psi_{12}a.$$

The terms a(d−c) and a ensure that the dose component contributes only for exposed individuals; when a=0 both terms drop out and the mean reduces to $E\{Y(0,0)\}=\psi_{0}$. Under (3), $\Delta_{0}(c)=\psi_{12}$ and, for the exposed target population, $\Delta_{D}(d_{1},d_{0})=\psi_{11}(d_{1}-d_{0})$, so $\psi_{12}$ and $\psi_{11}$ directly parameterize the reference‐dose effect and the dose–response effect, respectively. Note that (3) can be written as

$$E\{Y(a,d)\}=\psi_{0}+\psi_{11}ad-\psi_{11}ac+\psi_{12}a.$$

So if we choose an alternative reference level $c^{\prime }$ and write

$$E\{Y(a,d)\}=\psi_{0}^{\prime }+\psi_{11}^{\prime }a(d-c^{\prime })+\psi_{12}^{\prime }a,$$

then equality of the two parameterizations for all (a,d) implies $\psi_{11}^{\prime }=\psi_{11}$ and $\psi_{12}^{\prime }=\psi_{12}+\psi_{11}(c^{\prime }-c)$. Thus, changing c leaves the dose–response slope $\psi_{11}$ and the model's fitted values unchanged; it only re‐centers the reference level, shifting the exposed group's intercept $\psi_{12}$ according to $\psi_{12}^{\prime }=\psi_{12}+\psi_{11}(c^{\prime }-c)$. Here $\psi_{12}$ is an intercept in the sense that, writing the exposed mean as $E\{Y(1,d)\}=\psi_{0}+\psi_{12}+\psi_{11}(d-c)$, it gives the level of the exposed regression line at the reference dose d=c relative to the unexposed baseline $\psi_{0}$; equivalently, $\psi_{12}=E\{Y(1,c)\}-E\{Y(0,0)\}=\Delta_{0}(c)$. In this sense, the choice of c is analogous to centering a covariate in a linear regression model: it determines which particular contrast $\Delta_{0}(c)$ is reported, but does not change the underlying dose–response relation among the exposed. The assumptions in Section 2.2 ensure that these parameters (and hence $\Delta_{0}(c)$ and $\Delta_{D}$) are causally interpretable and identifiable from the observed data.

### 2.2 Identification Assumptions

We next state assumptions for identifiability the semi‐continuous exposure setting [2, 22, 23, 24].

Assumption 1 (Stable Unit Treatment Value Assumption) There is no interference between individuals and consistency holds: $Y_{i}=Y_{i}(0,0)$ if $A_{i}=0$, and $Y_{i}=Y_{i}(1,d)$ if $A_{i}=1$ and $D_{i}=d$.

Assumption 2 (Exchangeability) For all d, $Y_{i}(1,d)\;⊥\;D_{i}|(A_{i}=1,Z_{i1})$ and $\{Y_{i}(0,0),Y_{i}(1,d)\}\;⊥\;A_{i}|Z_{i2}$.

Assumption 3 (Positivity) For covariate values with positive density, $0<P(A_{i}=1|Z_{i2})<1$ and $f(D_{i}|A_{i}=1,Z_{i1})>0$ for all d in the dose range of interest.

Assumption 1 links the observed outcome to the corresponding potential outcome under the observed exposure and rules out interference between individuals. Assumption 2 states that, conditional on measured covariates, exposure status is independent of the potential outcomes and, among exposed individuals, dose is independent of the potential outcomes. Assumption 3 requires overlap in exposure status across covariate values and adequate support for the dose levels needed to identify the dose‐response effect among exposed and the reference‐dose effect. Under Assumptions 1 to 3, $\psi_{11}$ identifies $\Delta_{D}$ and $\psi_{12}$ identifies $\Delta_{0}(c)$.

### 2.3 Model Settings and a Two‐Part Propensity Score

For the data generating model, we adopt a linear regression model relating the semi‐continuous exposure and baseline covariates to the conditional mean of the outcome,

(4) $$E\left(Y_{i}|A_{i},D_{i},X_{i};\theta \right)=\theta_{0}+\theta_{11}A_{i}(D_{i}-c)+\theta_{12}A_{i}+X_{i}^{\prime }\theta_{2},$$

where c is a pre‐specified reference dose, $\theta ={\left(\theta_{0},\theta_{11},\theta_{12},\theta_{2}^{\prime }\right)}^{\prime }$ with $\theta_{2}={\left(\theta_{21}^{\prime },\theta_{22}^{\prime },\theta_{23}^{\prime }\right)}^{\prime }$ and $\theta_{21}={\left(\theta_{211},\ldots ,\theta_{21k_{1}}\right)}^{\prime }$, $\theta_{22}={\left(\theta_{221},\ldots ,\theta_{22k_{2}}\right)}^{\prime }$ and $\theta_{23}={\left(\theta_{231},\ldots ,\theta_{23k_{3}}\right)}^{\prime }$. More generally, the conditional mean model in (4) can be enriched to allow for treatment–covariate interactions and effect modification. Let $V_{i}$ denote a vector of potential effect modifiers, typically a subset of $X_{i}$, and consider the extended specification

(5) $$\begin{array}{cc} & E\{Y_{i}|A_{i},D_{i},X_{i};\theta \}=\theta_{0}+\theta_{11}A_{i}(D_{i}-c)+\theta_{12}A_{i}+X_{i}^{\prime }\theta_{2}\\ & \;+A_{i}(D_{i}-c)V_{i}^{\prime }\theta_{3}+A_{i}V_{i}^{\prime }\theta_{4}. \end{array}$$

Under this specification, both the dose–response effect among exposed individuals and the reference‐dose exposure effect may vary across levels of $V_{i}$. The corresponding causal effects can therefore be summarized either marginally (with caveats) or within subgroups defined by $V_{i}$.

Separately, the baseline covariate adjustment term in (4) can be made more flexible by replacing $X_{i}$ with a vector of functions of the covariates, denoted $W(X_{i})$. This vector may include main effects, squared terms, and interactions among baseline covariates. The results in Sections 2 to 4 continue to apply when such a richer $W(X_{i})$ is used in place of $X_{i}$, since this amounts to enlarging the set of baseline covariate adjustment terms. This more general class of models is distinct from those used to accommodate effect modification through treatment–covariate interaction terms; the latter changes the causal effects being summarized. Here, we state the theory using the simpler model (4). The impact of effect modification on finite‐sample performance is examined via simulation in the heterogeneous treatment effect setting of Section 5.4.

To represent the semi‐continuous exposure distribution, we adopt a two‐part exposure model with one component for the continuous dose among the exposed and one for exposure status [15, 16]. Among exposed individuals, we specify a linear model for the continuous dose conditional on covariates,

(6) $$E(D_{i}|A_{i}=1,Z_{i1};\alpha_{1})={\bar{Z}}_{i1}^{\prime }\alpha_{1},$$

where ${\bar{Z}}_{1}={\left(1,Z_{1}^{\prime }\right)}^{\prime }$ and $\alpha_{1}={\left(\alpha_{10},\alpha_{11}^{\prime },\alpha_{12}^{\prime }\right)}^{\prime }$ where $\alpha_{11}={\left(\alpha_{111},\ldots ,\alpha_{11k_{1}}\right)}^{\prime }$ and $\alpha_{12}={\left(\alpha_{121},\ldots ,\alpha_{12k_{3}}\right)}^{\prime }$. For the exposure status, we define a logistic regression model

(7) $$\log\left\{\frac{P(A_{i}=1|Z_{i2})}{1-P(A_{i}=1|Z_{i2})}\right\}={\bar{Z}}_{i2}^{\prime }\alpha_{2},$$

with ${\bar{Z}}_{2}={\left(1,Z_{2}^{\prime }\right)}^{\prime }$ and $\alpha_{2}={\left(\alpha_{20},\alpha_{21}^{\prime },\alpha_{22}^{\prime }\right)}^{\prime }$ where $\alpha_{21}={\left(\alpha_{211},\ldots ,\alpha_{21k_{2}}\right)}^{\prime }$ and $\alpha_{22}={\left(\alpha_{221},\ldots ,\alpha_{22k_{3}}\right)}^{\prime }$.

For a binary exposure the PS is the conditional probability of exposure given covariates [2] while for continuous exposures the generalized PS is the conditional exposure density given covariates; more generally any function of the covariates that balances the exposure distribution can serve as a balancing score [7]. In our setting with a binary indicator A and a continuous dose D among the exposed, we construct a two‐part PS $S(X;\alpha )={\left(S_{1}(Z_{1};\alpha_{1}),\;S_{2}(Z_{2};\alpha_{2})\right)}^{\prime }$, where $S_{1}(Z_{1};\alpha_{1})=E(D|A=1,Z_{1};\alpha_{1})$ and $S_{2}(Z_{2};\alpha_{2})=P(A=1|Z_{2};\alpha_{2})$. Under the dose model in (6) and assuming normal errors with constant variance, $D|(A=1,Z_{1})∼N(S_{1}(Z_{1};\alpha_{1}),\sigma_{D}^{2})$, the conditional distribution of D depends on $Z_{1}$ only through $S_{1}(Z_{1};\alpha_{1})$; hence among exposed individuals, $D⊥Z_{1}|\{A=1,S_{1}(Z_{1};\alpha_{1})\}$. Note this normality assumption is stronger than correct specification of the conditional mean alone; when it is implausible, a density‐based generalized propensity score may be used [6, 7]. In our application, the log‐transformed dose among exposed individuals is approximately symmetric (Figure 1B), and, accordingly, S(X;α) can be treated as a two‐part PS for adjusting confounding in the semi‐continuous exposure setting under the exposure models (6) and (7).

In (6) and (7), the linear predictors ${\bar{Z}}_{i1}^{\prime }\alpha_{1}$ and ${\bar{Z}}_{i2}^{\prime }\alpha_{2}$ may be replaced by $B_{1}{\left(Z_{i1}\right)}^{\prime }\alpha_{1}$ and $B_{2}{\left(Z_{i2}\right)}^{\prime }\alpha_{2}$, where $B_{1}(\cdot )$ and $B_{2}(\cdot )$ are vectors of basis functions of the covariates that may include main effects, polynomial terms, and interactions among covariates. The balancing properties established above and the theoretical results in Sections 3 and 4 are unchanged under this case. The Stage I balancing property requires only that $D⊥Z_{1}|\{A=1,S_{1}(Z_{1};\alpha_{1})\}$, which holds when the conditional mean $E(D|A=1,Z_{1})$, together with an error distribution independent of $Z_{1}$, characterizes the conditional dose distribution; the Stage II results require correct specification of $P(A=1|Z_{2})$, which may likewise be achieved with a flexible linear predictor. Treatment–covariate interactions instead enter through the outcome model described above.

### 2.4 Regression Adjustment With a Two‐Part Propensity Score

Using the two‐part propensity score S(X;α) defined in Section 2.3, we consider regression adjustment based on (A,D,S):

(8) $$E\{Y|A,D,S(X;\alpha )\}=\eta_{0}+\eta_{11}A(D-c)+\eta_{12}A+S{\left(X;\alpha \right)}^{\prime }\eta_{2}$$

where $\eta ={\left(\eta_{0},\eta_{1}^{\prime },\eta_{2}^{\prime }\right)}^{\prime }$ with $\eta_{1}={\left(\eta_{11},\eta_{12}\right)}^{\prime }$ and $\eta_{2}={\left(\eta_{21},\eta_{22}\right)}^{\prime }$. Under Assumptions 1 to 3, correct specification of the exposure status model (7) gives the balancing property $A⊥Z_{2}|S_{2}(Z_{2};\alpha_{2})$, and correct specification of the dose model (6) with errors independent of $Z_{1}$ gives $D⊥Z_{1}|\{A=1,S_{1}(Z_{1};\alpha_{1})\}$ among exposed individuals; see Supporting Information A for the stage‐specific identification arguments. In particular, the dose‐response effect among exposed individuals is identified by adjustment for $S_{1}(Z_{1};\alpha_{1})$ in Stage I, whereas the reference‐dose effect is identified by adjustment for $S_{2}(Z_{2};\alpha_{2})$ after applying the Stage I offset in Stage II. Each stage therefore uses only the balancing property of its corresponding propensity‐score component.

In (8), the components of the two‐part propensity score enter the linear predictor linearly. More generally, $S_{1}(Z_{1};\alpha_{1})$ and $S_{2}(Z_{2};\alpha_{2})$ may enter through flexible functions, for example by replacing $S{\left(X;\alpha \right)}^{\prime }\eta_{2}$ with $h{\left\{S(X;\alpha )\right\}}^{\prime }\eta_{2}$, where h(·) contains polynomial or spline terms in each component of the propensity score [25]. Including higher‐order terms of the propensity score in the adjustment model can reduce residual variation when the relation between the outcome and the propensity score is nonlinear, and can thereby improve estimation and inference. The estimating equation framework and asymptotic results of Section 3 apply directly with these additional regressors, since they amount to an enlarged regression basis. For clarity of exposition we present the methods using the linear specification in (8).

## 3 A Framework for Two‐Stage Estimation

Regression adjustment hinges on the correct specification of both PS models, motivating us to develop a two‐stage formulation that separates estimation of the dose–response from the contrast between exposure and no exposure at a reference dose. In Stage I we estimate the causal dose–response effect among exposed individuals and in Stage II we estimate the causal effect of exposure at the reference dose compared to no exposure. We use PS regression adjustment in Stage I, and in Stage II use PS regression adjustment, inverse probability weighted estimating equations, or AIPW estimating equations, and derive the corresponding estimating equations, and present asymptotic properties.

### 3.1 Stage I: The Causal Dose‐Response Effect

Let n denote the sample size and index individuals by i=1,…,n. The observed data are ${𝒟}_{i}=(Y_{i},X_{i},A_{i},D_{i})$, i=1,…,n. Among exposed individuals ($A_{i}=1$), we consider a PS regression adjustment to assess the causal dose–response effect by fitting the model

(9) $$\begin{array}{cc} & E\left\{Y_{i}|A_{i}=1,D_{i},S_{1}(Z_{i1};\alpha_{1});\gamma_{1}\right\}\\ & =\gamma_{10}+\gamma_{11}D_{i}+\gamma_{12}S_{1}(Z_{i1};\alpha_{1})=\mu_{i11}(\phi_{1}), \end{array}$$

by least squares where $S_{1}(Z_{i1};\alpha_{1})=E(D_{i}|A_{i}=1,Z_{i1};\alpha_{1})$, $\gamma_{1}={\left(\gamma_{10},\gamma_{11},\gamma_{12}\right)}^{\prime }$, and $\phi_{1}={\left(\gamma_{1}^{\prime },\alpha_{1}^{\prime }\right)}^{\prime }$. The estimating equation for $\gamma_{1}$ is

(10) $$U_{11}(𝒟;\phi_{1})=\overset{n}{\underset{i=1}{\sum }}U_{i11}({𝒟}_{i};\phi_{1})=0,$$

where

$$U_{i11}({𝒟}_{i};\phi_{1})=A_{i}\frac{\partial \mu_{i11}(\phi_{1})}{\partial \gamma_{1}}\{Y_{i}-\mu_{i11}(\phi_{1})\}.$$

The parameter $\alpha_{1}$ can be estimated by solving

(11) $$U_{12}(𝒟;\alpha_{1})=\overset{n}{\underset{i=1}{\sum }}U_{i12}({𝒟}_{i};\alpha_{1})=0$$

where

$$U_{i12}({𝒟}_{i};\alpha_{1})=A_{i}\frac{\partial \mu_{i12}(\alpha_{1})}{\partial \alpha_{1}}\left\{D_{i}-\mu_{i12}(\alpha_{1})\right\}$$

with $\mu_{i12}(\alpha_{1})={\bar{Z}}_{i1}^{\prime }\alpha_{1}$. Considering (10) and (11) jointly, the Stage I estimating equation is

(12) $$U_{1}({𝒟}_{i};\phi_{1})=\overset{n}{\underset{i=1}{\sum }}U_{i1}({𝒟}_{i};\phi_{1})=0$$

with $U_{i1}({𝒟}_{i};\phi_{1})={\left(U_{i11}^{\prime }({𝒟}_{i};\phi_{1}),U_{i12}^{\prime }({𝒟}_{i};\alpha_{1})\right)}^{\prime }.$ Under the additive homogeneous structural outcome model (A.1) specified in Supporting Information A, Assumptions 1 to 3, and correct specification of the dose model (6) with errors independent of $Z_{1}$ (as in Theorem 1 of Section 3.3), the causal dose–response effect among exposed is represented by $\gamma_{11}$, which corresponds to $\psi_{11}$ in (3). This correspondence holds when the dose effect is constant; under effect heterogeneity, $\gamma_{11}$ instead targets a weighted average of individual dose–response effects over the exposed [25]. The enriched working model of Section 2.3 with treatment–covariate interactions allows the dose–response effect to vary across individuals, and recovers both the marginal and subgroup effects as shown in the heterogeneous treatment effect setting of Section 5.4.

### 3.2 Stage II: The Effect of Exposure at the Reference Dose

In Stage II, we assess the causal effect of exposure at a pre‐specified reference dose c compared to no exposure. This is achieved by conceptualizing $\gamma_{11}A_{i}(D_{i}-c)$ as an “offset”—in practice $\gamma_{11}$ will be estimated in Stage I and ${\hat{\gamma }}_{11}A_{i}(D_{i}-c)$ will be treated as a fixed quantity in Stage II analysis. Intuitively, the offset removes the portion of the outcome variation attributable to departures of the observed dose from the reference level among exposed individuals, thereby isolating the causal effect of exposure at dose c versus no exposure. The uncertainty in ${\hat{\gamma }}_{11}$ from Stage I is addressed in the large sample variance estimation. We consider three approaches to estimation at Stage II: (i) PS regression adjustment [25], (ii) inverse probability weighting [5], and (iii) augmented inverse probability weighting [17, 18].

#### 3.2.1 Propensity Score Regression Adjustment

To assess the effect of A=1 at dose D=c via a PS regression adjustment, we consider a Stage II linear predictor of the form

$$E\left\{Y_{i}|D_{i},A_{i},S_{2}(Z_{i2};\alpha_{2});\gamma_{2}\right\}=\mu_{i21}(\phi_{2})+\text{offset}\left\{\gamma_{11}A_{i}(D_{i}-c)\right\}$$

where $\mu_{i21}(\phi_{2})=\gamma_{20}+\gamma_{21}A_{i}+\gamma_{22}S_{2}(Z_{i2};\alpha_{2})$ with the Stage II PS $S_{2}(Z_{i2};\alpha_{2})=E(A_{i}|Z_{i2};\alpha_{2})$, and $\gamma_{2}={\left(\gamma_{20},\gamma_{21},\gamma_{22}\right)}^{\prime }$ with $\phi_{2}={\left(\gamma_{2}^{\prime },\alpha_{2}^{\prime }\right)}^{\prime }$. The regression parameter $\gamma_{2}$ can be estimated for specified ${\left(\gamma_{1}^{\prime },\alpha_{2}^{\prime }\right)}^{\prime }$ by the estimating equation

(13) $${\tilde{U}}_{21}(𝒟;\gamma_{1},\phi_{2})=\overset{n}{\underset{i=1}{\sum }}{\tilde{U}}_{i21}({𝒟}_{i};\gamma_{1},\phi_{2})=0$$

where

$$\begin{array}{cc} & {\tilde{U}}_{i21}({𝒟}_{i};\gamma_{1},\phi_{2})\\ & =\frac{\partial \mu_{i21}(\phi_{2})}{\partial \gamma_{2}}\left[Y_{i}-\left[\mu_{i21}(\phi_{2})+\text{offset}\left\{\gamma_{11}A_{i}(D_{i}-c)\right\}\right]\right]. \end{array}$$

The parameter $\alpha_{2}$ can be estimated by solving

(14) $$U_{22}\left(𝒟;\alpha_{2}\right)=\overset{n}{\underset{i=1}{\sum }}U_{i22}\left({𝒟}_{i};\alpha_{2}\right)=0$$

where

$$\begin{array}{cc} & U_{i22}\left({𝒟}_{i};\alpha_{2}\right)=\frac{\partial S_{2}(Z_{i2};\alpha_{2})}{\partial \alpha_{2}}\\ & \;\times \frac{1}{S_{2}(Z_{i2};\alpha_{2})\left\{1-S_{2}(Z_{i2};\alpha_{2})\right\}}\left\{A_{i}-S_{2}(Z_{i2};\alpha_{2})\right\}. \end{array}$$

Any binary regression model can be used but we employ a logistic link. To combine (13) and (14) we let ${\tilde{U}}_{i2}({𝒟}_{i};\gamma_{1},\phi_{2})={\left\{{\tilde{U}}_{i21}^{\prime }({𝒟}_{i};\gamma_{1},\phi_{2}),U_{i22}^{\prime }({𝒟}_{i};\alpha_{2})\right\}}^{\prime }$ and define

$${\tilde{U}}_{2}({𝒟}_{i};\gamma_{1},\phi_{2})=\overset{n}{\underset{i=1}{\sum }}{\tilde{U}}_{i2}({𝒟}_{i};\gamma_{1},\phi_{2})=0$$

as the joint Stage II estimating equations under PS regression adjustment. Taken together with the Stage I estimating equation, the full set of estimating equations for the two‐stage analysis under PS regression adjustment in Stage I and II is

(15) $$\tilde{U}(𝒟;\Omega )=\left(\begin{array}{l} U_{1}(𝒟;\phi_{1})\\ {\tilde{U}}_{2}(𝒟;\gamma_{1},\phi_{2}) \end{array}\right)=0$$

where $\Omega ={\left(\phi_{1}^{\prime },\phi_{2}^{\prime }\right)}^{\prime }$. Under the additive homogeneous structural outcome model (A.1) specified in Supporting Information A, Assumptions 1 to 3, and the correct specification of the PS model for the binary exposure status to obtain $S_{2}(Z_{i2};\alpha_{2})$, $\gamma_{21}$ is the causal effect of the exposure at reference dose compared to no exposure, and this holds likewise for the IPW and AIPW approaches described in Sections 3.2.2 and 3.2.3.

#### 3.2.2 Inverse Probability Weighting

We construct inverse probability weights based on $S_{2}(Z_{i2};\alpha_{2})$ to fit the Stage II MSM

$$E\left\{Y_{i}(a,d);\gamma_{1},\gamma_{2}\right\}=\gamma_{20}+\gamma_{21}a+\text{offset}\left\{\gamma_{11}a(d-c)\right\}.$$

An IPW estimating equation [5, 26] is

(16) $${\bar{U}}_{21}(𝒟;\gamma_{1},\phi_{2})=\overset{n}{\underset{i=1}{\sum }}{\bar{U}}_{i21}({𝒟}_{i};\gamma_{1},\phi_{2})=0$$

where

$$\begin{array}{cc} & {\bar{U}}_{i21}(𝒟;\gamma_{1},\phi_{2})=\overset{1}{\underset{a=0}{\sum }}w_{i}(a;\alpha_{2})\frac{\partial \mu_{21}(a;\gamma_{2})}{\partial \gamma_{2}}\\ & \;\times \left[Y_{i}-\left[\mu_{21}(a;\gamma_{2})+\text{offset}\left\{\gamma_{11}A_{i}(D_{i}-c)\right\}\right]\right] \end{array}$$

where $\mu_{21}(a;\gamma_{2})=\gamma_{20}+\gamma_{21}a$, and

(17) $$w_{i}(a;\alpha_{2})=\frac{I(A_{i}=a)}{S_{2}{\left(Z_{i2};\alpha_{2}\right)}^{a}{\left\{1-S_{2}(Z_{i2};\alpha_{2})\right\}}^{1-a}}$$

for a=0,1. The joint Stage II estimating equation under IPW is

$${\bar{U}}_{2}({𝒟}_{i};\gamma_{1},\phi_{2})=\overset{n}{\underset{i=1}{\sum }}{\bar{U}}_{i2}({𝒟}_{i};\gamma_{1},\phi_{2})=0$$

where ${\bar{U}}_{i2}({𝒟}_{i};\gamma_{1},\phi_{2})={\left({\bar{U}}_{i21}^{\prime }({𝒟}_{i};\gamma_{1},\phi_{2}),U_{i22}^{\prime }({𝒟}_{i};\alpha_{2})\right)}^{\prime }$. The two‐stage estimating equations with IPW are then

(18) $$\bar{U}(\Omega )=\left(\begin{array}{l} U_{1}(𝒟;\phi_{1})\\ {\bar{U}}_{2}(𝒟;\gamma_{1},\phi_{2}) \end{array}\right)=0.$$

We also implemented the normalized (Hájek) weights [27, 28], which divides $w_{i}(a;\alpha_{2})$ within each exposure arm by its empirical sum. Because the Stage II working model includes an intercept, the normalizing constants are absorbed into $\gamma_{20}$ and cancel from the reference‐dose contrast $\gamma_{21}$; the normalized and unnormalized estimators therefore coincide for our estimand, and the results are unchanged.

#### 3.2.3 Augmented Inverse Probability Weighting

An augmented inverse probability weighting (AIPW) estimating function [17] can yield consistent estimators of causal effects when at least one of the PS model and the imputation model is correctly specified [29]. In the current framework we consider the use of an AIPW approach in Stage II. When the Stage II propensity score model is correctly specified, we require that the exposure probability conditional on the full covariate vector is fully represented by $Z_{2}$ (i.e., $P(A=a|X)=P(A=a|Z_{2})$ for a=0,1); this condition is used in the proof of Theorem 2. We again estimate $\gamma_{2}$ via an augmented estimating equation

(19) $${\bar{\bar{U}}}_{21}(𝒟;\gamma_{1},\phi_{2})=\overset{n}{\underset{i=1}{\sum }}{\bar{\bar{U}}}_{i21}({𝒟}_{i};\gamma_{1},\phi_{2})=0,$$

and ${\bar{\bar{U}}}_{i21}({𝒟}_{i};\gamma_{1},\phi_{2})$ is given by

$$\begin{array}{cc} & \overset{1}{\underset{a=0}{\sum }}w_{i}(a;\alpha_{2})\frac{\partial \mu_{21}(a;\gamma_{2})}{\partial \gamma_{2}}\left[Y_{i}-\left[\mu_{21}(a;\gamma_{2})+\text{offset}\left\{\gamma_{11}A_{i}(D_{i}-c)\right\}\right]\right]\\ & \;-\{w_{i}(a;\alpha_{2})-1\}g_{i}(a;\theta ,\gamma_{2}) \end{array}$$

where $\phi_{2}={\left(\gamma_{2}^{\prime },\alpha_{2}^{\prime },\theta^{\prime }\right)}^{\prime }$ with $\theta ={\left(\theta_{1}^{\prime },\theta_{0}^{\prime }\right)}^{\prime }$, $w_{i}(a;\alpha_{2})$ is given in (17), and

$$g_{i}(a;\theta ,\gamma_{2})=\frac{\partial \mu_{21}(a;\gamma_{2})}{\partial \gamma_{2}}\left\{m_{ia}(\theta )-\mu_{21}(a;\gamma_{2})\right\}$$

where $m_{ia}(\theta )$ denotes the imputed model based expression for the expected response under treatment a∈{0,1}; from (4). The imputed values are specified as

$$\begin{array}{cc} m_{ia}(\theta_{a}) & =E\left\{Y_{i}-\gamma_{11}a(D_{i}-c)\;|A_{i}=a,X_{i};\theta_{a}\right\}\\ & =\theta_{a0}+\theta_{a1}^{\prime }X_{i1}+\theta_{a2}^{\prime }X_{i2}+\theta_{a3}^{\prime }X_{i3}, \end{array}$$

where $\theta_{ap}$ is $k_{p}$‐dimensional, p=1,2,3 for a=0,1. The estimating equation for θ with specified $\gamma_{1}$ is

(20) $$U_{23}(𝒟;\gamma_{1},\theta )=\overset{n}{\underset{i=1}{\sum }}U_{i23}({𝒟}_{i};\gamma_{1},\theta )=0$$

where $U_{i23}({𝒟}_{i};\gamma_{1},\theta )={\left\{U_{i231}^{\prime }({𝒟}_{i};\gamma_{1},\theta_{1}),U_{i230}^{\prime }({𝒟}_{i};\theta_{0})\right\}}^{\prime }$ with

$$U_{i23a}({𝒟}_{i};\gamma_{1},\theta_{a})=I(A_{i}=a)\;\frac{\partial m_{ia}(\theta_{a})}{\partial \theta_{a}}\left\{Y_{i}-\gamma_{11}a(D_{i}-c)-m_{ia}(\theta_{a})\right\}$$

for a=0,1. By combining (19), (14), and (20), let ${\bar{\bar{U}}}_{i2}({𝒟}_{i};\phi_{2})={\left\{{\bar{\bar{U}}}_{i21}^{\prime }({𝒟}_{i};\gamma_{1},\phi_{2}),U_{i22}^{\prime }({𝒟}_{i};\alpha_{2}),U_{i23}^{\prime }({𝒟}_{i};\gamma_{1},\theta )\right\}}^{\prime }$ and consider the joint Stage II AIPW estimating equation

(21) $${\bar{\bar{U}}}_{2}(𝒟;\gamma_{1},\phi_{2})=\overset{n}{\underset{i=1}{\sum }}{\bar{\bar{U}}}_{i2}({𝒟}_{i};\gamma_{1},\phi_{2})=0.$$

By combining (12) and (21), the joint estimating equation is

(22) $$\bar{\bar{U}}(\Omega )=\left(\begin{array}{l} U_{1}(𝒟;\phi_{1})\\ {\bar{\bar{U}}}_{2}(𝒟;\gamma_{1},\phi_{2}) \end{array}\right)=0.$$

### 3.3 Estimation and Statistical Inference

The two‐stage analysis can be characterized by

(23) $$U(𝒟;\Omega )=\left(\begin{array}{l} U_{1}(𝒟;\phi_{1})\\ U_{2}(𝒟;\gamma_{1},\phi_{2}) \end{array}\right)=0,$$

where $U_{1}(𝒟;\phi_{1})$ is defined in (12) and $U_{2}(𝒟;\gamma_{1},\phi_{2})$ denotes the Stage II estimating equations under regression adjustment, IPW, or AIPW, as given in (15), (18), and (22), respectively. From a theoretical perspective, treating (23) as a unified system is convenient for deriving the large sample properties of the resulting estimators, and the estimates $\hat{\Omega }={\left({\hat{\phi }}_{1}^{\prime },{\hat{\phi }}_{2}^{\prime }\right)}^{\prime }$ can be obtained by solving (23) directly. In practice, however, estimation proceeds sequentially: the Stage I estimating Equation (12) is solved to obtain ${\hat{\phi }}_{1}$ and hence ${\hat{\gamma }}_{11}$, which is then substituted into the Stage II estimating equation. Solving the latter yields ${\hat{\phi }}_{2}$ which includes ${\hat{\gamma }}_{21}$, the estimator of the causal effect of exposure at the reference dose.

We now establish the large sample properties of the two‐stage estimator with AIPW in Stage II; results for regression adjustment and IPW follow analogously. Let $\hat{\Omega }$ denote the solution to the joint estimating Equation (23), where $U_{i}(\cdot )$ is the stacked vector of estimating functions contributions from individual i from Stages I and II, i=1,…,n.

Theorem 1 *Suppose that the additive homogeneous structural outcome model* (*A*.1) *specified in* Supporting Information *A holds. If Assumptions *1 *to* 3 *hold and the PS model for* $S_{1}(Z_{1};\alpha_{1})$ *is correctly specified with error distribution independent of* $Z_{1}$, *so that* $D╨Z_{1}|\{A=1,\;S_{1}(Z_{1};\alpha_{1})\}$ *among exposed individuals, then the solution* ${\hat{\phi }}_{1}={\left({\hat{\gamma }}_{1}^{\prime },{\hat{\alpha }}_{1}^{\prime }\right)}^{\prime }$ *to* (12) *is consistent for* $\phi_{1}$. *In particular*, ${\hat{\gamma }}_{11}$ *is a consistent estimator of the causal dose–response effect among exposed* $\psi_{11}$ *in the MSM* (3).

Theorem 2 *Suppose that the additive homogeneous structural outcome model* (*A*.1) *specified in* Supporting Information *A holds. If Assumptions *1 *to* 3 *hold*, ${\hat{\gamma }}_{11}$ *is a consistent estimator of* $\psi_{11}$, *and at least one of the imputation model* $m_{a}(X;\theta )$ *or the PS model for* $S_{2}(Z_{2};\alpha_{2})$ *is correctly specified, where correct specification of the PS model means that*

$$P(A=a|X)=S_{2}{\left(Z_{2};\alpha_{2}\right)}^{a}{\left\{1-S_{2}(Z_{2};\alpha_{2})\right\}}^{1-a},\;a=0,1,$$

*then the solution* ${\hat{\gamma }}_{2}={\left({\hat{\gamma }}_{20},{\hat{\gamma }}_{21}\right)}^{\prime }$ *to the estimating Equation *(19) *is consistent for* $\gamma_{2}$. *In particular*, ${\hat{\gamma }}_{21}$ *is a consistent estimator of the causal effect of exposure at the reference dose compared to no exposure*, $\psi_{12}$, *in the MSM *(3).

Theorem 3 *Suppose that the additive homogeneous structural outcome model* (*A*.1) *specified in* Supporting Information *A holds. If Assumptions* 1 *to* 3 *hold, the model specification conditions required for consistency in Theorems* 1 *and* 2 *are satisfied, and standard regularity conditions* [30], *including uniqueness of the population solution and nonsingularity of* 𝒜(Ω), *hold, then the joint estimator* $\hat{\Omega }$ *is consistent for* Ω, *and* $\sqrt{n}\;(\hat{\Omega }-\Omega )$ *converges in distribution to a mean‐zero multivariate normal vector with covariance matrix*

$$\Sigma (\Omega )={𝒜}^{-1}(\Omega )\;ℬ(\Omega )\;{\left\{{𝒜}^{-1}(\Omega )\right\}}^{\prime },$$

*where*

$$𝒜(\Omega )=E\left\{-\frac{\partial U_{i}(\Omega )}{\partial \Omega^{\prime }}\right\}\;\text{and}\;ℬ(\Omega )=E\left\{U_{i}(\Omega )U_{i}^{\prime }(\Omega )\right\}.$$

Proofs of Theorems 1 to 3 and explicit expressions for the sandwich covariance estimator are given in Supporting Information B. We use the resulting sandwich covariance matrix for $\hat{\Omega }$ to compute standard errors, conduct Wald tests and construct confidence intervals.

## 4 Implication of Propensity Score Misspecification

The consistency of the causal estimators introduced above depends on the correct specification of the PS models used in the two‐stage procedure. The explicit form of the possibly biased estimator obtained by PS regression adjustment based on a possibly misspecified PS model for a semi‐continuous exposure under a one‐stage framework has been derived previously [15]. Here, we derive the limiting values of the estimators under misspecified PS models for the two‐stage approach introduced in Section 3, with PS regression adjustment applied in both stages. Under misspecification, these limiting values are not in general equal to the causal effects of interest.

### 4.1 Misspecification of the Stage I Propensity Score

We work with expectations of the estimating functions to characterize limiting values under misspecification [31]. Since the data are i.i.d., we suppress the subscript i and use (Y,A,D,X) to denote a generic observation. Let ${\tilde{S}}_{1}(X;{\tilde{\alpha }}_{1})$ denote the Stage I PS under misspecification parameterized by ${\tilde{\alpha }}_{1}$. A regression adjustment based on ${\tilde{S}}_{1}(X;{\tilde{\alpha }}_{1})$ involves fitting

$$\begin{array}{cc} & E(Y|A=1,D,{\tilde{S}}_{1}(X;{\tilde{\alpha }}_{1});{\tilde{\gamma }}_{1})\\ & ={\tilde{\gamma }}_{10}+{\tilde{\gamma }}_{11}D+{\tilde{\gamma }}_{12}{\tilde{S}}_{1}(X;{\tilde{\alpha }}_{1})=\mu_{11}({\tilde{\phi }}_{1}), \end{array}$$

where ${\tilde{\gamma }}_{1}={\left({\tilde{\gamma }}_{10},{\tilde{\gamma }}_{11},{\tilde{\gamma }}_{12}\right)}^{\prime }$ and ${\tilde{\phi }}_{1}={\left({\tilde{\gamma }}_{1}^{\prime },{\tilde{\alpha }}_{1}^{\prime }\right)}^{\prime }$. Let 𝒰(·) denote the corresponding estimating functions under misspecification. The limiting value of the estimator for $\gamma_{1}$ is the solution to

(24) $$E\left\{{𝒰}_{11}(𝒟;{\tilde{\phi }}_{1})\right\}=0,$$

with

$${𝒰}_{11}(𝒟;{\tilde{\phi }}_{1})=A\;\frac{\partial \mu_{11}({\tilde{\phi }}_{1})}{\partial {\tilde{\gamma }}_{1}}\{Y-\mu_{11}({\tilde{\phi }}_{1})\},$$

where the expectation is taken with respect to the true distribution of (Y,A,D,X). Solving (24) yields $\gamma_{1}^{*}={\left(\gamma_{10}^{*},\gamma_{11}^{*},\gamma_{12}^{*}\right)}^{\prime }$ with

(25) $$\begin{array}{l} \gamma_{11}^{*}=\theta_{11}+\frac{\theta_{2}^{\prime }\left[\zeta_{1}(X|A=1)-\beta_{1}(X|A=1)\rho_{1}\sqrt{\frac{\mathrm{var}\{E(D|X,A=1)\}}{\mathrm{var}\{{\tilde{S}}_{1}(X;{\tilde{\alpha }}_{1})\}}}\right]}{\mathrm{var}(D|A=1)-\mathrm{var}\{E(D|X,A=1)\}\rho_{1}^{2}}, \end{array}$$

where $\rho_{1}=\text{corr}\{E(D|X,A=1),{\tilde{S}}_{1}(X;{\tilde{\alpha }}_{1})\}$, 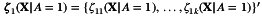, and 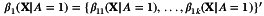, with $\zeta_{1j}(X|A=1)=\text{cov}\{E(D|X,A=1),X_{j}|A=1\}$ and $\beta_{1j}(X|A=1)=\text{cov}\{{\tilde{S}}_{1}(X;{\tilde{\alpha }}_{1}),X_{j}|A=1\}$ for j=1,…,k. Further details are provided in Supporting Information C.1.

When the PS model for the continuous exposure is correctly specified, ${\tilde{S}}_{1}(X;{\tilde{\alpha }}_{1})=E(D|X,A=1)$. In this case $\rho_{1}=1$, $\zeta_{1}(X|A=1)=\beta_{1}(X|A=1)$, and $\mathrm{var}\{E(D|X,A=1)\}=\mathrm{var}\{{\tilde{S}}_{1}(X;{\tilde{\alpha }}_{1})\}$. Substituting these equalities into (25) eliminates the second term, yielding $\gamma_{11}^{*}=\theta_{11}$. Otherwise, when ${\tilde{S}}_{1}(X;{\tilde{\alpha }}_{1})$ is misspecified, the asymptotic bias of ${\hat{\gamma }}_{11}$ depends on the covariance structure among E(D|X,A=1), X given A=1, and ${\tilde{S}}_{1}(X;{\tilde{\alpha }}_{1})$.

The bias term in (25) admits an omitted‐variable‐bias interpretation. Recall that $\gamma_{11}^{*}$ is the probability limit of the Stage I estimator ${\hat{\gamma }}_{11}$. By construction, ${\hat{\gamma }}_{11}$ is the coefficient on D in the regression of Y on $\left\{D,{\tilde{S}}_{1}(X;{\tilde{\alpha }}_{1})\right\}$ among exposed individuals, with the full confounder vector X omitted from the working model. This regression is equivalent, by the Frisch–Waugh–Lovell theorem [32, 33], to a two‐step procedure: first regress D on ${\tilde{S}}_{1}(X;{\tilde{\alpha }}_{1})$ among exposed individuals and let $\tilde{D}$ denote the resulting residual; then regress Y on $\tilde{D}$ (still among A=1). Since the omitted term in the working outcome model is $X^{\prime }\theta_{2}$, the linear projection of Y onto $\left(D,{\tilde{S}}_{1}\right)$ satisfies the classical omitted‐variable‐bias formula

$$\gamma_{11}^{*}=\theta_{11}+\frac{\text{cov}\left(X^{\prime }\theta_{2},\tilde{D}|A=1\right)}{\mathrm{var}\left(\tilde{D}|A=1\right)}.$$

In (25), the vector inside the brackets, multiplied by $\theta_{2}$, provides a componentwise representation of $\text{cov}(X^{\prime }\theta_{2},\tilde{D}|A=1)$: $\zeta_{1}(X|A=1)$ records the association between X and the true conditional mean dose E(D|X,A=1), while the correction $\beta_{1}(X|A=1)\;\rho_{1}\sqrt{\mathrm{var}\{E(D|X,A=1)\}/\mathrm{var}\{{\tilde{S}}_{1}(X;{\tilde{\alpha }}_{1})\}}$ subtracts the portion of this association that is already absorbed by ${\tilde{S}}_{1}(X;{\tilde{\alpha }}_{1})$. The denominator in (25) is the residual variance of D that remains after adjusting for ${\tilde{S}}_{1}(X;{\tilde{\alpha }}_{1})$. The Stage I bias is therefore small when $\rho_{1}$ is close to one, so that ${\tilde{S}}_{1}(X;{\tilde{\alpha }}_{1})$ captures most of the D–X association, or when $\theta_{2}$ is small in norm, so that any residual imbalance in X has limited impact on the outcome.

### 4.2 Biased Estimator in Stage II

We now derive the limiting value of the exposure effect at the reference dose compared to no exposure. Let ${\tilde{S}}_{2}(X;{\tilde{\alpha }}_{2})$ be the Stage II PS under misspecification, and then fit the regression model by treating ${\tilde{\gamma }}_{11}A(D-c)$ as an offset term in

$$\begin{array}{cc} & E\left\{Y|D,A,{\tilde{S}}_{2}(X;{\tilde{\alpha }}_{2});{\tilde{\gamma }}_{1},{\tilde{\gamma }}_{2}\right\}\\ & ={\tilde{\gamma }}_{20}+{\tilde{\gamma }}_{21}A+{\tilde{\gamma }}_{22}{\tilde{S}}_{2}(X;{\tilde{\alpha }}_{2})+\text{offset}\left\{{\tilde{\gamma }}_{11}A(D-c)\right\} \end{array}$$

where ${\tilde{\gamma }}_{2}={\left({\tilde{\gamma }}_{20},{\tilde{\gamma }}_{21},{\tilde{\gamma }}_{22}\right)}^{\prime }$. The estimating equation for ${\tilde{\gamma }}_{2}$ is

(26) $$E\left\{{𝒰}_{21}(𝒟;{\tilde{\gamma }}_{1},{\tilde{\phi }}_{2})\right\}=0$$

where

$${𝒰}_{21}(𝒟;{\tilde{\gamma }}_{1},{\tilde{\phi }}_{2})=\frac{\partial \mu_{21}({\tilde{\phi }}_{2})}{\partial {\tilde{\gamma }}_{2}}\left[Y-\left\{\mu_{21}({\tilde{\phi }}_{2})+{\tilde{\gamma }}_{11}A(D-c)\right\}\right]$$

with $\mu_{21}({\tilde{\phi }}_{2})={\tilde{\gamma }}_{20}+{\tilde{\gamma }}_{21}A+{\tilde{\gamma }}_{22}{\tilde{S}}_{2}(X;{\tilde{\alpha }}_{2})$ again; the expectation in (26) is taken with respect to the true distribution of (Y,D,A,X). Solving (26) with $\gamma_{11}^{*}$ given by (25) yields $\gamma_{2}^{*}={\left(\gamma_{20}^{*},\gamma_{21}^{*},\gamma_{22}^{*}\right)}^{\prime }$. Here we let $\rho_{21}=\text{corr}\left\{E(A|X),{\tilde{S}}_{2}(X;{\tilde{\alpha }}_{2})\right\}$, $\rho_{22}=\text{corr}\left\{E(AD|X),{\tilde{S}}_{2}(X;{\tilde{\alpha }}_{2})\right\}$, $\zeta_{2}(X)={\left\{\zeta_{21}(X),\ldots ,\zeta_{2k}(X)\right\}}^{\prime }$, $\beta_{2}(X)={\left\{\beta_{21}(X),\ldots ,\beta_{2k}(X)\right\}}^{\prime }$, and $\delta_{1}=\text{cov}\left\{E(A|X),{\tilde{S}}_{2}(X;{\tilde{\alpha }}_{2})\right\}$ where $\zeta_{2j}(X)=\text{cov}\left\{E(A|X),X_{j}\right\}$ and $\beta_{2j}(X)=\text{cov}\left\{{\tilde{S}}_{2}(X;{\tilde{\alpha }}_{2}),X_{j}\right\}$ for j=1,2,…,k. Then $\gamma_{21}^{*}$, the limiting value of the estimator for the reference‐dose exposure effect under misspecification, is

(27) $$\begin{array}{ll} & \gamma_{21}^{*}=\theta_{12}+\frac{\theta_{2}^{\prime }\left[\zeta_{2}(X)-\beta_{2}(X)\rho_{21}\sqrt{\frac{\mathrm{var}\left\{E(A|X)\right\}}{\mathrm{var}\{{\tilde{S}}_{2}(X;{\tilde{\alpha }}_{2})\}}}\right]}{\mathrm{var}(A)-\mathrm{var}\left\{E(A|X)\right\}\rho_{21}^{2}}\\ & \;+\frac{\begin{array}{c} (\theta_{11}-\gamma_{11}^{*})[\text{cov}(AD,A)-c\left[E\left\{\mathrm{var}(A|X)\right\}+(1-\rho_{21}^{2})\mathrm{var}\left\{E(A|X)\right\}\right]\\ \;-\;\rho_{21}\rho_{22}\sqrt{\mathrm{var}\left\{E(A|X)\right\}\mathrm{var}\left\{E(AD|X)\right\}}] \end{array}}{\mathrm{var}(A)-\mathrm{var}\left\{E(A|X)\right\}\rho_{21}^{2}}. \end{array}$$

Further details are provided in Supporting Information C.2.

Note that $\gamma_{21}^{*}=\theta_{12}$ when the second and third terms in (27) are zero. If ${\tilde{S}}_{2}(X;{\tilde{\alpha }}_{2})$ is correctly specified so that ${\tilde{S}}_{2}(X;{\tilde{\alpha }}_{2})=E(A|X)$, and the Stage I estimator is consistent (implying $\gamma_{11}^{*}=\theta_{11}$), these terms vanish and $\gamma_{21}^{*}=\theta_{12}$. More generally, the asymptotic bias in the Stage II estimator depends on the covariance and variance structure of E(A|X), X and ${\tilde{S}}_{2}(X;{\tilde{\alpha }}_{2})$, as well as any misspecification of ${\tilde{S}}_{1}(X;{\tilde{\alpha }}_{1})$ in Stage I. The bias decreases as ${\tilde{S}}_{2}(X;{\tilde{\alpha }}_{2})$ approaches E(A|X) and as $\gamma_{11}^{*}$ approaches $\theta_{11}$.

Equation (27) decomposes the limiting value $\gamma_{21}^{*}$ into the true reference‐dose effect $\theta_{12}$ and two interpretable bias components. The second term is the omitted‐variable‐bias contribution of $X^{\prime }\theta_{2}$ to the regression of Y on A after the part of A explained by ${\tilde{S}}_{2}(X;{\tilde{\alpha }}_{2})$ has been removed; it is structurally identical to the Stage I expression in (25), with D replaced by A and ${\tilde{S}}_{1}(X;{\tilde{\alpha }}_{1})$ replaced by ${\tilde{S}}_{2}(X;{\tilde{\alpha }}_{2})$, and represents residual confounding in the exposure status under Stage II propensity score misspecification. It vanishes when ${\tilde{S}}_{2}(X;{\tilde{\alpha }}_{2})=E(A|X)$. The third term, proportional to $\left(\theta_{11}-\gamma_{11}^{*}\right)$, represents the transmission of Stage I error into Stage II: after subtracting the offset ${\tilde{\gamma }}_{11}A(D-c)$, the pseudo‐outcome retains a leftover dose contribution $\left(\theta_{11}-\gamma_{11}^{*})A(D-c\right)$, which correlates with A through the joint dependence of (A,D) on X and is partially absorbed by ${\hat{\gamma }}_{21}$. The bracketed quantity $\text{cov}(AD,A)-c\left[E\{\mathrm{var}(A|X)\}+(1-\rho_{21}^{2})\mathrm{var}\{E(A|X)\}\right]-\rho_{21}\rho_{22}\sqrt{\mathrm{var}\{E(A|X)\}\mathrm{var}\{E(AD|X)\}}$ is the population regression coefficient that maps this leftover dose signal into bias in ${\hat{\gamma }}_{21}$. Thus, for $\gamma_{21}^{*}$ to equal $\theta_{12}$, both bias components must vanish. This occurs, in particular, when both propensity score models are correctly specified (so that ${\tilde{S}}_{2}(X;{\tilde{\alpha }}_{2})=E(A|X)$ and $\gamma_{11}^{*}=\theta_{11}$), but can also arise in special cases where the two bias terms cancel.

The limiting values $\gamma_{11}^{*}$ and $\gamma_{21}^{*}$ considered in this section should be interpreted as pseudo‐true parameter values under the selected working models. More specifically, they are the population roots of the corresponding estimating equations when one or more propensity score models are misspecified. For the propensity score regression adjustment procedures considered here, they may also be interpreted as population least‐squares projection coefficients. In general, these pseudo‐true parameter values need not equal the causal effects $\psi_{11}$ and $\psi_{12}$ and need not have a direct causal interpretation. Therefore, the expressions in this section are intended to characterize the forms and sources of asymptotic bias under misspecification, rather than to provide a robustness guarantee under misspecified models. Causal interpretation of the estimators relies on the identification assumptions and the relevant correct model specification conditions described in Sections 2 and 3.

## 5 Simulation Studies

### 5.1 Data Generation and Simulation Design

We consider two data generation processes.

#### 5.1.1 Data Generation Model 1

Let $X={\left(X_{1},\ldots ,X_{6}\right)}^{\prime }$ follow a multivariate normal distribution with mean zero, unit variances and pairwise correlation 0.2 with $X_{1}={\left(X_{11},X_{12}\right)}^{\prime }$, $X_{2}={\left(X_{21},X_{22}\right)}^{\prime }$, and $X_{3}={\left(X_{31},X_{32}\right)}^{\prime }$. The binary exposure $A|Z_{2}$ is generated from (7) with ${\bar{Z}}_{2}={\left(1,X_{2}^{\prime },X_{3}^{\prime }\right)}^{\prime }$, $\alpha_{21}={\left(\log1.2,\log2\right)}^{\prime }$ and $\alpha_{22}={\left(\log0.8,\log1.3\right)}^{\prime }$. We solve for $\alpha_{20}$ such that $P(A=1)=E_{Z_{2}}\left\{P(A=1|Z_{2})\right\}=0.25,0.5$ or 0.75 which give values $\alpha_{20}=-1.249$, 0.0008 and 1.249. For those exposed, the continuous exposure D is generated from (6) with ${\bar{Z}}_{1}={\left(1,X_{1}^{\prime },X_{3}^{\prime }\right)}^{\prime }$, $\alpha_{10}=0$, $\alpha_{11}={\left(1,0.5\right)}^{\prime }$, $\alpha_{12}={\left(0.6,0.8\right)}^{\prime }$ and a standard normal distributed error term. If $A_{i}=0$ for each individual $D_{i}$ undefined. Finally, we generate the response variable Y from model (4) with $\theta_{0}=0$, $\theta_{11}=0.5$, $\theta_{12}=4$, $\theta_{21}={\left(0.6,0.8\right)}^{\prime }$, $\theta_{22}={\left(0.8,0.4\right)}^{\prime }$, $\theta_{23}={\left(0.3,0.7\right)}^{\prime }$, and a standard normal distributed error term.

#### 5.1.2 Data Generation Model 2

Under this data generation process, we assume a common confounder vector X for both the exposure status and continuous exposure (i.e., $Z_{1}=Z_{2}=X$), and is therefore a simplified version of the data generation process described in Section 5.1.1. Let $X={\left(X_{1},X_{2},X_{3}\right)}^{\prime }$ follow a multivariate normal distribution with mean zero, unit variances and pairwise correlation 0.2. We generate A based on

(28) $$\log\left\{\frac{P(A=1|X)}{1-P(A=1|X)}\right\}=\alpha_{20}+\alpha_{21}^{\prime }X$$

with $\alpha_{21}={\left(\log1.2,\log2,-0.7\right)}^{\prime }$; we solve for $\alpha_{20}$ to give marginal probabilities of A=1 as 0.25,0.5 and 0.75, yielding $\alpha_{20}=-1.277$, 0.000571 and 1.278. If A=1, the continuous exposure D is generated from

(29) $$D=\alpha_{10}+\alpha_{11}^{\prime }X+W,$$

where $\alpha_{10}=0$, $\alpha_{11}={\left(1,0.5,0.5\right)}^{\prime }$, W⊥X, and W∼N(0,1). The second data generation model is the special case of (4)

(30) $$Y=\theta_{0}+\theta_{11}A(D-c)+\theta_{12}A+X^{\prime }\theta_{2}+E,$$

with $\theta_{0}=0$, $\theta_{11}=0.5$, $\theta_{12}=4$, $\theta_{2}={\left(0.8,0.8,0.4\right)}^{\prime }$, and E∼N(0,1).

### 5.2 Methods of Analysis

For the homogeneous simulations, the reference dose c is fixed at 0.396, 0.258, and 0.132 under Data Generation Model 1, and 0.138, 0.087, and 0.043 under Data Generation Model 2, for P(A=1)=0.25, 0.5, and 0.75, respectively. These values approximate the mean dose among exposed individuals and are held fixed across replications.

For each data generation model, we generate 2000 replicated datasets of size n=1000 and estimate the causal parameters using six methods: (i) a naive least‐squares fit of the MSM (3); (ii) the correctly specified response model; (iii) two–part PS regression adjustment in a single stage; (iv) two‐stage approach with PS regression in Stage II; (v) two‐stage approach with IPW in Stage II; and (vi) two‐stage approach with AIPW in Stage II.

Including covariates unrelated to the exposure but related to the outcome can reduce the variance of causal effect estimators without introducing bias [34]. Accordingly, we consider two propensity score specifications: (i) including only exposure‐related covariates and (ii) including all outcome‐related covariates. Under the second data generation model, these sets coincide, so the two specifications are identical.

To examine robustness, we consider five misspecification scenarios: (i) misspecified PS $S_{1}$ for the continuous exposure; (ii) misspecified PS $S_{2}$ for the binary exposure; (iii) misspecified imputation model $m_{a}$; (iv) misspecification of $S_{1}$ and $m_{a}$; and (v) misspecification of $S_{2}$ and $m_{a}$. In all cases we focus on the setting with minimal propensity scores $S={\left(S_{1}(Z_{1}),S_{2}(Z_{2})\right)}^{\prime }$. Full details of how each model is misspecified are given in Table 1.

**TABLE 1:** Model misspecification scenarios considered in the simulation study.

Scenario	Data Generation Model 1	Data Generation Model 2
(i) Misspecified $S_{1}$	omit $X_{12}$ from (6)	omit $X_{2}$ from (29)
(ii) Misspecified $S_{2}$	omit $X_{22}$ from (7)	omit $X_{2}$ from (28)
(iii) Misspecified $m_{a}$	omit $X_{22}$ from (4)	omit $X_{2}$ from (30)
(iv) Misspecified $S_{1}$ and $m_{a}$	omit $X_{12}$ from (6) + omit $X_{22}$ from (4)	omit $X_{2}$ from (29) + omit $X_{2}$ from (30)
(v) Misspecified $S_{2}$ and $m_{a}$	omit $X_{22}$ from (7) + omit $X_{22}$ from (4)	omit $X_{2}$ from (28) + omit $X_{2}$ from (30)

*Note:* $S_{1}$ represents the PS model for the continuous exposure D conditional on A=1, given by $S_{1}=E(D|A=1,Z_{1})$; $S_{2}$ represents the PS model for the binary exposure A, given by $S_{2}=E(A|Z_{2})$; $m_{a}$ represents the imputation models for a=0,1.

### 5.3 Finite Sample Performance

Table 2 presents the empirical bias (EBias), empirical standard error (ESE), average robust standard error (RSE), and empirical coverage probability (ECP) for estimators with P(A=1)=0.5 under correctly specified models. The naive method yields poor estimates for both parts across both data‐generation models. In contrast, all other methods show small biases and ECPs close to the nominal 95% level. Including all covariates in the PS models reduces empirical variance without inflating bias, in line with the variance reduction principle of [34]. The ESEs closely match the mean RSE, supporting the validity of the sandwich variance estimator in Section 3.3.

**TABLE 2:** Empirical results of estimators of causal effects with P(A=1)=0.5 under six methods of analysis based on correctly specified models.

Method	Exposure	Effect	Minimal PS: $S={\left\{S_{1}(Z_{1}),S_{2}(Z_{2})\right\}}^{\prime }$				Expanded PS: $S={\left\{S_{1}(X),S_{2}(X)\right\}}^{\prime }$			
			Ebias	ESE	RSE	ECP (%)	Ebias	ESE	RSE	ECP (%)
			*Data Generation Model 1*							
Naive	D	$\psi_{11}$	0.760	0.036	0.035	0.0	—	—	—	—
	A	$\psi_{12}$	0.952	0.128	0.126	0.0	—	—	—	—
DG model	D	$\psi_{11}$	−0.001	0.028	0.027	94.2	—	—	—	—
	A	$\psi_{12}$	<0.001	0.067	0.068	95.2	—	—	—	—
Two PS Reg	D	$\psi_{11}$	−0.002	0.034	0.034	94.2	−0.002	0.033	0.032	95.0
	A	$\psi_{12}$	−0.001	0.073	0.074	95.3	<0.001	0.067	0.068	95.0
${\text{PS}}_{I}+{\text{PS}}_{\text{II}}$	D	$\psi_{11}$	−0.002	0.060	0.059	94.3	−0.002	0.046	0.045	94.2
	A	$\psi_{12}$	−0.002	0.096	0.097	95.2	<0.001	0.068	0.069	95.5
${\text{PS}}_{I}+{\text{IPW}}_{\text{II}}$	D	$\psi_{11}$	−0.002	0.060	0.059	94.3	−0.002	0.046	0.045	94.2
	A	$\psi_{12}$	−0.002	0.107	0.107	94.5	<0.001	0.082	0.083	95.5
${\text{PS}}_{I}+{\text{AIPW}}_{\text{II}}$	D	$\psi_{11}$	−0.002	0.060	0.059	94.3	−0.002	0.046	0.045	94.2
	A	$\psi_{12}$	0.001	0.070	0.071	95.4	<0.001	0.069	0.070	95.2
			*Data Generation Model 2*							
Naive	D	$\psi_{11}$	0.637	0.035	0.034	0.0	—	—	—	—
	A	$\psi_{12}$	0.340	0.096	0.096	5.4	—	—	—	—
DG model	D	$\psi_{11}$	0.000	0.033	0.032	94.0	—	—	—	—
	A	$\psi_{12}$	−0.002	0.070	0.068	94.3	—	—	—	—
Two PS Reg	D	$\psi_{11}$	0.000	0.033	0.032	94.3	—	—	—	—
	A	$\psi_{12}$	−0.002	0.070	0.069	94.3	—	—	—	—
${\text{PS}}_{I}+{\text{PS}}_{\text{II}}$	D	$\psi_{11}$	−0.001	0.045	0.045	95.1	—	—	—	—
	A	$\psi_{12}$	−0.002	0.070	0.069	94.0	—	—	—	—
${\text{PS}}_{I}+{\text{IPW}}_{\text{II}}$	D	$\psi_{11}$	−0.001	0.045	0.045	95.1	—	—	—	—
	A	$\psi_{12}$	−0.001	0.078	0.076	94.2	—	—	—	—
${\text{PS}}_{I}+{\text{AIPW}}_{\text{II}}$	D	$\psi_{11}$	−0.001	0.045	0.045	95.1	—	—	—	—
	A	$\psi_{12}$	−0.002	0.072	0.070	94.5	—	—	—	—

*Note:* DG model: Data generation model; Two PS Reg: Two‐part PS regression adjustment; ${\text{PS}}_{I}+{\text{PS}}_{\text{II}}$: Using PS regression adjustment in Stage I and II; ${\text{PS}}_{I}+{\text{IPW}}_{\text{II}}$: Using PS regression adjustment in Stage I and IPW in Stage II; ${\text{PS}}_{I}+{\text{AIPW}}_{\text{II}}$: Using PS regression adjustment in Stage I and AIPW in Stage II.

Table 3 reports results under misspecified $S_{1}$ and $S_{2}$ with P(A=1)=0.5. Misspecification of $S_{1}$ induces dose‐effect bias and can also affect the status estimate, while misspecification of $S_{2}$ primarily affects the status estimate.

**TABLE 3:** Empirical results for estimators of causal effects with P(A=1)=0.5 for two‐part PS regression adjustment and two‐stage analysis under model misspecification.

Method	Exposure	Effect	Misspecified $S_{1}$				Misspecified $S_{2}$			
			Ebias	ESE	RSE	ECP (%)	Ebias	ESE	RSE	ECP (%)
			*Data Generation Model 1*							
Two PS Reg	D	$\psi_{11}$	0.135	0.039	0.038	6.0	−0.002	0.035	0.034	94.3
	A	$\psi_{12}$	0.034	0.084	0.086	93.5	0.245	0.074	0.075	9.7
${\text{PS}}_{I}$+${\text{PS}}_{\text{II}}$	D	$\psi_{11}$	0.348	0.062	0.062	0.1	−0.002	0.060	0.059	94.3
	A	$\psi_{12}$	0.089	0.093	0.094	84.0	0.331	0.095	0.099	7.5
${\text{PS}}_{I}$+${\text{IPW}}_{\text{II}}$	D	$\psi_{11}$	0.348	0.062	0.062	0.1	−0.002	0.060	0.059	94.3
	A	$\psi_{12}$	0.089	0.103	0.102	84.3	0.330	0.097	0.100	7.8
			*Data Generation Model 2*							
Two PS Reg	D	$\psi_{11}$	0.074	0.037	0.037	47.1	−0.001	0.033	0.032	94.7
	A	$\psi_{12}$	0.005	0.070	0.069	93.8	0.188	0.075	0.074	27.7
${\text{PS}}_{I}$+${\text{PS}}_{\text{II}}$	D	$\psi_{11}$	0.280	0.049	0.049	0.0	−0.001	0.045	0.045	95.1
	A	$\psi_{12}$	0.023	0.072	0.071	93.3	0.469	0.082	0.081	0.0
${\text{PS}}_{I}$+${\text{IPW}}_{\text{II}}$	D	$\psi_{11}$	0.280	0.049	0.049	0.0	−0.001	0.045	0.045	95.1
	A	$\psi_{12}$	0.023	0.079	0.077	92.9	0.469	0.084	0.082	0.0

*Note:* $S_{1}$ represents the PS model for the continuous exposure D conditioned on A=1, given by $S_{1}=E(D|A=1,Z_{1})$; $S_{2}$ represents the PS model for the binary exposure A, given by $S_{2}=E(A|Z_{2})$.

Table 4 summarizes the performance of the Stage II AIPW estimators under model misspecification with P(A=1)=0.5. When either the imputation model or the $S_{2}$ model is misspecified, the estimator remains consistent. In contrast, misspecified $S_{1}$ induces bias in both stages, and simultaneously misspecifying the imputation and $S_{2}$ models leads to biased Stage II estimates and loss of nominal coverage.

**TABLE 4:** Empirical results for estimators of effects for both parts with P(A=1)=0.5 for applying PS regression adjustment in Stage I and AIPW in Stage II under model misspecification.

Misspecified model	Exposure	Effect	Ebias	ESE	RSE	ECP (%)
			*Data Generation Model 1*			
$S_{1}$	D	$\psi_{11}$	0.348	0.062	0.062	0.1
	A	$\psi_{12}$	0.091	0.077	0.076	78.6
$S_{2}$	D	$\psi_{11}$	−0.002	0.060	0.059	94.3
	A	$\psi_{12}$	0.000	0.068	0.070	95.4
$m_{a}(X;\theta )$	D	$\psi_{11}$	−0.002	0.060	0.059	94.3
	A	$\psi_{12}$	0.002	0.071	0.072	95.3
$S_{1}$ + $m_{a}(X;\theta )$	D	$\psi_{11}$	0.348	0.062	0.062	0.1
	A	$\psi_{12}$	0.093	0.078	0.077	77.7
$S_{2}$ + $m_{a}(X;\theta )$	D	$\psi_{11}$	−0.002	0.060	0.059	94.3
	A	$\psi_{12}$	0.225	0.069	0.071	10.6
			*Data Generation Model 2*			
$S_{1}$	D	$\psi_{11}$	0.280	0.049	0.049	0.0
	A	$\psi_{12}$	0.023	0.074	0.073	93.5
$S_{2}$	D	$\psi_{11}$	−0.001	0.045	0.045	95.1
	A	$\psi_{12}$	−0.002	0.070	0.069	94.4
$m_{a}(X;\theta )$	D	$\psi_{11}$	−0.001	0.045	0.045	95.1
	A	$\psi_{12}$	<0.001	0.077	0.074	94.5
$S_{1}$ +$m_{a}(X;\theta )$	D	$\psi_{11}$	0.280	0.049	0.049	0.0
	A	$\psi_{12}$	0.025	0.078	0.076	93.2
$S_{2}$ + $m_{a}(X;\theta )$	D	$\psi_{11}$	−0.001	0.045	0.045	95.1
	A	$\psi_{12}$	0.470	0.083	0.082	0.0

*Note:* $S_{1}$ represents the PS model for the continuous exposure D conditioned on A=1, given by $S_{1}=E(D|A=1,Z_{1})$; $S_{2}$ represents the PS model for the binary exposure A, given by $S_{2}=E(A|Z_{2})$; $m_{a}(X;\theta )$ represents the imputation models for a=0,1.

Tables S1–S3 and S4–S6 in Supporting Information D present results for P(A=1)=0.25 and 0.75 respectively, and show patterns consistent with those for P(A=1)=0.5.

### 5.4 Simulation Design Under Heterogeneous Treatment Effects

To assess the performance of the proposed estimators when the constant treatment effect assumption is violated, we extend Data Generation Model 1 by introducing effect modification through a binary modifier derived from a common confounder. Let $X={\left(X_{11},X_{12},X_{21},X_{22},X_{31},X_{32}\right)}^{\prime }$ follow a multivariate normal distribution with mean zero, unit variances, and pairwise correlation 0.2, and define $X_{1}={\left(X_{11},X_{12}\right)}^{\prime }$, $X_{2}={\left(X_{21},X_{22}\right)}^{\prime }$, $X_{3}={\left(X_{31},X_{32}\right)}^{\prime }$, $Z_{1}={\left(X_{1}^{\prime },X_{3}^{\prime }\right)}^{\prime }$, and $Z_{2}={\left(X_{2}^{\prime },X_{3}^{\prime }\right)}^{\prime }$ as in Section 5.1.1. The binary modifier is defined as $V=I(X_{31}>0)$, derived from the common confounder $X_{31}\in X_{3}$, so that V enters both $Z_{1}$ and $Z_{2}$ through its parent $X_{31}$: it confounds exposure status and dose, and simultaneously modifies the causal effects. The binary exposure $A|Z_{2}$ is generated from (7) with $\alpha_{21}={\left(\log1.2,\log2\right)}^{\prime }$ and $\alpha_{22}={\left(\log0.8,\log1.3\right)}^{\prime }$, with $\alpha_{20}$ chosen so that P(A=1)=0.5. For exposed individuals, the continuous dose is generated from (6) with $\alpha_{10}=0$, $\alpha_{11}={\left(1,0.5\right)}^{\prime }$, $\alpha_{12}={\left(0.6,0.8\right)}^{\prime }$ and a standard normal error term; D is undefined when A=0. The response is generated from the heterogeneous structural model

(31) $$\begin{array}{cc} Y & =\theta_{0}+\theta_{11}A(D-c)+\theta_{12}A+\theta_{13}A(D-c)V+\theta_{14}AV\\ & \;+X^{\prime }\theta_{2}+\varepsilon_{Y},\;\varepsilon_{Y}∼N(0,1), \end{array}$$

with $\theta_{0}=0$, $\theta_{11}=0.5$, $\theta_{12}=4$, $\theta_{21}={\left(0.6,0.8\right)}^{\prime }$, $\theta_{22}={\left(0.8,0.4\right)}^{\prime }$, $\theta_{23}={\left(0.3,0.7\right)}^{\prime }$, and reference dose c set to the mean of D among exposed individuals in an independent Monte Carlo sample of 200,000 individuals, yielding c=0.248. This reference dose is held fixed across simulation replications. The interaction coefficients $\left(\theta_{13},\theta_{14}\right)$ control the strength of heterogeneity, and we consider three settings: no heterogeneity (HG‐0: $\theta_{13}=0$, $\theta_{14}=0$), mild heterogeneity (HG‐mild: $\theta_{13}=2$, $\theta_{14}=0.25$), and strong heterogeneity (HG‐strong: $\theta_{13}=4$, $\theta_{14}=0.5$).

Under (31), the marginal dose–response effect among exposed individuals targeted by $\psi_{11}$ in the MSM is $\psi_{11}^{*}=\theta_{11}+\theta_{13}P(V=1|A=1)$, and the marginal reference‐dose effect targeted by $\psi_{12}$ is $\psi_{12}^{*}=\theta_{12}+\theta_{14}P(V=1)$; the corresponding subgroup‐specific effects are $\psi_{11}^{v}=\theta_{11}+\theta_{13}v$ and $\psi_{12}^{v}=\theta_{12}+\theta_{14}v$ for v=0,1. Since $V=I(X_{31}>0)$ with $X_{31}∼N(0,1)$, P(V=1)=0.5 exactly. Then the marginal‐effect benchmarks use the Monte Carlo averages of the estimated modifier proportions over the 2000 replications: P(V=1|A=1)≈0.501 and P(V=1)≈0.499. The resulting marginal dose‐effect benchmarks are 0.500, 1.501, and 2.502, and the status‐effect benchmarks are 4.000, 4.125, and 4.250 under HG‐0, HG‐mild, and HG‐strong, respectively. The subgroup effects are $\psi_{11}^{0}=0.5$ and $\psi_{12}^{0}=4$ in all scenarios, with $\psi_{11}^{1}=0.5$, 2.5, and 4.5 and $\psi_{12}^{1}=4$, 4.25, and 4.5 across the three scenarios. For each scenario we generate 2000 replicated datasets of size n=1000 and apply the six methods of Section 5.2, with each method based on the enriched working model of Section 2.3 including the A(D−c)V and AV interaction terms; the marginal effects are estimated on the full sample and the subgroup effects are recovered from the same fit via the appropriate linear combinations of the estimated coefficients. The AIPW outcome‐imputation regressions include linear terms in the six baseline covariates. The Stage II propensity score is correctly specified in these heterogeneous‐effect simulations.

Tables 5 and 6 report the empirical results. For the marginal effects in Table 5, the naive method is severely biased in every scenario, while all other methods show negligible bias and empirical coverage close to the nominal 95% level across all three heterogeneity settings. The precision of the marginal dose–response estimate decreases as the strength of heterogeneity increases, reflecting the additional variability induced by the interaction terms. For the subgroup‐specific effects in Table 6, the naive method is again severely biased for all targets, whereas the remaining methods recover both the subgroup‐specific dose–response and reference‐dose effects with negligible bias and near‐nominal coverage. These results confirm that, when the working model accommodates the relevant interactions, the proposed two‐stage estimators recover both the marginal and subgroup‐specific dose–response and reference‐dose effects under effect heterogeneity. The empirical standard errors closely match the average robust standard errors, supporting the validity of the sandwich variance estimator under the enriched specification. As in the homogeneous setting, the weighting‐based Stage II estimators are less efficient than the regression‐based approaches for the reference‐dose effect, with the AIPW estimator recovering much of this efficiency loss.

**TABLE 5:** Empirical results of estimators of marginal causal effects with P(A=1)=0.5 under six methods of analysis for the heterogeneous treatment effects setting.

Method	Exp.	Effect	HG‐0: $\theta_{13}=0,\theta_{14}=0$				HG‐mild: $\theta_{13}=2,\theta_{14}=0.25$				HG‐strong: $\theta_{13}=4,\theta_{14}=0.5$			
			Ebias	ESE	RSE	ECP (%)	Ebias	ESE	RSE	ECP (%)	Ebias	ESE	RSE	ECP (%)
Naive	D	$\psi_{11}^{*}$	0.731	0.039	0.038	0.0	0.731	0.059	0.059	0.0	0.731	0.096	0.097	0.0
	A	$\psi_{12}^{*}$	0.945	0.133	0.129	0.0	0.945	0.132	0.129	0.0	0.945	0.131	0.128	0.0
DG model	D	$\psi_{11}^{*}$	−0.002	0.029	0.028	94.5	−0.002	0.053	0.053	94.8	−0.002	0.093	0.094	94.8
	A	$\psi_{12}^{*}$	<0.001	0.070	0.071	94.8	<0.001	0.070	0.071	94.7	<0.001	0.070	0.071	94.8
Two PS Reg	D	$\psi_{11}^{*}$	−0.002	0.037	0.036	95.2	−0.002	0.057	0.057	95.1	−0.002	0.095	0.096	95.2
	A	$\psi_{12}^{*}$	−0.001	0.077	0.078	94.8	−0.001	0.077	0.078	94.7	−0.001	0.078	0.078	94.7
${\text{PS}}_{I}+{\text{PS}}_{\text{II}}$	D	$\psi_{11}^{*}$	−0.002	0.060	0.059	94.1	−0.002	0.075	0.074	94.3	−0.002	0.108	0.107	95.0
	A	$\psi_{12}^{*}$	−0.001	0.105	0.106	95.3	−0.001	0.105	0.106	95.2	−0.001	0.106	0.106	95.2
${\text{PS}}_{I}+{\text{IPW}}_{\text{II}}$	D	$\psi_{11}^{*}$	−0.002	0.060	0.059	94.1	−0.002	0.075	0.074	94.3	−0.002	0.108	0.107	95.0
	A	$\psi_{12}^{*}$	0.001	0.115	0.114	94.8	0.001	0.115	0.114	94.9	0.001	0.115	0.114	95.0
${\text{PS}}_{I}+{\text{AIPW}}_{\text{II}}$	D	$\psi_{11}^{*}$	−0.002	0.060	0.059	94.1	−0.002	0.075	0.074	94.3	−0.002	0.108	0.107	95.0
	A	$\psi_{12}^{*}$	0.001	0.076	0.076	95.2	0.001	0.076	0.076	95.5	0.001	0.076	0.077	95.5

*Note:* $\psi_{11}^{*}$ and $\psi_{12}^{*}$ denote the marginal dose‐response effect among exposed and the marginal reference‐dose effect, respectively. DG model: Data generation model; Two PS Reg: Two‐part PS regression adjustment; ${\text{PS}}_{I}+{\text{PS}}_{\text{II}}$: Using PS regression adjustment in Stage I and II; ${\text{PS}}_{I}+{\text{IPW}}_{\text{II}}$: Using PS regression adjustment in Stage I and IPW in Stage II; ${\text{PS}}_{I}+{\text{AIPW}}_{\text{II}}$: Using PS regression adjustment in Stage I and AIPW in Stage II.

**TABLE 6:** Empirical results of estimators of subgroup‐specific causal effects with P(A=1)=0.5 under six methods of analysis for the heterogeneous treatment effects setting.

Method	Exp.	Effect	HG‐0: $\theta_{13}=0,\theta_{14}=0$				HG‐mild: $\theta_{13}=2,\theta_{14}=0.25$				HG‐strong: $\theta_{13}=4,\theta_{14}=0.5$			
			Ebias	ESE	RSE	ECP (%)	Ebias	ESE	RSE	ECP (%)	Ebias	ESE	RSE	ECP (%)
Naive	D	$\psi_{11}^{0}$	0.732	0.055	0.054	0.0	0.732	0.055	0.054	0.0	0.732	0.055	0.054	0.0
	D	$\psi_{11}^{1}$	0.731	0.054	0.054	0.0	0.731	0.054	0.054	0.0	0.731	0.054	0.054	0.0
	A	$\psi_{12}^{0}$	0.795	0.159	0.153	0.2	0.795	0.159	0.153	0.2	0.795	0.159	0.153	0.2
	A	$\psi_{12}^{1}$	1.095	0.154	0.153	0.0	1.095	0.154	0.153	0.0	1.095	0.154	0.153	0.0
DG model	D	$\psi_{11}^{0}$	−0.002	0.038	0.037	94.3	−0.002	0.038	0.037	94.3	−0.002	0.038	0.037	94.3
	D	$\psi_{11}^{1}$	−0.001	0.037	0.037	94.1	−0.001	0.037	0.037	94.1	−0.001	0.037	0.037	94.1
	A	$\psi_{12}^{0}$	−0.003	0.092	0.092	94.8	−0.003	0.092	0.092	94.8	−0.003	0.092	0.092	94.8
	A	$\psi_{12}^{1}$	0.003	0.087	0.089	95.8	0.003	0.087	0.089	95.8	0.003	0.087	0.089	95.8
Two PS Reg	D	$\psi_{11}^{0}$	−0.002	0.048	0.046	94.2	−0.002	0.048	0.046	94.2	−0.002	0.048	0.046	94.2
	D	$\psi_{11}^{1}$	−0.002	0.047	0.046	95.0	−0.002	0.047	0.046	95.0	−0.002	0.047	0.046	95.0
	A	$\psi_{12}^{0}$	−0.002	0.119	0.118	94.4	−0.002	0.119	0.118	94.4	−0.002	0.119	0.118	94.4
	A	$\psi_{12}^{1}$	0.001	0.112	0.113	94.8	0.001	0.112	0.113	94.8	0.001	0.112	0.113	94.8
${\text{PS}}_{I}+{\text{PS}}_{\text{II}}$	D	$\psi_{11}^{0}$	−0.002	0.069	0.067	94.8	−0.002	0.069	0.067	94.8	−0.002	0.069	0.067	94.8
	D	$\psi_{11}^{1}$	−0.002	0.068	0.067	94.5	−0.002	0.068	0.067	94.5	−0.002	0.068	0.067	94.5
	A	$\psi_{12}^{0}$	−0.004	0.170	0.168	94.8	−0.004	0.170	0.168	94.8	−0.004	0.170	0.168	94.8
	A	$\psi_{12}^{1}$	0.001	0.159	0.159	94.7	0.001	0.159	0.159	94.7	0.001	0.159	0.159	94.7
${\text{PS}}_{I}+{\text{IPW}}_{\text{II}}$	D	$\psi_{11}^{0}$	−0.002	0.069	0.067	94.8	−0.002	0.069	0.067	94.8	−0.002	0.069	0.067	94.8
	D	$\psi_{11}^{1}$	−0.002	0.068	0.067	94.5	−0.002	0.068	0.067	94.5	−0.002	0.068	0.067	94.5
	A	$\psi_{12}^{0}$	−0.003	0.211	0.206	94.2	−0.003	0.211	0.206	94.2	−0.003	0.211	0.206	94.2
	A	$\psi_{12}^{1}$	0.005	0.204	0.202	94.2	0.005	0.204	0.202	94.2	0.005	0.204	0.202	94.2
${\text{PS}}_{I}+{\text{AIPW}}_{\text{II}}$	D	$\psi_{11}^{0}$	−0.002	0.069	0.067	94.8	−0.002	0.069	0.067	94.8	−0.002	0.069	0.067	94.8
	D	$\psi_{11}^{1}$	−0.002	0.068	0.067	94.5	−0.002	0.068	0.067	94.5	−0.002	0.068	0.067	94.5
	A	$\psi_{12}^{0}$	−0.002	0.123	0.121	94.2	−0.002	0.123	0.121	94.1	−0.001	0.123	0.121	94.0
	A	$\psi_{12}^{1}$	0.004	0.106	0.106	95.1	0.004	0.106	0.106	95.1	0.004	0.106	0.107	95.4

*Note:* $\psi_{11}^{v}$ and $\psi_{12}^{v}$ denote the subgroup‐specific dose‐response effect among exposed and the subgroup‐specific reference‐dose effect, respectively, in subgroup V=v for v=0,1. DG model: Data generation model; Two PS Reg: Two‐part PS regression adjustment; ${\text{PS}}_{I}+{\text{PS}}_{\text{II}}$: Using PS regression adjustment in Stage I and II; ${\text{PS}}_{I}+{\text{IPW}}_{\text{II}}$: Using PS regression adjustment in Stage I and IPW in Stage II; ${\text{PS}}_{I}+{\text{AIPW}}_{\text{II}}$: Using PS regression adjustment in Stage I and AIPW in Stage II.

## 6 Application to the Prenatal Alcohol Exposure Study

### 6.1 Description of the Detroit Longitudinal Cohort

The Detroit Longitudinal Cohort is a prospective study of 480 pregnant African‐American women from inner‐city Detroit and their children, followed from birth to age 19 to investigate the effects of PAE [11, 19]. Prenatal alcohol intake was assessed at each visit using a timeline follow‐back interview [19] and summarized as average daily consumption over pregnancy; let T denote the average ounces of AA per day. We treat PAE as a semi‐continuous exposure: A=I(T>0) is a binary indicator of any drinking (16.2% of mothers reported no drinking during pregnancy), and D=logT is the log daily dose among drinkers. The reference dose is set to c=−2.31, the sample mean of D among exposed mothers. With this choice, the parameter $\psi_{12}$ in the MSM represents the average causal effect of any prenatal drinking, when drinkers consume their typical dose, compared to complete abstention. Alternative choices of c, such as a clinically defined low‐risk threshold, would yield different but related estimands $\Delta_{0}(c)$; within our linear MSM, estimates at such alternative reference levels are simple linear transformations of the estimated pair $\left({\hat{\psi }}_{11},{\hat{\psi }}_{12}\right)$, as described in Section 2. The log transformation yields an approximately symmetric dose distribution among drinkers (Figure 1B), supporting the normality assumption of the dose model (6) used in Stage I.

The outcome is the Full Scale IQ score of the Wechsler Intelligence Scales for Children, 3rd edition (WISC‐III) [20], measured at age 7 years for 377 children. Among children with observed outcomes, the mean (SD) Full Scale IQ score was 84.22 (12.31).

We adjust for baseline maternal sociodemographic characteristics, smoking and other substance use during pregnancy, reproductive history, gestational age at screening, and measures of the home environment and maternal cognition (listed in Table S7 in Supporting Information E.1). To address missingness, we generate 20 multiply imputed datasets using an imputation model including all variables; further details are given in Supporting Information E.2. Point estimates and standard errors are combined using Rubin's rules under a missing‐at‐random assumption [35, 36].

### 6.2 Application and Findings

We estimate the causal effects of prenatal drinking status and continuous dose using five approaches: (i) conventional covariate regression adjustment; (ii) two–part PS regression adjustment; (iii) a two‐stage analysis with PS regression adjustment in Stage II; (iv) a two‐stage analysis with IPW in Stage II; and (v) a two‐stage analysis with AIPW in Stage II. Implementation details are provided in Supporting Information E.3.

Supporting Information E.1 summarizes the fitted models for the continuous and binary exposure components, E(D|A=1,X) and P(A=1|X). Both models include all candidate covariates to improve precision of causal effect estimates [34].

Table 7 reports the estimated causal effects under the five approaches. For all PS–based methods, the estimated effects of both log(AA/day) among the exposed and drinking status are negative, with the magnitude of the drinking status effect larger than that of the dose effect. By contrast, the conventional covariate adjustment model yields a small positive estimate (0.253) for drinking status and a slightly negative estimate (−0.125) for log dose. Given the reliance of covariate adjustment on correct specification of the outcome model [34, 37], these estimates may be more vulnerable to bias than those from the PS–based procedures.

**TABLE 7:** Estimated effects of drinking status and log prenatal AA consumption per day.

	D|A=1		A	
Method	Estimate	Standard error	Estimate	Standard error
Covariate adjustment	−0.125	0.515	0.253	1.655
Two–PS regression	−0.189	0.572	−0.128	1.621
${\text{PS}}_{I}+{\text{PS}}_{\text{II}}$	−0.011	0.518	−0.013	1.634
${\text{PS}}_{I}+{\text{IPW}}_{\text{II}}$	—	—	−0.268	2.128
${\text{PS}}_{I}+{\text{AIPW}}_{\text{II}}$	—	—	−0.176	1.965

*Note:* Two–PS regression: two‐dimensional PS regression adjustment; ${\text{PS}}_{I}+{\text{PS}}_{\text{II}}$: PS regression adjustment in Stages I and II; ${\text{PS}}_{I}+{\text{IPW}}_{\text{II}}$: PS regression in Stage I and IPW in Stage II; ${\text{PS}}_{I}+{\text{AIPW}}_{\text{II}}$: PS regression in Stage I and AIPW in Stage II.

For the log‐dose effect among drinkers, PS–based point estimates are small and similar across methods, with standard errors around 0.5. For the drinking status effect, the PS–based estimates range from approximately −0.013 to −0.268, whereas the conventional covariate adjustment estimate is slightly positive. The two‐stage AIPW estimator provides a negative point estimate (−0.176) and remains consistent if either the Stage II propensity score or the imputation model is correctly specified, provided the Stage I dose–response effect is consistently estimated, but, as in our simulations, weighting‐based methods yield larger standard errors than regression‐based approaches (approximately 2.0 versus 1.6). Taken together, the PS–based results are compatible with modest harmful causal effects of both increased prenatal alcohol dose among drinkers and drinking status at the reference dose on WISC‐III full scale IQ, although none of the estimated effects is statistically significant at the 5% level.

### 6.3 Assessing Effect Modification by Maternal Age

Prior analyses of the Detroit cohort indicate that the adverse effect of PAE on child cognition is concentrated among the offspring of older mothers: alcohol‐related deficits were found most strongly in the children of women over 30 years of age [38], and an effect of PAE on WISC‐III Full Scale IQ similarly emerged among children born to older mothers [11]. Motivated by these findings, we illustrate the heterogeneous specification of Section 5.4 by allowing the causal effects to vary with a binary maternal‐age modifier V=I(maternal age>30).

We fit the model (5) with A(D−c)V and AV interaction terms. Table 8 reports the subgroup‐specific dose–response and reference‐dose effects for younger (V=0) and older (V=1) mothers, obtained from the fitted model via the appropriate linear combinations of the estimated coefficients. Among older mothers (V=1), the estimated dose–response effects are negative across all methods (ranging from −0.79 to −1.18), and the PS–based estimates of the drinking status effect are likewise negative (ranging from −0.76 to −2.47); among younger mothers (V=0), the corresponding effects are closer to zero. This pattern is directionally consistent with prior reports that the adverse effects of PAE on cognition are concentrated among the offspring of older mothers [11, 38]. As in the homogeneous analysis, the conventional covariate adjustment estimate of the status effect among older mothers is positive, in contrast to the PS–based methods. The subgroup estimates, however, carry substantially larger standard errors than the corresponding homogeneous estimates in Table 7, particularly for the older subgroup and the weighting‐based estimators.

**TABLE 8:** Estimated subgroup effects of drinking status and log prenatal AA consumption per day, with effect modifier V=I(maternal age>30).

Method	Subgroup	D|A=1		A	
		Estimate	Standard error	Estimate	Standard error
Covariate adjustment	Younger (V=0)	0.240	0.566	0.083	1.697
	Older (V=1)	−1.177	1.205	1.727	2.492
Two–PS regression	Younger (V=0)	0.051	0.589	0.627	1.846
	Older (V=1)	−0.794	1.114	−2.465	4.776
${\text{PS}}_{I}+{\text{PS}}_{\text{II}}$	Younger (V=0)	0.206	0.581	0.600	1.649
	Older (V=1)	−0.840	1.114	−1.631	2.222
${\text{PS}}_{I}+{\text{IPW}}_{\text{II}}$	Younger (V=0)	—	—	0.971	2.127
	Older (V=1)	—	—	−2.325	5.192
${\text{PS}}_{I}+{\text{AIPW}}_{\text{II}}$	Younger (V=0)	—	—	0.441	2.778
	Older (V=1)	—	—	−0.759	3.444

*Note:* Two–PS regression: two‐dimensional PS regression adjustment; ${\text{PS}}_{I}+{\text{PS}}_{\text{II}}$: PS regression adjustment in Stages I and II; ${\text{PS}}_{I}+{\text{IPW}}_{\text{II}}$: PS regression in Stage I and IPW in Stage II; ${\text{PS}}_{I}+{\text{AIPW}}_{\text{II}}$: PS regression in Stage I and AIPW in Stage II.

Given these wide standard errors, it is natural to ask whether the maternal age is an effect modifier; if not, specification of Section 6.2 is adequate. The proposed framework makes this question directly testable: heterogeneity of the dose–response and reference‐dose effects is parameterized by the interaction coefficients $\theta_{13}$ and $\theta_{14}$ in model (5), and the absence of modification corresponds to the joint null hypothesis $H_{0}:\theta_{13}=\theta_{14}=0$. Because $\theta_{13}$ is estimated in Stage I and $\theta_{14}$ in Stage II, we base the test on the joint sandwich covariance derived in Section 3. Within each imputed dataset this yields the interaction pair $\left({\hat{\theta }}_{13},{\hat{\theta }}_{14}\right)$ and its variance–covariance matrix; we pool these across the 20 imputed datasets by Rubin's rules [35, 36] to obtain ${\hat{\theta }}_{13:14}$ and its total covariance, form the joint Wald statistic $W={\hat{\theta }}_{13:14}^{\prime }\;\hat{\mathrm{Var}}{\left({\hat{\theta }}_{13:14}\right)}^{-1}\;{\hat{\theta }}_{13:14}$, and refer it to a $\chi_{2}^{2}$ distribution. Table 9 reports the results. Across all five methods the test fails to reject the null at the 5% level, with p‐values ranging from 0.19 to 0.66, providing no significant evidence that maternal age modifies either the dose–response or the reference‐dose effect. The directional pattern in Table 8 is therefore not statistically distinguishable from the homogeneous fit, and we adopt the homogeneous specification (Table 7) as our primary analysis.

**TABLE 9:** Wald test of effect modification by maternal age, V=I(maternal age>30).

Method	Estimate (SE)		Joint Wald test		
	${\hat{\theta }}_{13}$	${\hat{\theta }}_{14}$	W	df	p
Covariate adjustment	−1.417(1.303)	1.644(2.266)	1.308	2	0.520
Two–PS regression	−0.844(1.241)	−3.092(5.387)	0.846	2	0.655
${\text{PS}}_{I}+{\text{PS}}_{\text{II}}$	−1.046(1.216)	−2.231(1.835)	3.365	2	0.186
${\text{PS}}_{I}+{\text{IPW}}_{\text{II}}$	−1.046(1.216)	−3.296(5.970)	1.208	2	0.547
${\text{PS}}_{I}+{\text{AIPW}}_{\text{II}}$	−1.046(1.216)	−1.200(4.665)	0.879	2	0.644

*Note:* Pooled standard errors in parentheses; the Stage I interaction ${\hat{\theta }}_{13}$ is common to the three two‐stage methods. Two–PS regression: two‐dimensional PS regression adjustment; ${\text{PS}}_{I}+{\text{PS}}_{\text{II}}$: PS regression adjustment in Stages I and II; ${\text{PS}}_{I}+{\text{IPW}}_{\text{II}}$: PS regression in Stage I and IPW in Stage II; ${\text{PS}}_{I}+{\text{AIPW}}_{\text{II}}$: PS regression in Stage I and AIPW in Stage II.

### 6.4 Further Considerations

To reduce the variance of the IPW and AIPW estimators, we considered trimming weights to limit the influence of extreme values. However, weight trimming is an ad hoc procedure and it can be difficult to obtain valid variance estimates, particularly for deriving asymptotic standard errors [39]. In addition, when applying Rubin's Rules to combine estimates across imputations, we found that the between‐imputation variance for Stage II IPW and AIPW was substantially larger than that observed for other approaches. One potential strategy to improve stability is to average propensity scores across multiple imputed datasets, with the aim of reducing the impact of extreme scores [40]. Motivated by this idea, we explored the use of averaged propensity scores within the Stage II IPW and AIPW procedures. Although this reduced the between‐imputation variance in our analyses, concerns have been raised about the validity of such averaging. In particular, averaging propensity scores across imputations may not yield valid balancing scores and can introduce bias, especially for weighting‐based estimators [41]. More recent work further suggests that estimating propensity scores within each imputed dataset is more reliable for achieving adequate covariate balance and producing stable estimates [42]. We therefore proceed with the conventional within‐imputation strategy: propensity scores are estimated separately within each imputed dataset, point estimates are obtained within each dataset, and Rubin's Rules are used to compute the overall variance.

As a further check on model specification, we repeated the analyses of Sections 6.2 and 6.3 including quadratic terms of the propensity scores in all regression adjustment models [25]; the resulting estimates were broadly consistent with those in Tables 7 and 8 (Supporting Information E.4), supporting the adequacy of the linear propensity score specification in this application.

The analyses reported here also differ from those in other studies of PAE using data from the Detroit cohort, reflecting differences in the outcome definition, propensity score construction, analytic strategy, and handling of missing data. With respect to the outcome, some work has used a composite cognitive score derived from a second‐order confirmatory factor analysis [16, 43] and the WISC‐III Freedom from Distractibility Index measured at age 7 [15], whereas we focus on the WISC‐III Full Scale IQ measured at age 7. In terms of modelling the semi‐continuous exposure, some approaches treat exposure as a single construct and adjust for confounding using a single two‐part propensity score [15]. In contrast, our framework decomposes exposure into status and dose components and carries out a two‐stage analysis that adjusts for separate propensity score models at each stage. We also use a slightly different set of baseline confounders in the propensity score models. In addition, our two‐part formulation allows us to model the log dose among exposed individuals directly, avoiding the need to add an arbitrary constant prior to log transformation.

A natural question is whether the two‐part exposure representation is needed in a given application. This parallels the modification analysis of Section 6.3: in both cases, the proposed framework renders a model‐simplification question empirically testable, because the status, dose, and their potential effect modification are estimated as distinct parameters. In the Detroit cohort, the estimated status and dose effects do not differ significantly, and a reduced model treating PAE as a single exposure construct yields significant evidence of an adverse effect of PAE on child cognition, consistent with previous findings [15]. Finally, with respect to missing data, we use multiple imputation to address missing values [16], whereas complete‐case analysis has also been used in this setting [15]. Our primary objective here is to illustrate the proposed methodological framework using data from the Detroit cohort; for a more comprehensive investigation of PAE effects, we refer readers to Jacobson et al. [19, 43].

## 7 Discussion

We propose a causal framework for evaluating the effects of a semi‐continuous exposure using a two‐stage estimation. In Stage I, propensity score regression adjustment targets the dose‐response effect among exposed individuals, while Stage II targets the causal reference‐dose effect compared to no exposure using an AIPW estimator that is consistent if either the Stage II propensity score or the imputation model is correctly specified, given a consistent Stage I estimate. We establish large‐sample properties for the resulting estimators, characterize their limiting values under propensity score misspecification, and assess finite‐sample performance in simulations. Taken together, these developments provide an interpretable framework that separates the consequences of any exposure from those of increasing dose, while allowing consistent estimation of the status effect when either the Stage II propensity score or the imputation model is correctly specified, provided the Stage I dose–response effect is consistently estimated.

A key limitation of the current work is that Stage I does not incorporate doubly robust estimation of the continuous exposure effect. Doubly robust approaches for continuous treatments based on kernel smoothing and flexible generalized propensity score models have been proposed and could be used to strengthen Stage I and reduce sensitivity to model misspecification [44, 45, 46]. More broadly, when Stage I yields an inconsistent estimate, consistency in Stage II generally cannot be recovered, highlighting the importance of robust modelling strategies in the first stage. Developing approaches that mitigate the impact of Stage I misspecification on Stage II estimation remains an important direction for future research.

Our framework requires specification of a reference dose c in order to define the status contrast $\Delta_{0}(c)$. This choice is necessarily subject‐matter driven and different values of c correspond to different causal questions (e.g., the effect of any exposure at a low‐dose guideline versus no exposure, or at the mean dose among exposed individuals). As we show in Section 2, under our linear MSM the choice of c does not affect the estimated dose–response slope for exposed individuals, but only re‐parameterizes the intercept term for the exposed and hence the particular reference‐dose effect being reported. In applications, we recommend that c be pre‐specified based on clinical relevance or the empirical exposure distribution, and, where feasible, that sensitivity analyses be conducted to assess the impact of alternative plausible reference levels.

In the heterogeneous treatment effect analyses, we treated the effect modifier as known. This is appropriate when there is strong scientific justification for a particular modifier, but in many applications, the relevant sources of effect heterogeneity may not be known in advance and may need to be identified from a larger set of candidate covariates. Extending the proposed framework to data‐adaptive discovery of effect modifiers is therefore an important direction for future work. Modern approaches such as causal forests and generalized random forests [47, 48] could be adapted to the semi‐continuous exposure setting to identify modifiers of both the dose–response effect among exposed individuals and the reference‐dose exposure effect.

In our application, children's cognition is assessed through multidimensional neurological outcomes in several U.S. longitudinal cohort studies, but we focused on a single outcome (WISC‐III) from the Detroit cohort. Related work has developed methods for analyzing data from multiple cohort studies while accounting for correlated outcomes, including hierarchical meta‐analysis and multi‐domain structural equation modelling with propensity score adjustment [49, 50]. Extending the proposed semi‐continuous two‐stage framework to accommodate multivariate neurological outcomes, and to settings that pool information across cohorts, is a natural next step.

Finally, the Detroit study contains missing data in baseline covariates. An additional extension would be to develop methods for evaluating effects of a semi‐continuous exposure in the presence of missing confounders, combining flexible causal inference tools with principled missing data methodology.

## Funding

This work was supported by the National Institutes of Health/National Institute on Alcohol Abuse and Alcoholism (Grant Nos. R01‐AA09524, R01‐AA025905, R01‐AA06966, and P50‐AA07606), Lycaki‐Young Fund (State of Michigan), Canadian Institutes of Health Research (Grant No. PJT‐180551), Natural Sciences and Engineering Research Council of Canada (Grant No. RGPIN‐2017‐04064), Australian Research Council Centre of Excellence for Mathematical and Statistical Frontiers (Grant No. CE140100049), and NIH/National Institute on Drug Abuse (Grant No. R21‐DA021034).

## Conflicts of Interest

The authors declare no conflicts of interest.

## Supporting information




**Table S1:** Empirical results for estimators of causal effects with P(A=1)=0.25 under six methods of analysis based on correctly specified models. **Table S2:** Empirical results for estimators of causal effects with P(A=1)=0.25 for two‐part PS regression adjustment and two‐stage analysis under model misspecification. **Table S3:** Empirical results for estimators of effects for both parts with P(A=1)=0.25 for applying PS regression adjustment in Stage I and AIPW in Stage II under model misspecification. **Table S4:** Empirical results for estimators of causal effects with P(A=1)=0.75 under six methods of analysis based on correctly specified models. **Table S5:** Empirical results for estimators of causal effects with P(A=1)=0.75 for two‐propensity‐score regression adjustment and two‐stage analysis under model misspecification. **Table S6:** Empirical results for estimators of effects for both parts with P(A=1)=0.75 for applying PS regression adjustment in Stage I and AIPW in Stage II under model misspecification. **Table S7:** Estimated effects of prenatal covariates on the continuous and binary exposure components. **Table S8:** Estimated effects of drinking status and log prenatal absolute alcohol consumption per day with quadratic propensity score terms. **Table S9:** Estimated subgroup effects of drinking status and log prenatal absolute alcohol consumption per day with quadratic propensity score terms, with effect modifier V=I(maternal age>30).

## Data Availability

The data from the Detroit Longitudinal Cohort Study that support the application in this article are not publicly available because they contain potentially identifiable participant information and are subject to ethical and data‐use restrictions. The computer code used to implement the proposed methods and reproduce the simulation studies is provided in the accompanying Data Files.
