# Supplementary material for: Two‐Stage Estimation for Causal Inference Involving Semi‐Continuous Exposures
Source: Stat Med. 2026 Oct 28;45(25-27):e70800. doi: 10.1002/sim.70800 (PMC13716696; doi:10.1002/sim.70800)
Supplement: Supplementary file 1 [file SIM-45-0-s001.zip › SupplementaryMaterial.pdf]

Received: Added at production | Revised: Added at production | Accepted: Added at production

DOI: xxx/xxxx

RESEARCH ARTICLE

Supplementary Material for "Two-stage Estimation for Causal Inference Involving Semi-Continuous Exposures"

Xiaoya Wang[1] | **Richard J. Cook**[1] | **Yeying Zhu**[1] | **Tugba Akkaya-Hocagil**[2] | **R. Colin Carter**[3] | **Sandra W. Jacobson**[4] | **Joseph L. Jacobson**[4] | **Louise M. Ryan**[5]

[1]Department of Statistics and Actuarial Science, University of Waterloo, ON, Canada

[2]Department of Biostatistics, School of Medicine, Ankara University, Ankara, Turkey

[3]Departments of Emergency Medicine and Pediatrics, Columbia University, NY, USA

[4]Department of Psychiatry and Behavioral Neurosciences, Wayne State University, MI, USA

[5]School of Mathematical and Physical Sciences, University of Technology Sydney, NSW, Australia

Correspondence
Xiaoya Wang
Email: x932wang@uwaterloo.ca

Abstract

This document includes supplementary materials.

A | PROOF OF IDENTIFICATION FOR TWO-PART PROPENSITY SCORE REGRESSION ADJUSTMENT

We consider the data generation model

$$Y = \theta_0 + \theta_{11}A(D-c) + \theta_{12}A + \boldsymbol{\theta}'_{21}\mathbf{X}_1 + \boldsymbol{\theta}'_{22}\mathbf{X}_2 + \boldsymbol{\theta}'_{23}\mathbf{X}_3 + E, \tag{A.1}$$

where $\boldsymbol{\theta}_2 = (\boldsymbol{\theta}'_{21}, \boldsymbol{\theta}'_{22}, \boldsymbol{\theta}'_{23})'$, $\mathbf{X} = (\mathbf{X}'_1, \mathbf{X}'_2, \mathbf{X}'_3)'$, and E is an error term with mean zero and independent of $(A, D, \mathbf{X})$. Here we interpret (A.1) as a structural outcome model, so that

$$Y(1,d) = \theta_0 + \theta_{11}(d-c) + \theta_{12} + \boldsymbol{\theta}'_{21}\mathbf{X}_1 + \boldsymbol{\theta}'_{22}\mathbf{X}_2 + \boldsymbol{\theta}'_{23}\mathbf{X}_3 + E,$$

and

$$Y(0,0) = \theta_0 + \boldsymbol{\theta}'_{21}\mathbf{X}_1 + \boldsymbol{\theta}'_{22}\mathbf{X}_2 + \boldsymbol{\theta}'_{23}\mathbf{X}_3 + E.$$

This structural representation is used below when relating the observed outcome to the corresponding potential outcomes. Define the two-part propensity score vector

$$\mathbf{S}(\mathbf{X};\boldsymbol{\alpha}) = (S_1(\mathbf{Z}_1;\boldsymbol{\alpha}_1),\ S_2(\mathbf{Z}_2;\boldsymbol{\alpha}_2))',$$

where $S_1(\mathbf{Z}_1;\boldsymbol{\alpha}_1) = E(D \mid A=1, \mathbf{Z}_1;\boldsymbol{\alpha}_1)$ and $S_2(\mathbf{Z}_2;\boldsymbol{\alpha}_2) = P(A=1 \mid \mathbf{Z}_2;\boldsymbol{\alpha}_2)$.

 © 2023 Copyright Holder Name

Following the balancing-score idea of Rosenbaum and Rubin [1], each component of $\mathbf{S}(\mathbf{X};\boldsymbol{\alpha})$ serves as a balancing score for its own confounder set, so that

$$D \perp \mathbf{Z}_1 \mid \{A=1,\ S_1(\mathbf{Z}_1;\boldsymbol{\alpha}_1)\}, \tag{A.2}$$
$$A \perp \mathbf{Z}_2 \mid S_2(\mathbf{Z}_2;\boldsymbol{\alpha}_2). \tag{A.3}$$

The Stage I property (A.2) follows from the dose model (6): under the normal location model $D = S_1(\mathbf{Z}_1;\boldsymbol{\alpha}_1) + W$ with $W \perp \mathbf{Z}_1 \mid A=1$, the conditional distribution of D depends on $\mathbf{Z}_1$ only through $S_1(\mathbf{Z}_1;\boldsymbol{\alpha}_1)$, and hence $D \perp \mathbf{Z}_1 \mid \{A=1, S_1(\mathbf{Z}_1;\boldsymbol{\alpha}_1)\}$ among exposed individuals. The Stage II property (A.3) is the balancing property of the propensity score $S_2 = P(A=1 \mid \mathbf{Z}_2)$ for the status model (7) [1]. For the stage-specific regression-adjustment identification argument in this section, we do not require any balancing property for the full covariate vector $\mathbf{X}$; in what follows, each stage uses only the balancing property of its corresponding component, together with the exchangeability condition in Assumption 2 for the same confounder set.

A.1 | Dose–Response Effect among the Exposed

We first consider the effect of increasing the dose D by one unit among exposed individuals. By consistency (Assumption 1) and the exchangeability condition $Y(1,d) \perp D \mid (A=1,\mathbf{Z}_1)$ in Assumption 2,

$$E\{Y \mid A=1, D=d, \mathbf{Z}_1\} = E\{Y(1,d) \mid A=1,\mathbf{Z}_1\} = \theta_0 + \theta_{12} + \theta_{11}(d-c) + \boldsymbol{\theta}_{21}'\mathbf{X}_1 + \boldsymbol{\theta}_{23}'\mathbf{X}_3 + \boldsymbol{\theta}_{22}'E\{\mathbf{X}_2 \mid A=1,\mathbf{Z}_1\}, \tag{A.4}$$

where the final expression substitutes the model (A.1) and uses that $\mathbf{X}_1$ and $\mathbf{X}_3$ are fixed given $(A=1,\mathbf{Z}_1)$ and that E has mean zero. Since the only term that depends on d is $\theta_{11}(d-c)$, we have

$$E\{Y \mid A=1, D=d+1, \mathbf{Z}_1\} - E\{Y \mid A=1, D=d, \mathbf{Z}_1\} = \theta_{11}.$$

It remains to reduce the adjustment from $\mathbf{Z}_1$ to the scalar S_1. By the balancing property (A.2), $\mathbf{Z}_1 \perp D \mid (A=1, S_1)$, and hence

$$E\{Y \mid A=1, D=d, S_1\} = E_{\mathbf{Z}_1 \mid A=1,S_1}[E\{Y \mid A=1, D=d, \mathbf{Z}_1\}] = \theta_{11}(d-c) + E_{\mathbf{Z}_1 \mid A=1,S_1}\{r(\mathbf{Z}_1)\}, \tag{A.5}$$

where $r(\mathbf{Z}_1)$ denotes the terms in (A.4) that do not depend on d. Therefore

$$E\{Y \mid A=1, D=d+1, S_1\} - E\{Y \mid A=1, D=d, S_1\} = \theta_{11} = \psi_{11},$$

so that regression adjustment on (D, S_1) among the exposed identifies the causal dose–response effect ψ_{11} in the marginal structural model (3). This derivation uses only the Stage I balancing property (A.2) and the exchangeability condition with respect to $\mathbf{Z}_1$; the confounder set $\mathbf{Z}_2$ and the component S_2 do not enter.

A.2 | Causal Effect of Reference-Dose Exposure versus No Exposure

We next consider the effect of exposure at the reference dose $D=c$ compared with no exposure. Let $\widetilde{Y} = Y - \theta_{11}A(D-c)$ denote the pseudo-outcome formed through the Stage I offset. Under (A.1) the dose term cancels, giving

$$\widetilde{Y} = \theta_0 + \theta_{12}A + \boldsymbol{\theta}_{21}'\mathbf{X}_1 + \boldsymbol{\theta}_{22}'\mathbf{X}_2 + \boldsymbol{\theta}_{23}'\mathbf{X}_3 + E.$$

By consistency, $\widetilde{Y} = Y(1,D) - \theta_{11}(D-c) = Y(1,c)$ when $A=1$ and $\widetilde{Y} = Y(0,0)$ when $A=0$; that is, $\widetilde{Y}$ coincides with the potential outcome at the reference configuration. By the exchangeability condition $\{Y(0,0), Y(1,d)\} \perp A \mid \mathbf{Z}_2$ in Assumption 2,

$$E\{\widetilde{Y} \mid A=1,\mathbf{Z}_2\} = E\{Y(1,c) \mid \mathbf{Z}_2\}, \qquad E\{\widetilde{Y} \mid A=0,\mathbf{Z}_2\} = E\{Y(0,0) \mid \mathbf{Z}_2\}, \tag{A.6}$$

so that

$$E\{\widetilde{Y} \mid A=1,\mathbf{Z}_2\} - E\{\widetilde{Y} \mid A=0,\mathbf{Z}_2\} = E\{Y(1,c) - Y(0,0) \mid \mathbf{Z}_2\} = \theta_{12},$$

where the last equality holds because $Y(1,c)-Y(0,0)=\theta_{12}$ under (A.1). Reducing the adjustment from $\mathbf{Z}_2$ to S_2, the balancing property (A.3) gives $\mathbf{Z}_2 \perp A \mid S_2$, and hence

$$E\{\widetilde{Y} \mid A=a, S_2\} = E_{\mathbf{Z}_2 \mid S_2}\left[E\{\widetilde{Y} \mid A=a, \mathbf{Z}_2\}\right], \qquad a=0,1, \tag{A.7}$$

so that

$$E\{\widetilde{Y} \mid A=1, S_2\} - E\{\widetilde{Y} \mid A=0, S_2\} = \theta_{12} = \psi_{12}.$$

Therefore regression adjustment on (A, S_2) applied to the pseudo-outcome identifies the causal effect of reference-dose exposure versus no exposure, ψ_{12}, in the marginal structural model (3). This derivation uses only the Stage II balancing property (A.3) and the exchangeability condition with respect to $\mathbf{Z}_2$; the confounder set $\mathbf{Z}_1$ and the component S_1 do not enter. In practice θ_{11} is unknown and is replaced by the consistent Stage I estimator $\hat{\gamma}_{11}$ in Theorem 1; since $\hat{\gamma}_{11} \xrightarrow{p} \psi_{11}$, the pseudo-outcome formed with the estimated offset is asymptotically equivalent, as used in the proof of Theorem 2.

Taken together, (A.4)–(A.5) and (A.6)–(A.7) show that identification proceeds one stage at a time: the dose–response effect is de-confounded using only $\mathbf{Z}_1$, equivalently S_1, and the reference-dose effect using only $\mathbf{Z}_2$, equivalently S_2. No independence or balancing condition on the full covariate vector $\mathbf{X}$ is required, which is consistent with the two-stage estimator of Section 3 and with allowing the confounder sets $\mathbf{Z}_1$ and $\mathbf{Z}_2$ to differ.

B | RESULTS ON LARGE SAMPLE PROPERTIES

The proofs of Theorems 1–3 in this section assume the additive homogeneous structural outcome model (A.1) introduced in Section A, in addition to Assumptions 1–3 and the additional conditions stated in the respective theorems.

B.1 | Proof of Theorem 1

Under Assumptions 1–3 and correct specification of the dose model (6), with an error distribution independent of $\mathbf{Z}_1$, the Stage I propensity score satisfies

$$D_i \perp\!\!\!\perp \mathbf{Z}_{i1} \mid \{A_i = 1, S_1(\mathbf{Z}_{i1}; \boldsymbol{\alpha}_1)\}. \tag{B.1}$$

Let $S_{i1} = S_1(\mathbf{Z}_{i1}; \boldsymbol{\alpha}_1)$ and $\mathbf{V}_{i1} = (1, D_i, S_{i1})'$. The Stage I estimating function is $U_{i11}(\mathcal{D}_i; \boldsymbol{\phi}_1) = A_i \mathbf{V}_{i1}\{Y_i - \mu_{i11}(\boldsymbol{\phi}_1)\}$, where $\mu_{i11}(\boldsymbol{\phi}_1) = \gamma_{10} + \gamma_{11} D_i + \gamma_{12} S_{i1}$.

We show that the population estimating equation is unbiased at a parameter value for which $\gamma_{11} = \psi_{11}$. By the law of iterated expectations,

$$\begin{aligned} &E\left\{U_{i11}(\mathcal{D}_i; \boldsymbol{\phi}_1)\right\} \\ &\quad = E\left[A_i \mathbf{V}_{i1}\left\{E(Y_i \mid A_i, D_i, S_{i1}) - \mu_{i11}(\boldsymbol{\phi}_1)\right\}\right]. \end{aligned} \tag{B.2}$$

We first characterize the conditional mean in (B.2). By consistency and the Stage I exchangeability condition in Assumption 2, $Y_i(1,d) \perp\!\!\!\perp D_i \mid (A_i = 1, \mathbf{Z}_{i1})$, we have

$$\begin{aligned} &E(Y_i \mid A_i = 1, D_i = d, \mathbf{Z}_{i1}) \\ &\quad = E\{Y_i(1,d) \mid A_i = 1, \mathbf{Z}_{i1}\} \\ &\quad = \theta_0 + \theta_{12} + \theta_{11}(d-c) + \boldsymbol{\theta}_{21}' \mathbf{X}_{i1} + \boldsymbol{\theta}_{23}' \mathbf{X}_{i3} + \boldsymbol{\theta}_{22}' E(\mathbf{X}_{i2} \mid A_i = 1, \mathbf{Z}_{i1}). \end{aligned} \tag{B.3}$$

Here $\mathbf{X}_{i1}$ and $\mathbf{X}_{i3}$ are components of $\mathbf{Z}_{i1} = (\mathbf{X}_{i1}', \mathbf{X}_{i3}')'$.

Let $r_1(\mathbf{Z}_{i1})$ denote all terms on the right-hand side of (B.3) other than $\theta_{11}(d-c)$. By the law of iterated expectations and the balancing property in (B.1),

$$\begin{aligned} &E(Y_i \mid A_i = 1, D_i = d, S_{i1}) \\ &\quad = E\left[E(Y_i \mid A_i = 1, D_i = d, \mathbf{Z}_{i1}) \mid A_i = 1, D_i = d, S_{i1}\right] \\ &\quad = \theta_{11}(d-c) + E\{r_1(\mathbf{Z}_{i1}) \mid A_i = 1, S_{i1}\}. \end{aligned} \tag{B.4}$$

The second equality in (B.4) follows because (B.1) implies that the conditional distribution of $\mathbf{Z}_{i1}$ given $(A_i = 1, D_i = d, S_{i1})$ is the same as its conditional distribution given $(A_i = 1, S_{i1})$. Therefore, (B.4) implies that

$$E(Y_i \mid A_i = 1, D_i = d+1, S_{i1}) - E(Y_i \mid A_i = 1, D_i = d, S_{i1}) = \theta_{11} = \psi_{11}. \tag{B.5}$$

It remains to link the identification result in (B.5) to the least-squares estimating equation. Let $r_1^\dagger(S_{i1})$ denote $E\{r_1(\mathbf{Z}_{i1}) \mid A_i = 1, S_{i1}\} - \theta_{11}c$. Define $(\gamma_{10}^*, \gamma_{12}^*)'$ as the population least-squares projection coefficient of $r_1^\dagger(S_{i1})$ on $(1, S_{i1})'$ among individuals with $A_i = 1$, and let $q(S_{i1}) = r_1^\dagger(S_{i1}) - \gamma_{10}^* - \gamma_{12}^* S_{i1}$. By the projection property, $E\{q(S_{i1}) \mid A_i = 1\} = 0$ and $E\{S_{i1} q(S_{i1}) \mid A_i = 1\} = 0$.

Set $\gamma_{11}^* = \theta_{11} = \psi_{11}$. It follows from (B.4) that the conditional residual from the Stage I working model is $q(S_{i1})$. The intercept and S_{i1} components of the estimating equation consequently have mean zero by the projection properties above. For the dose component, correct specification of the dose model gives $E(D_i \mid A_i = 1, \mathbf{Z}_{i1}) = S_{i1}$, and hence

$$\begin{aligned} E\{D_i q(S_{i1}) \mid A_i = 1\} &= E\left[E(D_i \mid A_i = 1, S_{i1}) q(S_{i1}) \mid A_i = 1\right] \\ &= E\{S_{i1} q(S_{i1}) \mid A_i = 1\} = 0. \end{aligned} \tag{B.6}$$

Thus, using the projection properties together with (B.6), we obtain

$$E_{\mathcal{D}_i}\left\{U_{i11}(\mathcal{D}_i; \boldsymbol{\phi}_1^*)\right\} = \mathbf{0}, \qquad \gamma_{11}^* = \theta_{11} = \psi_{11}. \tag{B.7}$$

Therefore, under Assumptions 1–3 and the correct Stage I dose model, the population estimating equation is unbiased at $\gamma_{11} = \psi_{11}$. Under standard regularity conditions for M-estimation, (B.7) implies $\hat{\gamma}_{11} \xrightarrow{p} \psi_{11}$.

B.2 | Proof of Theorem 2

Under Assumptions 1–3, and assuming that $\hat{\gamma}_{11}$ obtained from Stage I is a consistent estimator of ψ_{11}, we show that the Stage II AIPW estimating function is unbiased when either the Stage-II propensity score model or the imputation model is correctly specified.

Firstly, let

$$Y_i^\dagger = Y_i - \psi_{11} A_i (D_i - c). \tag{B.8}$$

Since $\hat{\gamma}_{11} \xrightarrow{p} \psi_{11}$, using $\hat{\gamma}_{11} A_i (D_i - c)$ as the offset is asymptotically equivalent to using $\psi_{11} A_i (D_i - c)$. Therefore, it is sufficient to establish the result using $Y_i^\dagger$.

For $a = 0, 1$, let

$$m_a^0(\mathbf{X}_i) = E\left\{Y_i^\dagger \mid A_i = a, \mathbf{X}_i\right\} \tag{B.9}$$

denote the true conditional mean of the adjusted outcome under exposure level a. Let

$$\mu_a = E\left\{m_a^0(\mathbf{X}_i)\right\}. \tag{B.10}$$

Under the structural outcome model (A.1), subtraction of the dose component gives

$$m_a^0(\mathbf{X}_i) = \theta_0 + \theta_{12} a + \boldsymbol{\theta}_{21}' \mathbf{X}_{i1} + \boldsymbol{\theta}_{22}' \mathbf{X}_{i2} + \boldsymbol{\theta}_{23}' \mathbf{X}_{i3}.$$

Consequently,

$$\mu_a = \theta_0 + \theta_{12} a + \boldsymbol{\theta}_{21}' E(\mathbf{X}_{i1}) + \boldsymbol{\theta}_{22}' E(\mathbf{X}_{i2}) + \boldsymbol{\theta}_{23}' E(\mathbf{X}_{i3}),$$

and hence $\mu_1 - \mu_0 = \theta_{12} = \psi_{12}$. Under the Stage-II marginal structural model, we parameterize these marginal means as $\mu_a = \mu_{21}(a; \boldsymbol{\gamma}_2) = \gamma_{20} + \gamma_{21} a$ for $a = 0, 1$, so that $\gamma_{21} = \mu_1 - \mu_0 = \psi_{12}$. Let $\pi_a(\mathbf{X}_i) = P(A_i = a \mid \mathbf{X}_i)$ for $a = 0, 1$. Under the Stage-II propensity score model, we define

$$\pi_a(\mathbf{Z}_{i2}; \boldsymbol{\alpha}_2) = S_2(\mathbf{Z}_{i2}; \boldsymbol{\alpha}_2)^a \{1 - S_2(\mathbf{Z}_{i2}; \boldsymbol{\alpha}_2)\}^{1-a}.$$

When the Stage-II propensity score model is correctly specified, we require

$$\pi_a(\mathbf{X}_i) = \pi_a(\mathbf{Z}_{i2}; \boldsymbol{\alpha}_2), \qquad a = 0, 1. \tag{B.11}$$

That is, conditional on $\mathbf{Z}_{i2}$, there is no remaining association between A_i and the components of $\mathbf{X}_i$ not included in $\mathbf{Z}_{i2}$.

Let $m_a(\mathbf{X}_i;\boldsymbol{\theta})$ be the working imputation model for $m_a^0(\mathbf{X}_i)$. For $a=0,1$, the AIPW estimating function for μ_a can be written as

$$\varphi_{ia}(\mu_a) = \frac{I(A_i=a)}{\pi_a(\mathbf{Z}_{i2};\boldsymbol{\alpha}_2)}\left\{Y_i^\dagger - m_a(\mathbf{X}_i;\boldsymbol{\theta})\right\} + m_a(\mathbf{X}_i;\boldsymbol{\theta}) - \mu_a. \tag{B.12}$$

The Stage-II estimating function for $\boldsymbol{\gamma}_2 = (\gamma_{20},\gamma_{21})'$ is obtained by stacking the two exposure-specific estimating functions in (B.12). Equivalently, it can be written as

$$\sum_{a=0}^{1} \frac{\partial \mu_{21}(a;\boldsymbol{\gamma}_2)}{\partial \boldsymbol{\gamma}_2}\varphi_{ia}\{\mu_{21}(a;\boldsymbol{\gamma}_2)\}. \tag{B.13}$$

The form in (B.12) is algebraically equivalent to the AIPW estimating function used in the main text. In fact, with

$$w_i(a;\boldsymbol{\alpha}_2) = \frac{I(A_i=a)}{\pi_a(\mathbf{Z}_{i2};\boldsymbol{\alpha}_2)},$$

we have

$$w_i(a;\boldsymbol{\alpha}_2)\left\{Y_i^\dagger - \mu_a\right\} - \{w_i(a;\boldsymbol{\alpha}_2)-1\}\left\{m_a(\mathbf{X}_i;\boldsymbol{\theta})-\mu_a\right\} = w_i(a;\boldsymbol{\alpha}_2)\left\{Y_i^\dagger - m_a(\mathbf{X}_i;\boldsymbol{\theta})\right\} + m_a(\mathbf{X}_i;\boldsymbol{\theta}) - \mu_a.$$

We next consider two cases.

Case 1: The imputation model is correctly specified.

Suppose that

$$m_a(\mathbf{X}_i;\boldsymbol{\theta}) = m_a^0(\mathbf{X}_i) = E\left\{Y_i^\dagger \mid A_i=a,\mathbf{X}_i\right\}, \qquad a=0,1. \tag{B.14}$$

The propensity score model may be misspecified. Let $\tilde{\pi}_a(\mathbf{Z}_{i2})$ denote the limiting propensity score used in the weights under misspecification. Then, conditional on $(A_i,\mathbf{X}_i)$,

$$E\left[\frac{I(A_i=a)}{\tilde{\pi}_a(\mathbf{Z}_{i2})}\left\{Y_i^\dagger - m_a^0(\mathbf{X}_i)\right\}\middle| A_i,\mathbf{X}_i\right] = \frac{I(A_i=a)}{\tilde{\pi}_a(\mathbf{Z}_{i2})}\left[E\left\{Y_i^\dagger \mid A_i,\mathbf{X}_i\right\} - m_a^0(\mathbf{X}_i)\right] = 0.$$

Therefore,

$$E\left\{\varphi_{ia}(\mu_a)\right\} = E\left[m_a^0(\mathbf{X}_i) - \mu_a\right] = 0,$$

by the definition of μ_a in (B.10). Thus, the AIPW estimating function is unbiased if the imputation model is correctly specified, even if the Stage-II propensity score model is misspecified.

Case 2: The propensity score model is correctly specified.

Now, suppose that the Stage-II propensity score model is correctly specified as in (B.11), but that $m_a(\mathbf{X}_i;\boldsymbol{\theta})$ may be misspecified. Taking the conditional expectation of the first term in (B.12) with respect to A_i given $\mathbf{X}_i$, we obtain

$$\begin{aligned}
&E\left[\frac{I(A_i=a)}{\pi_a(\mathbf{X}_i)}\left\{Y_i^\dagger - m_a(\mathbf{X}_i;\boldsymbol{\theta})\right\}\middle|\mathbf{X}_i\right]\\
=&E\left[\frac{I(A_i=a)}{\pi_a(\mathbf{X}_i)}E\left\{Y_i^\dagger - m_a(\mathbf{X}_i;\boldsymbol{\theta}) \mid A_i,\mathbf{X}_i\right\}\middle|\mathbf{X}_i\right]\\
=&E\left[\frac{I(A_i=a)}{\pi_a(\mathbf{X}_i)}\left\{m_a^0(\mathbf{X}_i) - m_a(\mathbf{X}_i;\boldsymbol{\theta})\right\}\middle|\mathbf{X}_i\right]\\
=&m_a^0(\mathbf{X}_i) - m_a(\mathbf{X}_i;\boldsymbol{\theta}).
\end{aligned}$$

It follows that

$$\begin{aligned} E\left\{\varphi_{ia}(\mu_a)\right\} =& E\left[m_a^0(\mathbf{X}_i) - m_a(\mathbf{X}_i;\boldsymbol{\theta}) + m_a(\mathbf{X}_i;\boldsymbol{\theta}) - \mu_a\right] \\ =& E\left\{m_a^0(\mathbf{X}_i) - \mu_a\right\} \\ =& 0. \end{aligned}$$

Thus, the AIPW estimating function is also unbiased if the Stage-II propensity score model is correctly specified, even if the imputation model is misspecified.

Therefore, provided that the Stage I estimator $\hat{\gamma}_{11}$ is consistent, the Stage II AIPW estimator is consistent for ψ_{12} if either the imputation model is correctly specified or the Stage II propensity score model is correctly specified in the sense of (B.11). When both models are correctly specified, the result follows directly from the two cases above.

B.3 | Proof of Theorem 3

Here we derive the asymptotic covariance matrix of the estimator obtained from two-stage analysis focusing on applying PS regression adjustment in stage I and use of AIPW in stage II. Recall that $\hat{\boldsymbol{\Omega}} = (\hat{\boldsymbol{\phi}}_1', \hat{\boldsymbol{\phi}}_2')'$ is the solution of

$$\bar{\bar{\mathbf{U}}}(\boldsymbol{\Omega}) = \begin{Bmatrix} \mathbf{U}_1\left(\mathcal{D};\boldsymbol{\phi}_1\right) \\ \bar{\bar{\mathbf{U}}}_2\left(\mathcal{D};\boldsymbol{\gamma}_1,\boldsymbol{\phi}_2\right) \end{Bmatrix} = \mathbf{0}.$$

where $\boldsymbol{\phi}_1 = (\boldsymbol{\gamma}_1', \boldsymbol{\alpha}_1')'$ and $\boldsymbol{\phi}_2 = (\boldsymbol{\gamma}_2', \boldsymbol{\alpha}_2', \boldsymbol{\theta}')'$.

Since

$$\bar{\bar{\mathbf{U}}}(\hat{\boldsymbol{\Omega}}) = \bar{\bar{\mathbf{U}}}(\boldsymbol{\Omega}) + \frac{\partial \bar{\bar{\mathbf{U}}}(\boldsymbol{\Omega})}{\partial \boldsymbol{\Omega}'}\left(\hat{\boldsymbol{\Omega}} - \boldsymbol{\Omega}\right) + o_p\left(\sqrt{n}\right),$$

we have

$$\sqrt{n}\left(\hat{\boldsymbol{\Omega}} - \boldsymbol{\Omega}\right) = \left\{-\frac{1}{n}\frac{\partial \bar{\bar{\mathbf{U}}}(\boldsymbol{\Omega})}{\partial \boldsymbol{\Omega}'}\right\}^{-1}\left\{\frac{1}{\sqrt{n}}\bar{\bar{\mathbf{U}}}(\boldsymbol{\Omega})\right\} + o_p(1).$$

where $\frac{\partial \bar{\bar{\mathbf{U}}}(\boldsymbol{\Omega})}{\partial \boldsymbol{\Omega}'}$ is

$$\sum_{i=1}^{n}\begin{Bmatrix} \frac{\partial \mathbf{U}_{i11}(\mathcal{D}_i;\boldsymbol{\phi}_1)}{\partial \boldsymbol{\gamma}_1} & \frac{\partial \mathbf{U}_{i11}(\mathcal{D}_i;\boldsymbol{\phi}_1)}{\partial \boldsymbol{\alpha}_1} & \mathbf{0} & \mathbf{0} & \mathbf{0} \\ \mathbf{0} & \frac{\partial \mathbf{U}_{i12}(\mathcal{D}_i;\boldsymbol{\alpha}_1)}{\partial \boldsymbol{\alpha}_1} & \mathbf{0} & \mathbf{0} & \mathbf{0} \\ \frac{\partial \bar{\bar{\mathbf{U}}}_{i21}(\mathcal{D}_i;\boldsymbol{\gamma}_1,\boldsymbol{\phi}_2)}{\partial \boldsymbol{\gamma}_1} & \mathbf{0} & \frac{\partial \bar{\bar{\mathbf{U}}}_{i21}(\mathcal{D}_i;\boldsymbol{\gamma}_1,\boldsymbol{\phi}_2)}{\partial \boldsymbol{\gamma}_2} & \frac{\partial \bar{\bar{\mathbf{U}}}_{i21}(\mathcal{D}_i;\boldsymbol{\gamma}_1,\boldsymbol{\phi}_2)}{\partial \boldsymbol{\alpha}_2} & \frac{\partial \bar{\bar{\mathbf{U}}}_{i21}(\mathcal{D}_i;\boldsymbol{\gamma}_1,\boldsymbol{\phi}_2)}{\partial \boldsymbol{\theta}} \\ \mathbf{0} & \mathbf{0} & \mathbf{0} & \frac{\partial \mathbf{U}_{i22}(\mathcal{D}_i;\boldsymbol{\alpha}_2)}{\partial \boldsymbol{\alpha}_2} & \mathbf{0} \\ \frac{\partial \mathbf{U}_{i23}(\mathcal{D}_i;\boldsymbol{\gamma}_1,\boldsymbol{\theta})}{\partial \boldsymbol{\gamma}_1} & \mathbf{0} & \mathbf{0} & \mathbf{0} & \frac{\partial \mathbf{U}_{i23}(\mathcal{D}_i;\boldsymbol{\gamma}_1,\boldsymbol{\theta})}{\partial \boldsymbol{\theta}} \end{Bmatrix}$$

As $n \to \infty$, $\mathcal{A}$ converges to

$$E\left\{-\frac{\partial \bar{\bar{\mathbf{U}}}_i(\boldsymbol{\Omega})}{\partial \boldsymbol{\Omega}'}\right\} = \begin{Bmatrix} \mathcal{A}_{11} & \mathcal{A}_{12} & \mathbf{0} & \mathbf{0} & \mathbf{0} \\ \mathbf{0} & \mathcal{A}_{22} & \mathbf{0} & \mathbf{0} & \mathbf{0} \\ \mathcal{A}_{31} & \mathbf{0} & \mathcal{A}_{33} & \mathcal{A}_{34} & \mathcal{A}_{35} \\ \mathbf{0} & \mathbf{0} & \mathbf{0} & \mathcal{A}_{44} & \mathbf{0} \\ \mathcal{A}_{51} & \mathbf{0} & \mathbf{0} & \mathbf{0} & \mathcal{A}_{55} \end{Bmatrix}$$

where

$$\mathcal{A}_{11} = E\left\{-\frac{\partial \mathbf{U}_{i11}(\mathcal{D}_i;\boldsymbol{\phi}_1)}{\partial \boldsymbol{\gamma}_1}\right\}, \mathcal{A}_{12} = E\left\{-\frac{\partial \mathbf{U}_{i11}(\mathcal{D}_i;\boldsymbol{\phi}_1)}{\partial \boldsymbol{\alpha}_1}\right\},$$

$$\mathcal{A}_{22} = E\left\{-\frac{\partial \mathbf{U}_{i12}(\boldsymbol{\alpha}_1)}{\partial \boldsymbol{\alpha}_1}\right\}, \mathcal{A}_{31} = E\left\{-\frac{\partial \bar{\bar{\mathbf{U}}}_{i21}(\mathcal{D}_i;\boldsymbol{\gamma}_1,\boldsymbol{\phi}_2)}{\partial \boldsymbol{\gamma}_1}\right\},$$

$$\mathcal{A}_{33} = E\left\{-\frac{\partial \bar{\bar{\mathbf{U}}}_{i21}(\mathcal{D}_i;\boldsymbol{\gamma}_1,\boldsymbol{\phi}_2)}{\partial \boldsymbol{\gamma}_2}\right\}, \mathcal{A}_{34} = E\left\{-\frac{\partial \bar{\bar{\mathbf{U}}}_{i21}(\mathcal{D}_i;\boldsymbol{\gamma}_1,\boldsymbol{\phi}_2)}{\partial \boldsymbol{\alpha}_2}\right\},$$

$$\mathcal{A}_{35} = E\left\{-\frac{\partial \bar{\bar{\mathbf{U}}}_{i21}(\mathcal{D}_i;\boldsymbol{\gamma}_1,\boldsymbol{\phi}_2)}{\partial \boldsymbol{\theta}}\right\}, \mathcal{A}_{44} = E\left\{-\frac{\partial \mathbf{U}_{i22}\left(\mathcal{D}_i;\boldsymbol{\alpha}_2\right)}{\partial \boldsymbol{\alpha}_2}\right\},$$

$$\mathcal{A}_{51} = E\left\{-\frac{\partial \mathbf{U}_{i23}(\mathcal{D}_i;\gamma_1,\boldsymbol{\theta})}{\partial \gamma_1}\right\}, \text{ and } \mathcal{A}_{55} = E\left\{-\frac{\partial \mathbf{U}_{i23}(\mathcal{D}_i;\gamma_1,\boldsymbol{\theta})}{\partial \boldsymbol{\theta}}\right\}.$$

As $n \to \infty$,

$$\frac{1}{n}\sum_{i=1}^{n} \bar{\bar{\mathbf{U}}}_i(\boldsymbol{\Omega})\bar{\bar{\mathbf{U}}}_i(\boldsymbol{\Omega})'$$

converges in probability to

$$E\left\{\bar{\bar{\mathbf{U}}}_i(\boldsymbol{\Omega})\bar{\bar{\mathbf{U}}}_i(\boldsymbol{\Omega})'\right\} = \mathcal{B}(\boldsymbol{\Omega})$$

Due to the consistency property of estimators for γ, we have

$$\sqrt{n}\left(\hat{\boldsymbol{\Omega}} - \boldsymbol{\Omega}\right) \xrightarrow{D} MVN\left\{\mathbf{0}, \boldsymbol{\Sigma}(\boldsymbol{\Omega})\right\}$$

where $\boldsymbol{\Sigma}(\boldsymbol{\Omega}) = \mathcal{A}^{-1}(\boldsymbol{\Omega})\mathcal{B}(\boldsymbol{\Omega})\left\{\mathcal{A}^{-1}(\boldsymbol{\Omega})\right\}'$ and

$$\mathcal{A}(\boldsymbol{\Omega}) = \begin{Bmatrix} \mathcal{A}_{11} & \mathcal{A}_{12} & \mathbf{0} & \mathbf{0} & \mathbf{0} \\ \mathbf{0} & \mathcal{A}_{22} & \mathbf{0} & \mathbf{0} & \mathbf{0} \\ \mathcal{A}_{31} & \mathbf{0} & \mathcal{A}_{33} & \mathcal{A}_{34} & \mathcal{A}_{35} \\ \mathbf{0} & \mathbf{0} & \mathbf{0} & \mathcal{A}_{44} & \mathbf{0} \\ \mathcal{A}_{51} & \mathbf{0} & \mathbf{0} & \mathbf{0} & \mathcal{A}_{55} \end{Bmatrix}.$$

B.4 | Sandwich Variance Estimator and Wald-Based Inference

For inference, we replace the population expectations in $\mathcal{A}(\cdot)$ and $\mathcal{B}(\cdot)$ with their empirical counterparts, yielding the sandwich covariance estimator

$$\hat{\boldsymbol{\Sigma}}(\hat{\boldsymbol{\Omega}}) = \hat{\mathcal{A}}^{-1}(\hat{\boldsymbol{\Omega}})\,\hat{\mathcal{B}}(\hat{\boldsymbol{\Omega}})\,\{\hat{\mathcal{A}}^{-1}(\hat{\boldsymbol{\Omega}})\}',$$

where

$$\hat{\mathcal{A}}(\hat{\boldsymbol{\Omega}}) = -\frac{1}{n}\sum_{i=1}^{n}\frac{\partial \mathbf{U}_i(\boldsymbol{\Omega})}{\partial \boldsymbol{\Omega}'}\Big|_{\boldsymbol{\Omega}=\hat{\boldsymbol{\Omega}}}, \quad \hat{\mathcal{B}}(\hat{\boldsymbol{\Omega}}) = \frac{1}{n}\sum_{i=1}^{n}\mathbf{U}_i(\hat{\boldsymbol{\Omega}})\mathbf{U}_i(\hat{\boldsymbol{\Omega}})'.$$

The estimated covariance matrix $1/n\hat{\boldsymbol{\Sigma}}(\hat{\boldsymbol{\Omega}})$ provides standard errors for all components of $\hat{\boldsymbol{\Omega}}$, from which Wald tests and confidence intervals can be constructed in the usual way.

C | PROOF OF THE BIAS INDUCED BY MISSPECIFIED PROPENSITY SCORES IN REGRESSION ADJUSTMENT

Here, we provide details of the derivations for the limiting values of the estimators under the two-stage approach when propensity score regression adjustment is applied in both stages. Under misspecification, these limiting values are pseudo-true parameter values and need not equal the causal effects of interest.

The response model is in the form of

$$Y = \theta_0 + \theta_{11}A(D-c) + \theta_{12}A + \mathbf{X}'\boldsymbol{\theta}_2 + E$$

where $E \sim N(0,\tau^2)$ and $(A,D,\mathbf{X}) \perp E$.

C.1 | Bias Caused by Misspecified Propensity Scores in Stage I

In the first stage, we let $\tilde{S}_1(\mathbf{X};\tilde{\alpha}_1)$ denote the Stage I propensity score under misspecification, and consider the regression adjustment

$$E(Y|A=1,D,\tilde{S}_1(\mathbf{X};\tilde{\boldsymbol{\alpha}}_1);\tilde{\gamma}_1) = \tilde{\gamma}_{10} + \tilde{\gamma}_{11}D + \tilde{\gamma}_{12}\tilde{S}_1(\mathbf{X};\tilde{\boldsymbol{\alpha}}_1) = \mu_{11}(\tilde{\boldsymbol{\phi}}_1),$$

where $\tilde{\gamma}_1 = (\tilde{\gamma}_{10}, \tilde{\gamma}_{11}, \tilde{\gamma}_{12})'$ and $\tilde{\boldsymbol{\phi}}_1 = (\tilde{\gamma}_1', \tilde{\boldsymbol{\alpha}}_1')'$. Here $\tilde{\gamma}_1$ is the solution to

$$E\left\{\mathcal{U}_{11}(\mathcal{D}; \tilde{\boldsymbol{\phi}}_1)\right\} = \mathbf{0} \tag{C.1}$$

where

$$\mathcal{U}_{11}(\mathcal{D}; \tilde{\boldsymbol{\phi}}_1) = A\,\frac{\partial \mu_{11}(\tilde{\boldsymbol{\phi}}_1)}{\partial \tilde{\gamma}_1}\{Y - \mu_{11}(\tilde{\boldsymbol{\phi}}_1)\}.$$

By applying the law of iterated expectations with respect to A, we obtain

$$E\{\mathcal{U}_{11}(\mathcal{D}; \tilde{\boldsymbol{\phi}}_1)\} = E\left[E\left[A\frac{\partial \mu_{11}(\tilde{\boldsymbol{\phi}}_1)}{\partial \tilde{\gamma}_1}\{Y - \mu_{11}(\tilde{\boldsymbol{\phi}}_1)\}\middle| A\right]\right].$$

Since A is binary,

$$E\left[A\frac{\partial \mu_{11}(\tilde{\boldsymbol{\phi}}_1)}{\partial \tilde{\gamma}_1}\{Y - \mu_{11}(\tilde{\boldsymbol{\phi}}_1)\}\middle| A\right] = AE\left[\frac{\partial \mu_{11}(\tilde{\boldsymbol{\phi}}_1)}{\partial \tilde{\gamma}_1}\{Y - \mu_{11}(\tilde{\boldsymbol{\phi}}_1)\}\middle| A\right].$$

Therefore,

$$E\{\mathcal{U}_{11}(\mathcal{D}; \tilde{\boldsymbol{\phi}}_1)\} = P(A=1)\,E\left[\frac{\partial \mu_{11}(\tilde{\boldsymbol{\phi}}_1)}{\partial \tilde{\gamma}_1}\{Y - \mu_{11}(\tilde{\boldsymbol{\phi}}_1)\}\middle| A=1\right].$$

Since $P(A=1) > 0$, solving the estimating equation (C.1) is equivalent to solving

$$E\left\{\mathcal{U}_{11}(\mathcal{D}; \tilde{\boldsymbol{\phi}}_1)\middle| A=1\right\} = 0.$$

Here, we let Y^* represent $Y|A=1$, D^* represent $D|A=1$, $\mathbf{X}^*$ represent $\mathbf{X}|A=1$, and $\mathcal{D}^* = (Y^*, D^*, \mathbf{X}^*)$. Now, we aim to evaluate this expectation. Firstly, we take the expectation with respect to Y^* given D^* and $\mathbf{X}^*$, then we have

$$\begin{aligned} E_{Y^*}\{\mathcal{U}_{11}(\mathcal{D}; \tilde{\boldsymbol{\phi}}_1)|D^*, \mathbf{X}^*\} &= E_{Y^*|D^*,\mathbf{X}^*}\{\mathcal{U}_{11}(\mathcal{D}; \tilde{\boldsymbol{\phi}}_1)\} \\ &= \bar{\mathbf{V}}_1^*\{E(Y^*|D^*, \mathbf{X}^*; \boldsymbol{\theta}) - \bar{\mathbf{V}}_1^{*\prime}\gamma_1\} \end{aligned} \tag{C.2}$$

where $\bar{\mathbf{V}}_1^* = \{1, D^*, \tilde{S}_1(\mathbf{X}^*; \tilde{\boldsymbol{\alpha}}_1)\}'$.

Then we take the expectation with respect to $D^*|\mathbf{X}^*$. This gives a system of equations as follows:

$$\theta_0^* + \theta_{11}E(D^*|\mathbf{X}^*) + \boldsymbol{\theta}_2'\mathbf{X}^* - \{\gamma_{10} + \gamma_{11}E(D^*|\mathbf{X}^*) + \gamma_{12}\tilde{S}_1(\mathbf{X}^*; \tilde{\boldsymbol{\alpha}}_1)\} = 0 \tag{C.3}$$

$$\begin{aligned} &\theta_0^*E(D^*|\mathbf{X}^*) + \theta_{11}E(D^{*2}|\mathbf{X}^*) + \boldsymbol{\theta}_2'E\{(D^*|\mathbf{X}^*)\mathbf{X}^*\} - \\ &[\gamma_{10}E(D^*|\mathbf{X}^*) + \gamma_{11}E(D^{*2}|\mathbf{X}^*) + \gamma_{12}E\{(D^*|\mathbf{X}^*)\tilde{S}_1(\mathbf{X}^*; \tilde{\boldsymbol{\alpha}}_1)\}] = 0 \end{aligned} \tag{C.4}$$

$$\begin{aligned} &\theta_0^*\tilde{S}_1(\mathbf{X}^*; \tilde{\boldsymbol{\alpha}}_1) + \theta_{11}E(D^*|\mathbf{X}^*)\tilde{S}_1(\mathbf{X}^*; \tilde{\boldsymbol{\alpha}}_1) + \boldsymbol{\theta}_2'\mathbf{X}^*\tilde{S}_1(\mathbf{X}^*; \tilde{\boldsymbol{\alpha}}_1) - \\ &\{\gamma_{10}\tilde{S}_1(\mathbf{X}^*; \tilde{\boldsymbol{\alpha}}_1) + \gamma_{11}E(D^*|\mathbf{X}^*)\tilde{S}_1(\mathbf{X}^*; \tilde{\boldsymbol{\alpha}}_1) + \gamma_{12}\tilde{S}_1(\mathbf{X}^*; \tilde{\boldsymbol{\alpha}}_1)^2\} = 0 \end{aligned} \tag{C.5}$$

where $\theta_0^* = \theta_0 + \theta_{12} - \theta_{11}c$. Next, we take expectation with respect to $\mathbf{X}^*$. From (C.3), we get

$$\theta_0^* + \theta_{11}E(D^*) + \boldsymbol{\theta}_2'E(\mathbf{X}^*) - [\gamma_{10} + \gamma_{11}E(D^*) + \gamma_{12}E\{\tilde{S}_1(\mathbf{X}^*; \tilde{\boldsymbol{\alpha}}_1)\}] = 0 \tag{C.6}$$

From (C.4), we get

$$\begin{aligned} &\theta_0^*E(D^*) + \theta_{11}E(D^{*2}) + \boldsymbol{\theta}_2'\{\boldsymbol{\zeta}_1(\mathbf{X}^*) + E(D^*)E(\mathbf{X}^*)\} - \\ &\left[\gamma_{10}E(D^*) + \gamma_{11}E(D^{*2}) + \gamma_{12}\left[\mathrm{cov}\{E(D^*|\mathbf{X}^*), \tilde{S}_1(\mathbf{X}^*; \tilde{\boldsymbol{\alpha}}_1)\} + E(D^*)E\{\tilde{S}_1(\mathbf{X}^*; \tilde{\boldsymbol{\alpha}}_1)\}\right]\right] = 0 \end{aligned} \tag{C.7}$$

where $\boldsymbol{\zeta}_1(\mathbf{X}^*) = \{\zeta_{11}(\mathbf{X}^*), ..., \zeta_{1k}(\mathbf{X}^*)\}'$ where $\zeta_{1j}(\mathbf{X}^*) = \mathrm{cov}\{E(D^*|\mathbf{X}^*), \mathbf{X}_j^*\}$ for $j = 1, 2, ..., k$.

From (C.5), we get

$$\theta_0^* E\{\tilde{S}_1(\mathbf{X}^*;\tilde{\boldsymbol{\alpha}}_1)\} + \theta_{11}\left[\text{cov}\{E(D^*|\mathbf{X}^*), \tilde{S}_1(\mathbf{X}^*;\tilde{\boldsymbol{\alpha}}_1)\} + E(D^*)E\{\tilde{S}_1(\mathbf{X}^*;\tilde{\boldsymbol{\alpha}}_1)\}\right]$$
$$+ \boldsymbol{\theta}_2'\left[\boldsymbol{\beta}_1(\mathbf{X}^*) + E\{\tilde{S}_1(\mathbf{X}^*;\tilde{\boldsymbol{\alpha}}_1)\}E(\mathbf{X}^*)\right]$$
$$- \left[\gamma_{10} E\{\tilde{S}_1(\mathbf{X}^*;\tilde{\boldsymbol{\alpha}}_1)\} + \gamma_{11}\left[\text{cov}\{E(D^*|\mathbf{X}^*), \tilde{S}_1(\mathbf{X}^*;\tilde{\boldsymbol{\alpha}}_1)\} + E(D^*)E\{\tilde{S}_1(\mathbf{X}^*;\tilde{\boldsymbol{\alpha}}_1)\}\right]\right.$$
$$\left. + \gamma_{12} E\{\tilde{S}_1(\mathbf{X}^*;\tilde{\boldsymbol{\alpha}}_1)^2\}\right] = 0 \tag{C.8}$$

where $\boldsymbol{\beta}_1(\mathbf{X}^*) = \{\beta_{11}(\mathbf{X}^*), ..., \beta_{1k}(\mathbf{X}^*)\}'$ where $\beta_{1j}(\mathbf{X}^*) = \text{cov}\{\tilde{S}_1(\mathbf{X}^*;\tilde{\boldsymbol{\alpha}}_1), \mathbf{X}_j^*\}$ for $j = 1, 2, ..., k$. Then from (C.6), we can obtain the form of γ_{10}:

$$\gamma_{10} = \theta_0^* + \theta_{11}E(D^*) + \boldsymbol{\theta}_2' E(\mathbf{X}^*) - \gamma_{11}E(D^*) - \gamma_{12}E\{\tilde{S}_1(\mathbf{X}^*;\tilde{\boldsymbol{\alpha}}_1)\}$$

Then, we replace γ_{10} in (C.7)

$$\theta_0^* E(D^*) + \theta_{11}E(D^{*2}) + \boldsymbol{\theta}_2'\{\boldsymbol{\zeta}_1(\mathbf{X}^*) + E(D^*)E(\mathbf{X}^*)\} -$$
$$\left[\left[\theta_0^* + \theta_{11}E(D^*) + \boldsymbol{\theta}_2' E(\mathbf{X}^*) - \gamma_{11}E(D^*) - \gamma_{12}E\{\tilde{S}_1(\mathbf{X}^*;\tilde{\boldsymbol{\alpha}}_1)\}\right] E(D^*)\right.$$
$$\left. + \gamma_{11}E(D^{*2}) + \gamma_{12}\left[\text{cov}\{E(D^*|\mathbf{X}^*), \tilde{S}_1(\mathbf{X}^*;\tilde{\boldsymbol{\alpha}}_1)\} + E(D^*)E\{\tilde{S}_1(\mathbf{X}^*;\tilde{\boldsymbol{\alpha}}_1)\}\right]\right] = 0$$

After some algebra, we get

$$\theta_{11}[E(D^{*2}) - E(D^*)^2] + \boldsymbol{\theta}_2'\boldsymbol{\zeta}_1(\mathbf{X}^*) - \gamma_{11}[E(D^{*2}) - E(D^*)^2]$$
$$- \gamma_{12}\text{cov}\left[E(D^*|\mathbf{X}^*), \tilde{S}_1(\mathbf{X}^*;\tilde{\boldsymbol{\alpha}}_1)\right] = 0. \tag{C.9}$$

After replacing γ_{10} in (C.8), we get

$$\theta_0^* E\{\tilde{S}_1(\mathbf{X}^*;\tilde{\boldsymbol{\alpha}}_1)\} + \theta_{11}\left[\text{cov}\{E(D^*|\mathbf{X}^*), \tilde{S}_1(\mathbf{X}^*;\tilde{\boldsymbol{\alpha}}_1)\} + E(D^*)E\{\tilde{S}_1(\mathbf{X}^*;\tilde{\boldsymbol{\alpha}}_1)\}\right]$$
$$+ \boldsymbol{\theta}_2'\left[\boldsymbol{\beta}_1(\mathbf{X}^*) + E\{\tilde{S}_1(\mathbf{X}^*;\tilde{\boldsymbol{\alpha}}_1)\}E(\mathbf{X}^*)\right]$$
$$- \left[\left[\theta_0^* + \theta_{11}E(D^*) + \boldsymbol{\theta}_2' E(\mathbf{X}^*) - \gamma_{11}E(D^*) - \gamma_{12}E\{\tilde{S}_1(\mathbf{X}^*;\tilde{\boldsymbol{\alpha}}_1)\}\right] E\{\tilde{S}_1(\mathbf{X}^*;\tilde{\boldsymbol{\alpha}}_1)\}\right.$$
$$\left. + \gamma_{11}\left[\text{cov}\{E(D^*|\mathbf{X}^*), \tilde{S}_1(\mathbf{X}^*;\tilde{\boldsymbol{\alpha}}_1)\} + E(D^*)E\{\tilde{S}_1(\mathbf{X}^*;\tilde{\boldsymbol{\alpha}}_1)\}\right] + \gamma_{12}E\left[\tilde{S}_1(\mathbf{X}^*;\tilde{\boldsymbol{\alpha}}_1)^2\right]\right] = 0. \tag{C.10}$$

Similarly, after some algebra, from (C.10) we finally get

$$\theta_{11}\text{cov}\{E(D^*|\mathbf{X}^*), \tilde{S}_1(\mathbf{X}^*;\tilde{\boldsymbol{\alpha}}_1)\} + \boldsymbol{\theta}_2'\boldsymbol{\beta}_1(\mathbf{X}^*) - \gamma_{11}\text{cov}\{E(D^*|\mathbf{X}^*), \tilde{S}_1(\mathbf{X}^*;\tilde{\boldsymbol{\alpha}}_1)\}$$
$$- \gamma_{12}\left\{E\{\tilde{S}_1(\mathbf{X}^*;\tilde{\boldsymbol{\alpha}}_1)^2\} - E\{\tilde{S}_1(\mathbf{X}^*;\tilde{\boldsymbol{\alpha}}_1)\}^2\right\} = 0$$

Then we can obtain γ_{12} expressed as

$$\gamma_{12} = \frac{\left[(\theta_{11} - \gamma_{11})\text{cov}\{E(D^*|\mathbf{X}^*), \tilde{S}_1(\mathbf{X}^*;\tilde{\boldsymbol{\alpha}}_1)\} + \boldsymbol{\theta}_2'\boldsymbol{\beta}_1(\mathbf{X}^*)\right]}{\text{var}\{\tilde{S}_1(\mathbf{X}^*;\tilde{\boldsymbol{\alpha}}_1)\}}. \tag{C.11}$$

By replacing γ_{12} in (C.9), we get

$$\theta_{11}\{E(D^{*2}) - E(D^*)^2\} + \boldsymbol{\theta}_2'\boldsymbol{\zeta}_1(\mathbf{X}^*) - \gamma_{11}\{E(D^{*2}) - E(D^*)^2\}$$
$$- \frac{\left[(\theta_{11} - \gamma_{11})\text{cov}\{E(D^*|\mathbf{X}^*), \tilde{S}_1(\mathbf{X}^*;\tilde{\boldsymbol{\alpha}}_1)\} + \boldsymbol{\theta}_2'\boldsymbol{\beta}_1(\mathbf{X}^*)\right]}{\text{var}\{\tilde{S}_1(\mathbf{X}^*;\tilde{\boldsymbol{\alpha}}_1)\}}\text{cov}\{E(D^*|\mathbf{X}^*), \tilde{S}_1(\mathbf{X}^*;\tilde{\boldsymbol{\alpha}}_1)\} = 0.$$

After simplifying, we obtain

$$\gamma_{11} = \theta_{11} + \frac{\boldsymbol{\theta}_2' \left[\boldsymbol{\zeta}_1(\mathbf{X}^*) - \boldsymbol{\beta}_1(\mathbf{X}^*)\mathrm{corr}\left\{E(D^*|\mathbf{X}^*), \tilde{S}_1(\mathbf{X}^*;\tilde{\boldsymbol{\alpha}}_1)\right\}\sqrt{\frac{\mathrm{var}\{E(D^*|\mathbf{X}^*)\}}{\mathrm{var}\{\tilde{S}_1(\mathbf{X}^*;\tilde{\boldsymbol{\alpha}}_1)\}}}\right]}{\mathrm{var}(D^*) - \mathrm{var}\left\{E(D^*|\mathbf{X}^*)\right\}\mathrm{corr}^2\left\{E(D^*|\mathbf{X}^*), \tilde{S}_1(\mathbf{X}^*;\tilde{\boldsymbol{\alpha}}_1)\right\}}$$

$$= \theta_{11} + \frac{\boldsymbol{\theta}_2' \left[\boldsymbol{\zeta}_1(\mathbf{X}|A=1) - \boldsymbol{\beta}_1(\mathbf{X}|A=1)\mathrm{corr}\left[E(D|\mathbf{X},A=1), \tilde{S}_1(\mathbf{X};\tilde{\boldsymbol{\alpha}}_1)\right]\sqrt{\frac{\mathrm{var}\{E(D|\mathbf{X},A=1)\}}{\mathrm{var}\{\tilde{S}_1(\mathbf{X};\tilde{\boldsymbol{\alpha}}_1)\}}}\right]}{\mathrm{var}(D|A=1) - \mathrm{var}\left\{E(D|\mathbf{X},A=1)\right\}\mathrm{corr}^2\left\{E(D|\mathbf{X},A=1), \tilde{S}_1(\mathbf{X};\tilde{\boldsymbol{\alpha}}_1)\right\}}$$

To make the form of the limiting value more clear, we rewrite it in the form of

$$\gamma_{11} = \theta_{11} + \frac{\boldsymbol{\theta}_2' \left[\boldsymbol{\zeta}_1(\mathbf{X}|A=1) - \boldsymbol{\beta}_1(\mathbf{X}|A=1)\rho_1\sqrt{\frac{\mathrm{var}\{E(D|\mathbf{X},A=1)\}}{\mathrm{var}\{\tilde{S}_1(\mathbf{X};\tilde{\boldsymbol{\alpha}}_1)\}}}\right]}{\mathrm{var}(D|A=1) - \mathrm{var}\left\{E(D|\mathbf{X},A=1)\right\}\rho_1^2}$$

where $\rho_1 = \mathrm{corr}\left\{E(D|\mathbf{X},A=1), \tilde{S}_1(\mathbf{X};\tilde{\boldsymbol{\alpha}}_1)\right\}$.

C.2 | Bias Caused by Misspecified Propensity Scores in Stage II

Let $\tilde{S}_2(\mathbf{X};\tilde{\boldsymbol{\alpha}}_2)$ be the Stage II propensity score under misspecification. We fit the propensity score regression adjustment model

$$E\left\{Y|D,A,\tilde{S}_2(\mathbf{X};\tilde{\boldsymbol{\alpha}}_2);\tilde{\gamma}_1,\tilde{\boldsymbol{\gamma}}_2\right\} = \tilde{\gamma}_{20} + \tilde{\gamma}_{21}A + \tilde{\gamma}_{22}\tilde{S}_2(\mathbf{X};\tilde{\boldsymbol{\alpha}}_2) + \text{offset}\left\{\tilde{\gamma}_{11}A(D-c)\right\}$$

where $\tilde{\boldsymbol{\gamma}}_2 = (\tilde{\gamma}_{20},\tilde{\gamma}_{21},\tilde{\gamma}_{22})'$. The estimating equation for $\tilde{\boldsymbol{\gamma}}_2$ is

$$E\left\{\mathcal{U}_{21}(\mathcal{D};\tilde{\gamma}_1,\tilde{\boldsymbol{\phi}}_2)\right\} = \mathbf{0} \tag{C.12}$$

where

$$\mathcal{U}_{21}(\mathcal{D};\tilde{\gamma}_1,\tilde{\boldsymbol{\phi}}_2) = \frac{\partial\mu_{21}(\tilde{\boldsymbol{\phi}}_2)}{\partial\tilde{\boldsymbol{\gamma}}_2}\left[Y - \left\{\mu_{21}(\tilde{\boldsymbol{\phi}}_2) + \tilde{\gamma}_{11}A(D-c)\right\}\right]$$

with $\mu_{21}(\tilde{\boldsymbol{\phi}}_2) = \tilde{\gamma}_{20} + \tilde{\gamma}_{21}A + \tilde{\gamma}_{22}\tilde{S}_2(\mathbf{X};\tilde{\boldsymbol{\alpha}}_2)$.

We let $\bar{\mathbf{V}}_2 = \{1, A, \tilde{S}_2(\mathbf{X};\tilde{\boldsymbol{\alpha}}_2)\}'$, the estimating function for $\boldsymbol{\gamma}_2$ is expressed as

$$\mathcal{U}_{21}(\mathcal{D};\tilde{\gamma}_1,\tilde{\boldsymbol{\phi}}_2) = \bar{\mathbf{V}}_2\left\{Y - \tilde{\gamma}_{11}A(D-c) - \bar{\mathbf{V}}_2'\boldsymbol{\gamma}_2\right\}.$$

We obtain $\hat{\boldsymbol{\gamma}}_2$ by solving the equation

$$E\{\mathcal{U}_{21}(\mathcal{D};\tilde{\gamma}_1,\tilde{\boldsymbol{\phi}}_2)\} = \mathbf{0}$$

Firstly, we take expectation with respect to $Y|D,A,\mathbf{X}$ and we get

$$E_{Y|D,A,\mathbf{X}}\left\{\mathcal{U}_{21}(\mathcal{D};\tilde{\gamma}_1,\tilde{\boldsymbol{\phi}}_2)\right\} = \bar{\mathbf{V}}_2\left\{E(Y|D,A,\mathbf{X};\boldsymbol{\theta}) - \tilde{\gamma}_{11}A(D-c) - \bar{\mathbf{V}}_2'\boldsymbol{\gamma}_2\right\}. \tag{C.13}$$

Then we take expectation with respect to $D,A|\mathbf{X}$ and we can get a 3×1 vector expressed as

$$\theta_0 + \theta_{12}E(A|\mathbf{X}) + (\theta_{11} - \tilde{\gamma}_{11})\left\{E(AD|\mathbf{X}) - cE(A|\mathbf{X})\right\} + \boldsymbol{\theta}_2'\mathbf{X}$$
$$- \{\gamma_{20} + \gamma_{21}E(A|\mathbf{X}) + \gamma_{22}\tilde{S}_2(\mathbf{X};\tilde{\boldsymbol{\alpha}}_2)\} = 0 \tag{C.14}$$

$$\theta_0 E(A|\mathbf{X}) + \theta_{12}E(A^2|\mathbf{X}) + (\theta_{11} - \tilde{\gamma}_{11})\left\{E(A^2D|\mathbf{X}) - cE(A^2|\mathbf{X})\right\} + \boldsymbol{\theta}_2'E\left\{(A|\mathbf{X})\mathbf{X}\right\}$$
$$- \left[\gamma_{20}E(A|\mathbf{X}) + \gamma_{21}E(A^2|\mathbf{X}) + \gamma_{22}E\left\{(A|\mathbf{X})\tilde{S}_2(\mathbf{X};\tilde{\boldsymbol{\alpha}}_2)\right\}\right] = 0 \tag{C.15}$$

$$\theta_0\tilde{S}_2(\mathbf{X};\tilde{\boldsymbol{\alpha}}_2) + \theta_{12}E\left\{(A|\mathbf{X})\tilde{S}_2(\mathbf{X};\tilde{\boldsymbol{\alpha}}_2)\right\} + (\theta_{11} - \tilde{\gamma}_{11})\left\{E(AD|\mathbf{X}) - cE(A|\mathbf{X})\right\}\tilde{S}_2(\mathbf{X};\tilde{\boldsymbol{\alpha}}_2)$$
$$+ \boldsymbol{\theta}_2'\tilde{S}_2(\mathbf{X};\tilde{\boldsymbol{\alpha}}_2)\mathbf{X} - \left[\tilde{S}_2(\mathbf{X};\tilde{\boldsymbol{\alpha}}_2)\gamma_{20} + \gamma_{21}E\left\{(A|\mathbf{X})\tilde{S}_2(\mathbf{X};\tilde{\boldsymbol{\alpha}}_2)\right\} + \gamma_{22}\tilde{S}_2(\mathbf{X};\tilde{\boldsymbol{\alpha}}_2)^2\right] = 0. \tag{C.16}$$

Our next step is to take expectation with respect to $\mathbf{X}$. From (C.14) we get

$$\theta_0 + \theta_{12}E(A) + (\theta_{11} - \tilde{\gamma}_{11})\left\{E(AD) - cE(A)\right\} + \boldsymbol{\theta}_2'E(\mathbf{X}) - \left[\gamma_{20} + \gamma_{21}E(A) + \gamma_{22}E\left\{\tilde{S}_2(\mathbf{X};\tilde{\boldsymbol{\alpha}}_2)\right\}\right] = 0 \tag{C.17}$$

From (C.15) we get

$$\theta_0 E(A) + \theta_{12} E(A^2) + (\theta_{11} - \tilde{\gamma}_{11})\{E(A^2 D) - cE(A^2)\} + \boldsymbol{\theta}_2'\{\boldsymbol{\zeta}_2(\mathbf{X}) + E(A)E(\mathbf{X})\}$$
$$- \left[\gamma_{20} E(A) + \gamma_{21} E(A^2) + \gamma_{22}\left[\mathrm{cov}\{E(A|\mathbf{X}), \tilde{S}_2(\mathbf{X};\tilde{\boldsymbol{\alpha}}_2)\} + E(A)E\{\tilde{S}_2(\mathbf{X};\tilde{\boldsymbol{\alpha}}_2)\}\right]\right] = 0 \quad \text{(C.18)}$$

where $\boldsymbol{\zeta}_2(\mathbf{X}) = \{\zeta_{21}(\mathbf{X}), \ldots, \zeta_{2k}(\mathbf{X})\}'$ where $\zeta_{2j}(\mathbf{X}) = \mathrm{cov}\{E(A|\mathbf{X}), \mathbf{X}_j\}$ for $j = 1, 2, ..., k$.

From (C.16) we get

$$\theta_0 E\{\tilde{S}_2(\mathbf{X};\tilde{\boldsymbol{\alpha}}_2)\} + \theta_{12}\left[\mathrm{cov}\{E(A|\mathbf{X}), \tilde{S}_2(\mathbf{X};\tilde{\boldsymbol{\alpha}}_2)\} + E(A)E\{\tilde{S}_2(\mathbf{X};\tilde{\boldsymbol{\alpha}}_2)\}\right]$$
$$+ (\theta_{11} - \tilde{\gamma}_{11})\Big[\mathrm{cov}\{E(AD|\mathbf{X}), \tilde{S}_2(\mathbf{X};\tilde{\boldsymbol{\alpha}}_2)\} + E(AD)E\{\tilde{S}_2(\mathbf{X};\tilde{\boldsymbol{\alpha}}_2)\}$$
$$- c\Big[\mathrm{cov}\{E(A|\mathbf{X}), \tilde{S}_2(\mathbf{X};\tilde{\boldsymbol{\alpha}}_2)\} + E(A)E\{\tilde{S}_2(\mathbf{X};\tilde{\boldsymbol{\alpha}}_2)\}\Big]\Big]$$
$$+ \boldsymbol{\theta}_2'\left[\mathrm{cov}\{\tilde{S}_2(\mathbf{X};\tilde{\boldsymbol{\alpha}}_2), \mathbf{X}\} + E\{\tilde{S}_2(\mathbf{X};\tilde{\boldsymbol{\alpha}}_2)\}E(\mathbf{X})\right]$$
$$- \left[\gamma_{20} E\{\tilde{S}_2(\mathbf{X};\tilde{\boldsymbol{\alpha}}_2)\} + \gamma_{21}\left[\mathrm{cov}\{E(A|\mathbf{X}), \tilde{S}_2(\mathbf{X};\tilde{\boldsymbol{\alpha}}_2)\} + E(A)E\{\tilde{S}_2(\mathbf{X};\tilde{\boldsymbol{\alpha}}_2)\}\right] + \gamma_{22} E\{\tilde{S}_2(\mathbf{X};\tilde{\boldsymbol{\alpha}}_2)^2\}\right] = 0. \quad \text{(C.19)}$$

Then from (C.17), we obtain γ_{20}:

$$\gamma_{20} = \theta_0 + \theta_{12} E(A) + (\theta_{11} - \tilde{\gamma}_{11})\{E(AD) - cE(A)\} + \boldsymbol{\theta}_2' E(\mathbf{X}) - \gamma_{21} E(A) - \gamma_{22} E\{\tilde{S}_2(\mathbf{X};\tilde{\boldsymbol{\alpha}}_2)\}$$

We replace γ_{20} in (C.18) and get:

$$\theta_0 E(A) + \theta_{12} E(A^2) + (\theta_{11} - \tilde{\gamma}_{11})\{E(A^2 D) - cE(A^2)\} + \boldsymbol{\theta}_2'\{\boldsymbol{\zeta}_2(\mathbf{X}) + E(A)E(\mathbf{X})\}$$
$$- \left[\theta_0 + \theta_{12} E(A) + (\theta_{11} - \tilde{\gamma}_{11})\{E(AD) - cE(A)\} + \boldsymbol{\theta}_2' E(\mathbf{X}) - \gamma_{21} E(A) - \gamma_{22} E\{\tilde{S}_2(\mathbf{X};\tilde{\boldsymbol{\alpha}}_2)\}\right] E(A)$$
$$- \gamma_{21} E(A^2) - \gamma_{22}\left[\mathrm{cov}\{E(A|\mathbf{X}), \tilde{S}_2(\mathbf{X};\tilde{\boldsymbol{\alpha}}_2)\} + E(A)E\{\tilde{S}_2(\mathbf{X};\tilde{\boldsymbol{\alpha}}_2)\}\right] = 0.$$

After algebra, we get

$$\theta_{12}\{E(A^2) - E(A)^2\} + \boldsymbol{\theta}_2'\boldsymbol{\zeta}_2(\mathbf{X}) - \gamma_{21}\{E(A^2) - E(A)^2\} - \gamma_{22}\mathrm{cov}\{E(A|\mathbf{X}), \tilde{S}_2(\mathbf{X};\tilde{\boldsymbol{\alpha}}_2)\}$$
$$+ (\theta_{11} - \tilde{\gamma}_{11})\{E(A^2 D) - E(AD)E(A) + cE(A)^2 - cE(A^2)\} = 0. \quad \text{(C.20)}$$

To solve for γ_{22}, we replace γ_{20} in (C.19) to get

$$\theta_0 E\{\tilde{S}_2(\mathbf{X};\tilde{\boldsymbol{\alpha}}_2)\} + \theta_{12}\left[\mathrm{cov}\{E(A|\mathbf{X}), \tilde{S}_2(\mathbf{X};\tilde{\boldsymbol{\alpha}}_2)\} + E(A)E\{\tilde{S}_2(\mathbf{X};\tilde{\boldsymbol{\alpha}}_2)\}\right]$$
$$+ (\theta_{11} - \tilde{\gamma}_{11})\Big[\mathrm{cov}\{E(AD|\mathbf{X}), \tilde{S}_2(\mathbf{X};\tilde{\boldsymbol{\alpha}}_2)\} + E(AD)E\{\tilde{S}_2(\mathbf{X};\tilde{\boldsymbol{\alpha}}_2)\}$$
$$- c\Big[\mathrm{cov}\{E(A|\mathbf{X}), \tilde{S}_2(\mathbf{X};\tilde{\boldsymbol{\alpha}}_2)\} + E(A)E\{\tilde{S}_2(\mathbf{X};\tilde{\boldsymbol{\alpha}}_2)\}\Big]\Big]$$
$$+ \boldsymbol{\theta}_2'\left[\mathrm{cov}\{\tilde{S}_2(\mathbf{X};\tilde{\boldsymbol{\alpha}}_2), \mathbf{X}\} + E\{\tilde{S}_2(\mathbf{X};\tilde{\boldsymbol{\alpha}}_2)\}E(\mathbf{X})\right]$$
$$- E\{\tilde{S}_2(\mathbf{X};\tilde{\boldsymbol{\alpha}}_2)\}\left[\theta_0 + \theta_{12} E(A) + (\theta_{11} - \tilde{\gamma}_{11})\{E(AD) - cE(A)\} + \boldsymbol{\theta}_2' E(\mathbf{X}) - \gamma_{21} E(A) - \gamma_{22} E\{\tilde{S}_2(\mathbf{X};\tilde{\boldsymbol{\alpha}}_2)\}\right]$$
$$- \gamma_{21}\left[\mathrm{cov}\{E(A|\mathbf{X}), \tilde{S}_2(\mathbf{X};\tilde{\boldsymbol{\alpha}}_2)\}\right] - \gamma_{22} E\{\tilde{S}_2(\mathbf{X};\tilde{\boldsymbol{\alpha}}_2)^2\} = 0.$$

We simplify it to get

$$\theta_{12}\mathrm{cov}\{E(A|\mathbf{X}), \tilde{S}_2(\mathbf{X};\tilde{\boldsymbol{\alpha}}_2)\} + \boldsymbol{\theta}_2'\mathrm{cov}\{\tilde{S}_2(\mathbf{X};\tilde{\boldsymbol{\alpha}}_2), \mathbf{X}\} - \gamma_{21}\mathrm{cov}\{E(A|\mathbf{X}), \tilde{S}_2(\mathbf{X};\tilde{\boldsymbol{\alpha}}_2)\}$$
$$- \gamma_{22}\left[E\{\tilde{S}_2(\mathbf{X};\tilde{\boldsymbol{\alpha}}_2)^2\} - E\{\tilde{S}_2(\mathbf{X};\tilde{\boldsymbol{\alpha}}_2)\}^2\right]$$
$$+ (\theta_{11} - \tilde{\gamma}_{11})\left[\mathrm{cov}\{E(AD|\mathbf{X}), \tilde{S}_2(\mathbf{X};\tilde{\boldsymbol{\alpha}}_2)\} - c \cdot \mathrm{cov}\{E(A|\mathbf{X}), \tilde{S}_2(\mathbf{X};\tilde{\boldsymbol{\alpha}}_2)\}\right] = 0.$$

Then we can obtain γ_{22} expressed by

$$\gamma_{22} = \frac{(\theta_{12} - \gamma_{21})\delta_1 + \boldsymbol{\theta}_2'\mathrm{cov}\{\tilde{S}_2(\mathbf{X};\tilde{\boldsymbol{\alpha}}_2), \mathbf{X}\} + (\theta_{11} - \tilde{\gamma}_{11})(\delta_2 - c\delta_1)}{\mathrm{var}\{\tilde{S}_2(\mathbf{X};\tilde{\boldsymbol{\alpha}}_2)\}} \quad \text{(C.21)}$$

where $\delta_1 = \text{cov}\{E(A|\mathbf{X}), \tilde{S}_2(\mathbf{X}; \tilde{\boldsymbol{\alpha}}_2)\}$ and $\delta_2 = \text{cov}\{E(AD|\mathbf{X}), \tilde{S}_2(\mathbf{X}; \tilde{\boldsymbol{\alpha}}_2)\}$. Next, we substitute γ_{22} in (C.20):

$$\begin{aligned}&\theta_{12}\{E(A^2) - E(A)^2\} + \boldsymbol{\theta}_2'\boldsymbol{\zeta}_2(\mathbf{X}) - \gamma_{21}\{E(A^2) - E(A)^2\}\\&- \frac{(\theta_{12} - \gamma_{21})\delta_1 + \boldsymbol{\theta}_2'\text{cov}\{\tilde{S}_2(\mathbf{X}; \tilde{\boldsymbol{\alpha}}_2), \mathbf{X}\} + (\theta_{11} - \tilde{\gamma}_{11})(\delta_2 - c\delta_1)}{\text{var}\{\tilde{S}_2(\mathbf{X}; \tilde{\boldsymbol{\alpha}}_2)\}}\delta_1\\&+ (\theta_{11} - \tilde{\gamma}_{11})\{E(A^2D) - E(AD)E(A) + cE(A)^2 - cE(A^2)\} = 0.\end{aligned}$$

By simplifying, we get

$$\begin{aligned}&\theta_{12}\text{var}(A) + \boldsymbol{\theta}_2'\boldsymbol{\zeta}_2(\mathbf{X}) - \gamma_{21}\text{var}(A)\\&- \frac{(\theta_{12} - \gamma_{21})\delta_1 + \boldsymbol{\theta}_2'\text{cov}\{\tilde{S}_2(\mathbf{X}; \tilde{\boldsymbol{\alpha}}_2), \mathbf{X}\} + (\theta_{11} - \tilde{\gamma}_{11})(\delta_2 - c\delta_1)}{\text{var}\{\tilde{S}_2(\mathbf{X}; \tilde{\boldsymbol{\alpha}}_2)\}}\delta_1\\&+ (\theta_{11} - \tilde{\gamma}_{11})\{\text{cov}(AD, A) - c\text{var}(A)\} = 0.\end{aligned}$$

Then we re-organize it and rewrite it as

$$\begin{aligned}&\theta_{12}\left[\text{var}(A) - \frac{\delta_1^2}{\text{var}\{\tilde{S}_2(\mathbf{X}; \tilde{\boldsymbol{\alpha}}_2)\}}\right] + \boldsymbol{\theta}_2'\left[\boldsymbol{\zeta}_2(\mathbf{X}) - \frac{\text{cov}\{\tilde{S}_2(\mathbf{X}; \tilde{\boldsymbol{\alpha}}_2), \mathbf{X}\}\delta_1}{\text{var}\{\tilde{S}_2(\mathbf{X}; \tilde{\boldsymbol{\alpha}}_2)\}}\right]\\&+ (\theta_{11} - \tilde{\gamma}_{11})\left[\text{cov}(AD, A) - c\text{var}(A) - \frac{\delta_1\delta_2 - c\delta_1^2}{\text{var}\{\tilde{S}_2(\mathbf{X}; \tilde{\boldsymbol{\alpha}}_2)\}}\right] = \gamma_{21}\left[\text{var}(A) - \frac{\delta_1^2}{\text{var}\{\tilde{S}_2(\mathbf{X}; \tilde{\boldsymbol{\alpha}}_2)\}}\right].\end{aligned} \tag{C.22}$$

Here we let

$$\rho_{21} = \text{corr}\{E(A|\mathbf{X}), \tilde{S}_2(\mathbf{X}; \tilde{\boldsymbol{\alpha}}_2)\};\ \rho_{22} = \text{corr}\{E(AD|\mathbf{X}), \tilde{S}_2(\mathbf{X}; \tilde{\boldsymbol{\alpha}}_2)\},$$

so that $\delta_1 = \rho_{21}\left[\text{var}\{E(A|\mathbf{X})\}\text{var}\{\tilde{S}_2(\mathbf{X}; \tilde{\boldsymbol{\alpha}}_2)\}\right]^{1/2}$ and $\delta_2 = \rho_{22}\left[\text{var}\{E(AD|\mathbf{X})\}\text{var}\{\tilde{S}_2(\mathbf{X}; \tilde{\boldsymbol{\alpha}}_2)\}\right]^{1/2}$. We now simplify some terms in (C.22), using these expressions together with the variance decomposition $\text{var}(A) = E\{\text{var}(A|\mathbf{X})\} + \text{var}\{E(A|\mathbf{X})\}$:

$$\begin{aligned}&\theta_{12}\left[\text{var}(A) - \frac{\delta_1^2}{\text{var}\{\tilde{S}_2(\mathbf{X}; \tilde{\boldsymbol{\alpha}}_2)\}}\right] = \theta_{12}\left[\text{var}(A) - \text{var}\{E(A|\mathbf{X})\}\rho_{21}^2\right],\\&(\theta_{11} - \tilde{\gamma}_{11})\left[\text{cov}(AD, A) - c\text{var}(A) - \frac{\delta_1\delta_2 - c\delta_1^2}{\text{var}\{\tilde{S}_2(\mathbf{X}; \tilde{\boldsymbol{\alpha}}_2)\}}\right]\\&= (\theta_{11} - \tilde{\gamma}_{11})\left[\text{cov}(AD, A) - c\left[E\{\text{var}(A|\mathbf{X})\} + (1 - \rho_{21}^2)\text{var}\{E(A|\mathbf{X})\}\right] - \rho_{21}\rho_{22}\sqrt{\text{var}\{E(A|\mathbf{X})\}\text{var}\{E(AD|\mathbf{X})\}}\right],\\&\gamma_{21}\left[\text{var}(A) - \frac{\delta_1^2}{\text{var}\{\tilde{S}_2(\mathbf{X}; \tilde{\boldsymbol{\alpha}}_2)\}}\right] = \gamma_{21}\left[\text{var}(A) - \text{var}\{E(A|\mathbf{X})\}\rho_{21}^2\right].\end{aligned}$$

Let

$$\boldsymbol{\beta}_2(\mathbf{X}) = \{\beta_{21}(\mathbf{X}), ..., \beta_{2k}(\mathbf{X})\}'$$

where $\beta_{2j}(\mathbf{X}) = \text{cov}\{\tilde{S}_2(\mathbf{X}; \tilde{\boldsymbol{\alpha}}_2), \mathbf{X}_j\}$ for $j = 1, 2, ..., k$. Then (C.22) can be expressed as

$$\begin{aligned}\gamma_{21} &= \theta_{12} + \frac{\boldsymbol{\theta}_2'\left[\boldsymbol{\zeta}_2(\mathbf{X}) - \frac{\boldsymbol{\beta}_2(\mathbf{X})\delta_1}{\text{var}\{\tilde{S}_2(\mathbf{X}; \tilde{\boldsymbol{\alpha}}_2)\}}\right]}{\text{var}(A) - \text{var}\{E(A|\mathbf{X})\}\rho_{21}^2}\\&+ \frac{(\theta_{11} - \tilde{\gamma}_{11})\left[\text{cov}(AD, A) - c\left[E\{\text{var}(A|\mathbf{X})\} + (1 - \rho_{21}^2)\text{var}\{E(A|\mathbf{X})\}\right] - \rho_{21}\rho_{22}\sqrt{\text{var}\{E(A|\mathbf{X})\}\text{var}\{E(AD|\mathbf{X})\}}\right]}{\text{var}(A) - \text{var}\{E(A|\mathbf{X})\}\rho_{21}^2}\end{aligned}$$

TABLE 1 Empirical results for estimators of causal effects with $P(A=1)=0.25$ under six methods of analysis based on correctly specified models.

			Minimal PS: $\mathbf{S}=\{S_1(\mathbf{Z}_1), S_2(\mathbf{Z}_2)\}'$				Expanded PS: $\mathbf{S}=\{S_1(\mathbf{X}), S_2(\mathbf{X})\}'$			
Method	Exposure	Effect	Ebias	ESE	RSE	ECP(%)	Ebias	ESE	RSE	ECP(%)
Data Generation Model 1										
Naive	D	θ_{11}	0.762	0.050	0.049	0.0	-	-	-	-
	A	θ_{12}	0.971	0.134	0.134	0.0	-	-	-	-
DG model	D	θ_{11}	-0.001	0.034	0.033	94.4	-	-	-	-
	A	θ_{12}	-0.004	0.076	0.077	94.8	-	-	-	-
Two PS Reg	D	θ_{11}	-0.022	0.042	0.042	92.0	-0.023	0.041	0.041	91.7
	A	θ_{12}	-0.008	0.084	0.085	94.8	-0.007	0.078	0.078	94.7
$\text{PS}_\text{I}+\text{PS}_\text{II}$	D	θ_{11}	-0.002	0.085	0.083	94.1	-0.003	0.064	0.063	94.3
	A	θ_{12}	-0.004	0.112	0.112	95.1	-0.005	0.080	0.081	95.2
$\text{PS}_\text{I}+\text{IPW}_\text{II}$	D	θ_{11}	-0.002	0.085	0.083	94.1	-0.003	0.064	0.063	94.3
	A	θ_{12}	-0.004	0.160	0.152	94.0	-0.006	0.138	0.130	93.1
$\text{PS}_\text{I}+\text{AIPW}_\text{II}$	D	θ_{11}	-0.002	0.085	0.083	94.1	-0.003	0.064	0.063	94.3
	A	θ_{12}	-0.004	0.092	0.091	94.8	-0.005	0.089	0.088	94.8
Data Generation Model 2										
Naive	D	θ_{11}	0.637	0.047	0.048	0.0	-	-	-	-
	A	θ_{12}	0.358	0.104	0.104	7.0	-	-	-	-
DG model	D	θ_{11}	0.000	0.040	0.040	95.0	-	-	-	-
	A	θ_{12}	0.003	0.076	0.078	95.0	-	-	-	-
Two PS Reg	D	θ_{11}	-0.003	0.041	0.041	94.9	-	-	-	-
	A	θ_{12}	0.003	0.076	0.078	95.0	-	-	-	-
$\text{PS}_\text{I}+\text{PS}_\text{II}$	D	θ_{11}	0.000	0.064	0.063	93.5	-	-	-	-
	A	θ_{12}	0.003	0.077	0.079	95.2	-	-	-	-
$\text{PS}_\text{I}+\text{IPW}_\text{II}$	D	θ_{11}	0.000	0.064	0.063	93.5	-	-	-	-
	A	θ_{12}	0.005	0.109	0.106	93.6	-	-	-	-
$\text{PS}_\text{I}+\text{AIPW}_\text{II}$	D	θ_{11}	0.000	0.064	0.063	93.5	-	-	-	-
	A	θ_{12}	0.003	0.089	0.088	94.5	-	-	-	-

DG model: Data generation model; Two PS Reg: Two-part propensity score regression adjustment; $\text{PS}_\text{I}+\text{PS}_\text{II}$: Using PS regression adjustment in Stage I and II; $\text{PS}_\text{I}+\text{IPW}_\text{II}$: Using PS regression adjustment in Stage I and IPW in Stage II; $\text{PS}_\text{I}+\text{AIPW}_\text{II}$: Using PS regression adjustment in Stage I and AIPW in Stage II.

and, evaluating at the Stage I limiting value $\tilde{\gamma}_{11}=\gamma_{11}^*$ obtained in Section C.1, we can obtain γ_{21} expressed as

$$\gamma_{21}=\theta_{12}+\frac{\boldsymbol{\theta}_2'\left[\boldsymbol{\zeta}_2(\mathbf{X})-\boldsymbol{\beta}_2(\mathbf{X})\rho_{21}\sqrt{\frac{\text{var}\{E(A|\mathbf{X})\}}{\text{var}\{\tilde{S}_2(\mathbf{X};\tilde{\alpha}_2)\}}}\right]}{\text{var}(A)-\text{var}\{E(A|\mathbf{X})\}\rho_{21}^2}$$
$$+\frac{(\theta_{11}-\gamma_{11}^*)\left[\text{cov}(AD,A)-c\left[E\{\text{var}(A|\mathbf{X})\}+(1-\rho_{21}^2)\text{var}\{E(A|\mathbf{X})\}\right]-\rho_{21}\rho_{22}\sqrt{\text{var}\{E(A|\mathbf{X})\}\text{var}\{E(AD|\mathbf{X})\}}\right]}{\text{var}(A)-\text{var}\{E(A|\mathbf{X})\}\rho_{21}^2}.$$

D | ADDITIONAL SIMULATION RESULTS

We consider two scenarios where the probability of exposure is set to $P(A=1)=0.25$ and 0.75. Tables 1, 2, and 3 provide empirical results for estimators of causal effects under $P(A=1)=0.25$. These results are consistent with those when $P(A=1)=0.5$. Misspecification of S_1 produces dose-effect bias and can also affect the Stage II estimate, whereas misspecification of S_2 produces substantial Stage II bias for PS regression and IPW. Provided that the Stage I estimator is consistent, the Stage II AIPW estimator remains consistent when either the Stage II propensity score model or the imputation model is correctly specified. Tables 4, 5, and 6 display the simulation results for $P(A=1)=0.75$. The findings again reflect similar patterns observed under $P(A=1)=0.5$ and $P(A=1)=0.25$. When S_1 is misspecified in the first stage, dose-effect estimation deteriorates, while the impact on Stage II estimation varies across methods and data-generating models.

TABLE 2 Empirical results for estimators of causal effects with $P(A=1)=0.25$ for two-part PS regression adjustment and two-stage analysis under model misspecification.

Method	Exposure	Effect	Misspecified S_1				Misspecified S_2			
			Ebias	ESE	RSE	ECP(%)	Ebias	ESE	RSE	ECP(%)
			Data Generation Model 1							
Two PS Reg	D	θ_{11}	0.082	0.047	0.048	60.2	-0.014	0.042	0.043	93.5
	A	θ_{12}	0.008	0.097	0.098	94.9	0.243	0.086	0.087	18.2
$PS_I + PS_{II}$	D	θ_{11}	0.349	0.090	0.088	2.6	-0.002	0.085	0.083	94.1
	A	θ_{12}	0.050	0.105	0.106	92.3	0.335	0.113	0.114	15.7
$PS_I + IPW_{II}$	D	θ_{11}	0.349	0.090	0.088	2.6	-0.002	0.085	0.083	94.1
	A	θ_{12}	0.135	0.140	0.134	77.3	0.338	0.126	0.126	24.1
			Data Generation Model 2							
Two PS Reg	D	θ_{11}	0.056	0.046	0.046	76.2	0.000	0.041	0.041	94.9
	A	θ_{12}	0.007	0.077	0.079	94.8	0.197	0.088	0.088	39.4
$PS_I + PS_{II}$	D	θ_{11}	0.282	0.070	0.069	2.5	0.000	0.064	0.063	93.5
	A	θ_{12}	0.019	0.081	0.083	94.2	0.482	0.093	0.094	0.1
$PS_I + IPW_{II}$	D	θ_{11}	0.282	0.070	0.069	2.5	0.000	0.064	0.063	93.5
	A	θ_{12}	0.044	0.103	0.100	91.2	0.484	0.102	0.101	0.1

S_1 represents the propensity score model for the continuous exposure D conditioned on $A=1$, given by $S_1 = E(D|A=1, \mathbf{Z}_1)$; S_2 represents the propensity score model for the binary exposure A, given by $S_2 = E(A|\mathbf{Z}_2)$.

TABLE 3 Empirical results for estimators of effects for both parts with $P(A=1)=0.25$ for applying PS regression adjustment in Stage I and AIPW in Stage II under model misspecification.

Misspecified Model	Exposure	Effect	Ebias	ESE	RSE	ECP(%)
			Data Generation Model 1			
S_1	D	θ_{11}	0.349	0.090	0.088	2.6
	A	θ_{12}	0.135	0.101	0.099	72.8
S_2	D	θ_{11}	-0.002	0.085	0.083	94.1
	A	θ_{12}	-0.003	0.090	0.089	94.7
$m_a(\mathbf{X};\boldsymbol{\theta})$	D	θ_{11}	-0.002	0.085	0.083	94.1
	A	θ_{12}	-0.001	0.096	0.094	94.7
$S_1 + m_a(\mathbf{X};\boldsymbol{\theta})$	D	θ_{11}	0.349	0.090	0.088	2.6
	A	θ_{12}	0.138	0.104	0.102	72.3
$S_2 + m_a(\mathbf{X};\boldsymbol{\theta})$	D	θ_{11}	-0.002	0.085	0.083	94.1
	A	θ_{12}	0.226	0.086	0.088	25.9
			Data Generation Model 2			
S_1	D	θ_{11}	0.282	0.070	0.069	2.5
	A	θ_{12}	0.042	0.093	0.091	91.8
S_2	D	θ_{11}	0.000	0.064	0.063	93.5
	A	θ_{12}	0.003	0.084	0.085	94.5
$m_a(\mathbf{X};\boldsymbol{\theta})$	D	θ_{11}	0.000	0.064	0.063	93.5
	A	θ_{12}	0.007	0.104	0.101	93.5
$S_1 + m_a(\mathbf{X};\boldsymbol{\theta})$	D	θ_{11}	0.282	0.070	0.069	2.5
	A	θ_{12}	0.046	0.102	0.099	90.1
$S_2 + m_a(\mathbf{X};\boldsymbol{\theta})$	D	θ_{11}	0.000	0.064	0.063	93.5
	A	θ_{12}	0.485	0.098	0.098	0.1

S_1 represents the propensity score model for the continuous exposure D conditioned on $A=1$, given by $S_1 = E(D|A=1, \mathbf{Z}_1)$; S_2 represents the propensity score model for the binary exposure A, given by $S_2 = E(A|\mathbf{Z}_2)$; $m_a(\mathbf{X};\boldsymbol{\theta})$ represents the imputation models for $a = 0, 1$.

E | ADDITIONAL INFORMATION ABOUT THE APPLICATION

E.1 | Effects of Prenatal Covariates on PAE

The estimated effects of baseline covariates on the semi-continuous exposure are given in Table 7.

TABLE 4 Empirical results for estimators of causal effects with $P(A = 1) = 0.75$ under six methods of analysis based on correctly specified models.

			Minimal PS: $\mathbf{S} = \{S_1(\mathbf{Z}_1), S_2(\mathbf{Z}_2)\}'$				Expanded PS: $\mathbf{S} = \{S_1(\mathbf{X}), S_2(\mathbf{X})\}'$			
Method	Exposure	Effect	Ebias	ESE	RSE	ECP(%)	Ebias	ESE	RSE	ECP(%)
			Data Generation Model 1							
Naive	D	θ_{11}	0.765	0.029	0.029	0.0	-	-	-	-
	A	θ_{12}	0.976	0.160	0.157	0.0	-	-	-	-
DG model	D	θ_{11}	-0.001	0.026	0.027	95.8	-	-	-	-
	A	θ_{12}	0.002	0.077	0.078	94.9	-	-	-	-
Two PS Reg	D	θ_{11}	0.013	0.033	0.033	92.3	0.013	0.031	0.031	92.3
	A	θ_{12}	0.007	0.084	0.085	95.0	0.007	0.078	0.079	94.8
$\text{PS}_\text{I} + \text{PS}_\text{II}$	D	θ_{11}	-0.001	0.048	0.048	95.5	-0.001	0.036	0.036	95.6
	A	θ_{12}	0.004	0.109	0.112	95.3	0.002	0.079	0.081	95.2
$\text{PS}_\text{I} + \text{IPW}_\text{II}$	D	θ_{11}	-0.001	0.048	0.048	95.5	-0.001	0.036	0.036	95.6
	A	θ_{12}	0.007	0.150	0.147	93.7	0.004	0.132	0.126	94.2
$\text{PS}_\text{I} + \text{AIPW}_\text{II}$	D	θ_{11}	-0.001	0.048	0.048	95.5	-0.001	0.036	0.036	95.6
	A	θ_{12}	0.001	0.083	0.085	95.2	0.001	0.084	0.085	95.1
			Data Generation Model 2							
Naive	D	θ_{11}	0.637	0.028	0.028	0.0	-	-	-	-
	A	θ_{12}	0.357	0.120	0.118	14.7	-	-	-	-
DG model	D	θ_{11}	-0.001	0.030	0.030	94.6	-	-	-	-
	A	θ_{12}	0.001	0.079	0.078	94.0	-	-	-	-
Two PS Reg	D	θ_{11}	0.001	0.030	0.030	95.0	-	-	-	-
	A	θ_{12}	0.001	0.079	0.078	94.0	-	-	-	-
$\text{PS}_\text{I} + \text{PS}_\text{II}$	D	θ_{11}	-0.002	0.037	0.036	94.5	-	-	-	-
	A	θ_{12}	0.001	0.080	0.079	94.0	-	-	-	-
$\text{PS}_\text{I} + \text{IPW}_\text{II}$	D	θ_{11}	-0.002	0.037	0.036	94.5	-	-	-	-
	A	θ_{12}	0.002	0.111	0.105	92.8	-	-	-	-
$\text{PS}_\text{I} + \text{AIPW}_\text{II}$	D	θ_{11}	-0.002	0.037	0.036	94.5	-	-	-	-
	A	θ_{12}	0.001	0.091	0.087	93.1	-	-	-	-

DG model: Data generation model; Two PS Reg: Two-part propensity score regression adjustment; $\text{PS}_\text{I} + \text{PS}_\text{II}$: Using PS regression adjustment in Stage I and II; $\text{PS}_\text{I} + \text{IPW}_\text{II}$: Using PS regression adjustment in Stage I and IPW in Stage II; $\text{PS}_\text{I} + \text{AIPW}_\text{II}$: Using PS regression adjustment in Stage I and AIPW in Stage II.

E.2 | Details of Multiple Imputation Procedure

In our data application, the Detroit dataset contains missing values in the outcome and covariates. 181 women have at least one missing value (41 with missing WISC-III and 159 with at least one missing covariate, with overlap between these groups). We address this by generating 20 multiply imputed datasets using an imputation model including all variables; multiple imputation relies on the assumption that data are missing at random (MAR), which means the probability of missingness may depend on observed data but not on the unobserved values themselves. Following this assumption, we use the `mice` package in R to perform multiple imputation by chained equations (MICE) and generate 20 imputed datasets [2]. The imputation model included all variables used in the outcome model, the two propensity score models, and several auxiliary variables associated with the outcome or with missingness [3, 4].

Specifically, the imputation model included the outcome variable: the WISC-III Full Scale IQ measured at child age 7; the exposure variables: drinking status and average daily absolute alcohol consumption (AA/day) during pregnancy; and all covariates used in the propensity score models. In addition, auxiliary variables, which are the proportion of drinking days and the amount of alcohol consumed per drinking occasion across pregnancy are included. As shown in Li et al.[5], these measures of prenatal alcohol exposure reflect distinct patterns of drinking behaviour and have differing impacts on children's cognition; they also exhibit different relationships with covariates when constructing propensity score models. Therefore, including them as auxiliary variables enhances the plausibility of the MAR assumption and improves the accuracy of imputed values [3].

Two binary variables, child's gender and mother's marital status, are imputed using logistic regression. All other variables are continuous and imputed using predictive mean matching (PMM). The default chained equations algorithm in `mice` is run for 5 iterations per imputation. We set the random seed to 391 and use the same 20 imputed datasets for all application analyses.

TABLE 5 Empirical results for estimators of causal effects with $P(A = 1) = 0.75$ for two-propensity-score regression adjustment and two-stage analysis under model misspecification.

			Misspecified S_1				Misspecified S_2			
Method	Exposure	Effect	Ebias	ESE	RSE	ECP(%)	Ebias	ESE	RSE	ECP(%)
			Data Generation Model 1							
Two PS Reg	D	θ_{11}	0.206	0.037	0.037	0.0	0.007	0.032	0.033	94.8
	A	θ_{12}	0.080	0.098	0.099	88.0	0.255	0.085	0.087	15.2
$PS_I + PS_{II}$	D	θ_{11}	0.350	0.050	0.051	0.0	-0.001	0.048	0.048	95.5
	A	θ_{12}	0.134	0.109	0.112	78.5	0.344	0.110	0.114	13.2
$PS_I + IPW_{II}$	D	θ_{11}	0.350	0.050	0.051	0.0	-0.001	0.048	0.048	95.5
	A	θ_{12}	0.053	0.150	0.147	90.7	0.347	0.118	0.123	19.6
			Data Generation Model 2							
Two PS Reg	D	θ_{11}	0.111	0.036	0.036	13.6	-0.001	0.031	0.030	94.7
	A	θ_{12}	0.015	0.081	0.079	93.7	0.195	0.082	0.082	34.0
$PS_I + PS_{II}$	D	θ_{11}	0.285	0.038	0.040	0.0	-0.002	0.037	0.036	94.5
	A	θ_{12}	0.037	0.083	0.082	91.8	0.481	0.093	0.094	0.1
$PS_I + IPW_{II}$	D	θ_{11}	0.285	0.038	0.040	0.0	-0.002	0.037	0.036	94.5
	A	θ_{12}	0.014	0.113	0.107	92.5	0.483	0.100	0.101	0.3

S_1 represents the propensity score model for the continuous exposure D conditioned on $A = 1$, given by $S_1 = E(D|A = 1, \mathbf{Z}_1)$; S_2 represents the propensity score model for the binary exposure A, given by $S_2 = E(A|\mathbf{Z}_2)$.

TABLE 6 Empirical results for estimators of effects for both parts with $P(A = 1) = 0.75$ for applying PS regression adjustment in Stage I and AIPW in Stage II under model misspecification.

Misspecified Model	Exposure	Effect	Ebias	ESE	RSE	ECP(%)
			Data Generation Model 1			
S_1	D	θ_{11}	0.350	0.050	0.051	0.0
	A	θ_{12}	0.048	0.087	0.088	91.8
S_2	D	θ_{11}	-0.001	0.048	0.048	95.5
	A	θ_{12}	0.002	0.082	0.083	95.0
$m_a(\mathbf{X};\boldsymbol{\theta})$	D	θ_{11}	-0.001	0.048	0.048	95.5
	A	θ_{12}	0.005	0.087	0.088	94.9
$S_1 + m_a(\mathbf{X};\boldsymbol{\theta})$	D	θ_{11}	0.350	0.050	0.051	0.0
	A	θ_{12}	0.051	0.091	0.091	90.5
$S_2 + m_a(\mathbf{X};\boldsymbol{\theta})$	D	θ_{11}	-0.001	0.048	0.048	95.5
	A	θ_{12}	0.231	0.079	0.082	18.4
			Data Generation Model 2			
S_1	D	θ_{11}	0.285	0.038	0.040	0.0
	A	θ_{12}	0.013	0.092	0.089	93.3
S_2	D	θ_{11}	-0.002	0.037	0.036	94.5
	A	θ_{12}	0.001	0.086	0.084	94.2
$m_a(\mathbf{X};\boldsymbol{\theta})$	D	θ_{11}	-0.002	0.037	0.036	94.5
	A	θ_{12}	0.005	0.105	0.100	93.2
$S_1 + m_a(\mathbf{X};\boldsymbol{\theta})$	D	θ_{11}	0.285	0.038	0.040	0.0
	A	θ_{12}	0.017	0.107	0.102	92.7
$S_2 + m_a(\mathbf{X};\boldsymbol{\theta})$	D	θ_{11}	-0.002	0.037	0.036	94.5
	A	θ_{12}	0.484	0.096	0.097	0.1

S_1 represents the propensity score model for the continuous exposure D conditioned on $A = 1$, given by $S_1 = E(D|A = 1, \mathbf{Z}_1)$; S_2 represents the propensity score model for the binary exposure A, given by $S_2 = E(A|\mathbf{Z}_2)$; $m_a(\mathbf{X};\boldsymbol{\theta})$ represents the imputation models for $a = 0, 1$.

After imputation, the analysis under the proposed methods is described in Section 6 of the main text. The resulting point estimates and standard errors are then combined using Rubin's Rules to obtain valid overall estimates and associated uncertainty [6].

TABLE 7 Estimated effects of prenatal covariates on the continuous and binary exposure components

	$E(D \mid A=1,\mathbf{X})$		$P(A=1 \mid \mathbf{X})$	
	Estimate	Standard error	Estimate	Standard error
Intercept	-4.183	1.003	-0.965	1.926
Smoking during pregnancy (cigarettes/day)	0.016	0.007	0.088	0.025
Socioeconomic status at birth	-0.007	0.003	-0.003	0.005
Child's gender (female)	-0.165	0.165	-0.608	0.314
Mother's age at delivery	0.057	0.018	0.056	0.036
Prenatal Beck score	0.026	0.011	0.026	0.023
HOME score at infancy	-0.004	0.019	0.039	0.036
Biological mother's PPVT	-0.010	0.007	0.001	0.014
Biological mother's marital status	-0.042	0.276	-0.103	0.487
Prenatal cocaine exposure (days/month)	-0.061	0.038	-0.102	0.070
Prenatal marijuana exposure (days/month)	-0.012	0.032	0.220	0.198
Prenatal opiate exposure (days/month)	0.193	0.079	0.057	0.253
Number of prenatal visits	-0.121	0.032	-0.021	0.061
Biological mother's education (years)	0.098	0.059	-0.041	0.115
Parity	-0.003	0.095	-0.471	0.220
Gravidity	0.036	0.057	0.271	0.145
Gestational age at screening	0.018	0.013	-0.010	0.025

E.3 | Details of Method Implementation in Application

E.3.1 | Conventional Covariate Regression Adjustment

Let A be a binary exposure representing whether the mother drinks during pregnancy, T denote the absolute alcohol consumption per day (in ounces), and $D = \log T$ represent the logarithm of daily alcohol consumption if $A = 1$. The vector of all confounders is denoted by $\mathbf{X}$. The regression model we consider is:

$$E(Y_i \mid A_i, D_i, \mathbf{X}_i) = \theta_0 + \theta_{11}A_i(D_i - c) + \theta_{12}A_i + \mathbf{X}_i'\boldsymbol{\theta}_2,$$

where c is a fixed constant representing the mean logarithm of daily alcohol consumption (in ounces). In this analysis, we set $c = -2.31$ which corresponds to the empirical mean value of D among mothers in this study who reported drinking.

E.3.2 | Two-Part Propensity Score Regression Adjustment

Firstly, we define two propensity score models:

$$S_{i1} = E(D \mid A=1, \boldsymbol{X}_i) = \alpha_{10} + \boldsymbol{\alpha}_{11}\boldsymbol{X}_i,$$

$$S_{i2} = E(A \mid \boldsymbol{X}_i) = \text{expit}(\alpha_{20} + \boldsymbol{\alpha}_{21}\boldsymbol{X}_i).$$

Then the two–part PS regression adjustment is specified by the linear model

$$E(Y_i \mid A_i, D_i, \mathbf{S}_i) = \eta_0 + \eta_{11}A_i(D_i - c) + \eta_{12}A_i + \eta_{21}S_{i1} + \eta_{22}S_{i2}$$

where $\mathbf{S}_i = (S_{i1}, S_{i2})'$.

E.3.3 | The Two-Stage Approach

We assess the causal effects with a semi-continuous exposure by a two-stage approach. In the first stage, we aim to obtain the effect of the logarithm of the absolute alcohol consumption among exposed by propensity score regression adjustment. Then we treat $\hat{\gamma}_{11}A(D-c)$ as an offset term where $\hat{\gamma}_{11}$ is the estimated causal effect of alcohol consumption among exposed. In stage II, we apply propensity score regression adjustment, IPW, and AIPW approaches to find the effect of prenatal drinking status.

TABLE 8 Estimated effects of drinking status and log prenatal absolute alcohol consumption per day with quadratic propensity score terms

	$D \mid A = 1$		A	
Method	Estimate	Standard error	Estimate	Standard error
Two–PS regression	-0.207	0.541	-0.110	1.622
$\mathrm{PS_I} + \mathrm{PS_{II}}$	-0.062	0.536	-0.032	1.627
$\mathrm{PS_I} + \mathrm{IPW_{II}}$	–	–	-0.052	1.850
$\mathrm{PS_I} + \mathrm{AIPW_{II}}$	–	–	-0.052	2.099

Two–PS regression: two-dimensional PS regression adjustment; $\mathrm{PS_I} + \mathrm{PS_{II}}$: PS regression adjustment in Stages I and II; $\mathrm{PS_I} + \mathrm{IPW_{II}}$: PS regression in Stage I and IPW in Stage II; $\mathrm{PS_I} + \mathrm{AIPW_{II}}$: PS regression in Stage I and AIPW in Stage II.

TABLE 9 Estimated subgroup effects of drinking status and log prenatal absolute alcohol consumption per day with quadratic propensity score terms, with effect modifier $V = I(\text{maternal age} > 30)$

		$D \mid A = 1$		A	
Method	Subgroup	Estimate	Standard error	Estimate	Standard error
Two–PS regression	Younger ($V = 0$)	0.103	0.594	0.754	1.848
	Older ($V = 1$)	-0.986	1.209	-2.586	4.738
$\mathrm{PS_I} + \mathrm{PS_{II}}$	Younger ($V = 0$)	0.251	0.587	0.593	1.649
	Older ($V = 1$)	-0.967	1.204	-1.526	2.222
$\mathrm{PS_I} + \mathrm{IPW_{II}}$	Younger ($V = 0$)	–	–	0.983	2.128
	Older ($V = 1$)	–	–	-2.271	5.188
$\mathrm{PS_I} + \mathrm{AIPW_{II}}$	Younger ($V = 0$)	–	–	0.452	2.777
	Older ($V = 1$)	–	–	-0.705	3.448

$V = I(\text{maternal age} > 30)$. Younger ($V = 0$) and older ($V = 1$) rows report subgroup-specific dose–response and reference-dose effects.

E.4 | Sensitivity Analysis with Quadratic Propensity Score Terms

To assess sensitivity to the assumption that the propensity scores enter the adjustment models linearly, we refit the application models including quadratic terms of the propensity scores: S_1^2 and S_2^2 in the two–part PS regression; S_1^2 in Stage I and S_2^2 in Stage II for $\mathrm{PS_I}+\mathrm{PS_{II}}$; and S_1^2 in Stage I only for $\mathrm{PS_I}+\mathrm{IPW_{II}}$ and $\mathrm{PS_I}+\mathrm{AIPW_{II}}$, whose Stage II uses the propensity score through the weights. Standard errors are obtained from the joint sandwich variance estimator of Section 3.3 of the main text, extended to include the additional derivative terms induced by the quadratic propensity score columns, and estimates are combined across the 20 multiply imputed datasets by Rubin's rules. Table 8 reports the results for the homogeneous specification and Table 9 for the heterogeneous specification with effect modifier $V = I(\text{maternal age} > 30)$. In both cases the estimates are broadly consistent with those obtained under the linear specification (Tables 7 and 8 of the main text), with a small loss of precision in the weighting-based estimators reflecting the additional parameters, supporting the adequacy of the linear propensity score specification in this application.

REFERENCES

1. Rosenbaum PR, Rubin DB. The central role of the propensity score in observational studies for causal effects. *Biometrika.* 1983;70(1):41–55.
2. Van Buuren S, Groothuis-Oudshoorn K. mice: Multivariate imputation by chained equations in R. *Journal of Statistical Software.* 2011;45:1–67.
3. White IR, Royston P, Wood AM. Multiple imputation using chained equations: issues and guidance for practice. *Statistics in Medicine.* 2011;30(4):377–399.
4. Van Buuren S, Van Buuren S. *Flexible imputation of missing data*. 10. CRC press Boca Raton, FL, 2012.
5. Li K, Akkaya-Hocagil T, Cook RJ, et al. Use of Generalized Propensity Scores for Assessing Effects of Multiple Exposures. *Statistics in Biosciences.* 2023:1–30.
6. Rubin DB. Inference and missing data. *Biometrika.* 1976;63(3):581–592.
